# *ent*-Pimarane and *ent*-Kaurane Diterpenes from *Aldama discolor* (Asteraceae) and Their Antiprotozoal Activity

**DOI:** 10.3390/molecules21091237

**Published:** 2016-09-15

**Authors:** Mauro S. Nogueira, Fernando B. Da Costa, Reto Brun, Marcel Kaiser, Thomas J. Schmidt

**Affiliations:** 1 Institute of Pharmaceutical Biology and Phytochemistry (IPBP), University of Münster, PharmaCampus Corrensstraße 48, Münster D-48149, Germany; nogueira@uni-muenster.de; 2 AsterBioChem Research Team, Laboratory of Pharmacognosy, School of Pharmaceutical Sciences of Ribeirão Preto, USP, Av. do Café s/n, Ribeirão Preto-SP 14040-903, Brazil; febcosta@fcfrp.usp.br; 3 Swiss Tropical and Public Health Institute (Swiss TPH), Socinstr. 57, Basel CH-4051, Switzerland; reto.brun@unibas.ch (R.B.); marcel.kaiser@unibas.ch (M.K.); 4 University of Basel, Petersplatz 1, Basel CH-4003, Switzerland

**Keywords:** *Aldama discolor*, *Viguiera discolor*, Asteraceae, *ent*-pimarane, *ent*-kaurane, antiprotozoal activity, *Trypanosoma brucei rhodesiense*, *Trypanosoma cruzi*, *Leishmania donovani*, *Plasmodium falciparum*

## Abstract

*Aldama discolor* (syn.*Viguiera discolor*) is an endemic Asteraceae from the Brazilian “Cerrado”, which has not previously been investigated for its chemical constituents and biological activity. Diterpenes are common secondary metabolites found in *Aldama* species, some of which have been reported to present potential antiprotozoal and antimicrobial activities. In this study, the known *ent*-3-*α*-hydroxy-kaur-16-en-18-ol (**1**), as well as three new diterpenes, namely, *ent*-7-oxo-pimara-8,15-diene-18-ol (**2**), *ent*-2*S*,4*S*-2-19-epoxy-pimara-8(3),15-diene-7*β*-ol (**3**) and *ent*-7-oxo-pimara-8,15-diene-3*β*-ol (**4**), were isolated from the dichloromethane extract of *A. discolor* leaves and identified by means of MS and NMR. The compounds were assayed in vitro against *Trypanosoma brucei rhodesiense*, *T. cruzi* and *Leishmania donovani*, *Plasmodium falciparum* and also tested for cytotoxicity against mammalian cells (L6 cell line). The *ent*-kaurane **1** showed significant in vitro activity against both *P. falciparum* (IC${}_{50}$ = 3.5 μM) and *L. donovani* (IC${}_{50}$ = 2.5 μM) and *ent*-pimarane **2** against *P. falciparum* (IC${}_{50}$ = 3.8 μM). Both compounds returned high selectivity indices (SI >10) in comparison with L6 cells, which makes them interesting candidates for in vivo tests. In addition to the diterpenes, the sesquiterpene lactone budlein A (**5**), which has been reported to possess a strong anti-*T. b. rhodesiense* activity, was identified as major compound in the *A. discolor* extract and explains its high activity against this parasite (100% growth inhibition at 2 μg/mL).

## 1. Introduction

*Aldama* La Llave [1], Syn.*Viguiera* Kunth, is a large American genus with about 180 species, which are widespread from North America until Argentina, but mostly concentrated in Mexico, the Andes and upland areas of Brazil [2]. It must be noted that most of this work refers to *Aldama* as *Viguiera*, which has been re-classified in 2011 in the course of a taxonomic reinvestigation of the genus [1]. In the last two decades, several species of *Aldama* have been chemically investigated with numerous articles focusing on Central and South American species, but not many have been published on the Brazilian species. For *A. discolor* (previously referred to as *V. discolor*), a species occurring in the Brazilian “Cerrado” habitats, neither the chemical investigation of its secondary metabolites nor investigations on its biological activity have been described before.

The main classes of secondary metabolites known from the genus so far are terpenoids, i.e., sesquiterpene lactones (STLs), represented mainly by germacranolides, heliangolides and furanoheliangolides [3,4,5,6,7], as well as diterpenes, whose presence has been largely described in the genus. Among the diterpenes, *ent*-kauranes are the most common types [3,6,8,9,10,11], although *ent*-beyerane, *ent*-atisane [12,13] and trachylobane [14] have also been described. Pimarane diterpenes have been found only in *A. pinnatilobata* [15] and *A. arenaria* [16,17].

Several biologically-active secondary metabolites have been described as constituents of different species of *Aldama*. Although only a few *Aldama* species have been investigated, the bioactivities found are diverse, such as trypanocidal [10,17], cytotoxic [18], antimicrobial [19,20], anti-inflammatory [21], insecticide [22,23] and inhibition of vascular smooth muscle contractility [24].

Eukaryotic unicellular (“protozoan”) parasites are known to cause infectious diseases, such as Human African trypanosomiasis (HAT), Chagas disease, leishmaniasis and malaria. These infections are responsible for a large number of deaths and a considerable contribution to the world-wide burden of disease, especially in impoverished regions of tropical countries. The treatment of these diseases is hampered, in many cases, by the use of old drugs, with low safety, by insufficient availability of drugs or complicated treatment regimens, or by the emergence of parasite resistance against the few existing therapeutics. Hence, there is an urgent need to find new drug candidates to be developed into new safe, efficient and available therapies. Natural products (NP) from plants of the Asteraceae family, especially sesquiterpene lactones (STLs) and diterpenes, have frequently been reported to present antiparasitic properties [25].

Previous reports on the antiprotozoal activities of some *Aldama* species highlighted active NP, such as *ent*-kaur-16-en-19-oic acid [10] against *Trypanosoma cruzi* in vitro, *ent*-15-pimarene-8*β*,19-diol [17] against *Trypanosoma cruzi* in vivo and budlein A against *T. brucei rhodesiense* in vitro [26]. On the basis of this reported biological potential, the present work, which is part of a larger study of in vitro screening of various Asteraceae from Brazilian “Cerrado” for antiprotozoal activity [27,28], reports on terpenoid constituents isolated from the dichloromethane extract of *Aldama discolor* and their antiprotozoal activity.

## 2. Results

### 2.1. Antiprotozoal Activity of A. discolor Crude Extracts

As part of an extensive screening of Asteraceae from the Brazilian “Cerrado”, extracts obtained from the leaves of *Aldama discolor* with solvents of different polarity (n-hexane (Hex), dichloromethane (DCM), ethyl acetate (EtOAc) and methanol (MeOH)) were tested for in vitro activity against *Trypanosoma brucei rhodesiense* (bloodstream trypomastigotes), *T. cruzi* (intracellular amastigotes), *Leishmania donovani* (axenic amastigotes) and *Plasmodium falciparum* (intraerythrocytic forms). Each extract was tested at two concentrations, 2 and 10 μg/mL, for growth inhibitory activity (% GI) against the various parasites. The results are presented in Table 1. It becomes obvious that all extracts presented a considerable activity against *T. b. rhodesiense*, which showed 100% growth inhibition already at 2 μg/mL of the DCM, the EtOAc, as well as the MeOH extract. *L. donovani* and *P. falciparum* were less strongly affected by the low concentration, but also quite susceptible to 10 µg/mL of all extracts, while *T. cruzi* was not affected by any of the extracts. For isolation and characterization of the potentially active chemical constituents, the DCM extract was chosen.

### 2.2. Isolation and Characterization of the Compounds

**Figure 1:**
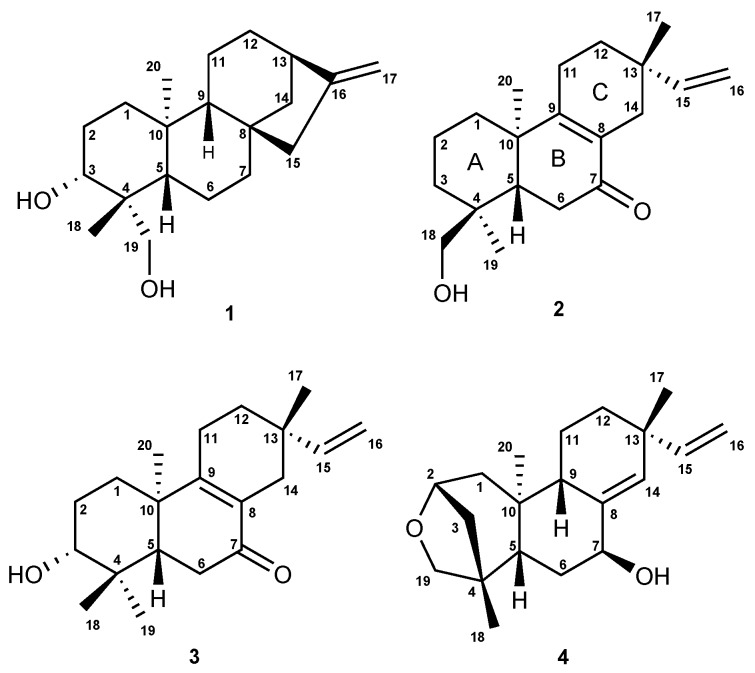
Chemical structures of diterpenes **1**–**4**.

Separation of the DCM extract of *Aldama discolor* leaves afforded the known *ent*-3-*α*-hydroxy-kaur- 16-en-18-ol (**1**) [29] and three diterpenes of the *ent*-pimarane type (**2** **4** (Figure 1)), which have not previously been described. Besides the diterpenes, a fraction consisting of almost pure budlein A (**5**) was obtained, which was identified by its HRESI mass spectrum obtained in direct comparison with an authentic reference sample by UHPLC/+ESI-QqTOF/MS (Figure S1, Supplementary Materials).

Diterpene **1** (*ent*-3-*α*-hydroxy-kaur-16-en-18-ol) was unambiguously identified by its NMR spectra (for ${}^{1}$H and ${}^{13}$C-NMR data, see Table 2) and exact mass data ([M + H]${}^{+}$ at *m/z* 305.2494; calculated for C${}_{20}$H${}_{31}$O${}_{2}$: *m/z* 305.2481; Figure S2, Supplementary Materials) in comparison with published data [29]. The relative configuration of **1** was furthermore confirmed by a 2D 1H/1H NOESY spectrum (Figure S8, Supplementary Materials), and the assignment of its absolute configuration was confirmed by its negative optical rotation (${\left[\alpha \right]}_{D}^{24}$ = −20.6), which was in agreement with the literature [29,30].

In case of Compounds **2**, **3** and **4**, the presence of further diterpenes was clear from the exact mass data, as well as the ${}^{13}$C-NMR and HSQC spectra (Figures S11, S12, S18, S19, S26 and S27, Supplementary Materials) from which the number of rings and double bonds could be deduced by means of the hydrogen deficiency index, which pointed towards the presence of a pimarane-type carbon skeleton. Their affiliation to the pimar-15-ene type became clear in all three cases by the presence of an ABXspin system in their ${}^{1}$H-NMR spectra (Figures S10, S17 and S25, Supplementary Materials) and the carbon resonances of C-15 and C-16 in the ${}^{13}$C-NMR spectral data due to a terminal vinyl group (Table 2).

**Figure 2:**
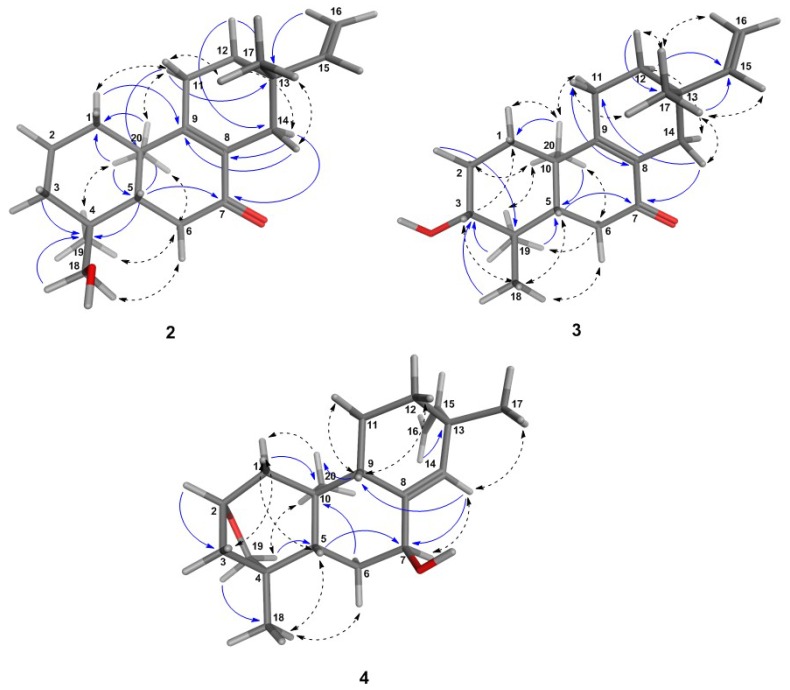
3D-structures of the most energetically-favorable conformers for the *ent*-pimaranes **2**–**4** obtained after a low mode molecular dynamics (LMD) conformation search. Distances (≤3.0 Å) between the protons in the molecular model were in agreement with the experimental NOE effects, as shown by the NOESY correlations (dashed arrows). HMBC key correlations are also shown (blue arrows).

Compound **2** was obtained as a colorless gum, ${\left[\alpha \right]}_{D}^{24}$: +12.4 (CHCl${}_{3}$), UV$\lambda_{max}$: 220 and 254 nm. The +ESI-MS spectrum returned a quasi-molecular ion at *m/z* 303.2304 [M + H]${}^{+}$, calculated for C${}_{20}$H${}_{31}$O${}_{2}$: *m/z* 303.2346 (Figure S9, Supplementary Materials). The carbon resonance at *δ* 199.8 (Table 2) indicated the presence of one ketone group. The deshielded carbon resonances of two quaternary sp${}^{2}$ carbons (C-8 at *δ* 129.1 and C-9 at *δ* 165.8) indicated conjugation of a tetra-substituted C=C double bond with the carbonyl group [31], whose position was confirmed at C-7 by the ${}^{1}$H–${}^{13}$C long-range correlations between the protons H-14*β*/H-14*α* and the carbonyl carbon (Figure 2; Figure S13, Supplementary Materials). The presence of a hydroxyl group as already suggested by the mass spectrum showing a fragment at *m/z* 285.2222 [M + H − H${}_{2}$O]${}^{+}$ in the MS2 spectra, was confirmed by the NMR spectra, and the position was located at C-18 by comparison of the ${}^{13}$C-NMR shift value (*δ* 70.9) with literature data, which have shown that hydroxylation at C-18 returns higher resonances (*δ* 71–75) than that at C-19 (*δ* 64–65) [30,31]. Moreover, hydroxylation at the equatorial substituent C-18 causes a shielding effect at C-5, as becomes clear when **2** is compared with **1**, where the hydroxylation is at C-19 (C-5: *δ* 43.2 in Compound **2** as compared to *δ* 55.9 in Compound **1**, Table 2). The stereochemistry at the C ring was solved based on the correlations in a NOESY spectrum (Figure S15, Supplementary Materials), from which it became clear that the methyl group C-17 is in the *β* and the vinyl group, hence in the *α*-position at C-13 (Figure 2), so that the presence of an iso-pimarane was excluded and the compound unambiguously assigned the structure of a pimarane.

Compound **3** was obtained as a white amorphous solid, ${\left[\alpha \right]}_{D}^{24}$: −49.2, CHCl${}_{3}$, UV$\lambda_{max}$: 220 nm and 250 nm, and found to represent an isomer of Compound **2**, as shown by its +ESI-QqTOF MS spectrum with a quasi-molecular ion at *m/z* 303.2332 [M + H]${}^{+}$, calculated for C${}_{20}$H${}_{31}$O${}_{2}$: *m/z* 303.2346 (Figure S16, Supplementary Materials). The NMR spectra were also very similar to those of **2**, and the only major differences observed pointed towards a changed position of the OH group, which could be located at C-3, as indicated by the carbon resonance at *δ* 78.1 and the methine proton signal at *δ* 3.29 (Table 2; Figure S17, Supplementary Materials), which was correlated with H-2*α* at *δ* 1.69 and H-2*β* at *δ* 1.77 in the COSY spectrum (Figure S21, Supplementary Materials). The relative *α*-orientation of the C-3-OH group follows from the coupling constants of H-3, which appears as a dd with a large (11.7 Hz) and a smaller (4.4 Hz) coupling constant with the neighboring protons at C-2, clearly showing that H-4 is in the axial and the OH group, hence in the equatorial (in this case *α*-). All observed NOEs (Figure 2) were in agreement with the 3D structure obtained by molecular modeling, confirming the relative configuration as depicted in Figure 1.

**Figure 3:**
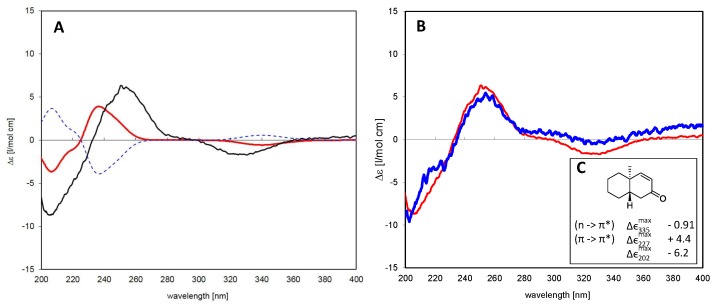
(**A**) Time-dependent density functional (TDDFT) calculated CD spectra for Compound **3** as a normal pimarane (blue, dashed) and *ent*-pimarane (red) in comparison with the experimental spectrum of the isolated compound (black); (**B**) comparison of CD spectra of Compounds **1** (blue) and **3** (red). Both compounds show essentially the same Cotton effects, so that they belong to the same series of diterpenes; (**C**) Correlation between the Cotton effect sign of a model enone in heptane that corresponds to the same absolute configuration as that observed in Compounds **1** and **3** (adapted from Lightner et al. [32]).

The absolute configuration of the closely-related isomers **2** and **3** was solved by comparison of their CD spectra with the theoretical spectrum simulated by a time-dependent density functional (TDDFT) calculation of Compound **3** and by comparison of the experimental CD spectra with literature data for structurally-related model compounds. Figure 3A shows a comparison of the experimental CD spectrum of **3** and the theoretical spectra simulated for the two enantiomers of this diterpene, i.e., as a normal and as an *ent*-pimarane. It becomes clear that the experimental curve with intense positive and negative Cotton effects (CEs) at 254 and 330 nm matches the simulated spectrum for the *ent*-pimarane quite well. Moreover, all three observed experimental CEs of both **2** and **3** (Figure 3B) are also in very good agreement with published data for a model compound containing the same chromophore in a very similar chiral environment corresponding to the *ent*-geometry [32] (Figure 3C), so that the assignment of the absolute configuration of both compounds to the *ent*-pimarane series is unambiguous. This is also in agreement with the literature on other diterpenes in *Aldama* species, where pimaranes or the normal series have not been found. Compound **2** hence represents *ent*-7-oxo-pimara-8,15-diene-18-ol, and Compound **3** is assigned the structure of *ent*-7-oxo-pimara-8,15-diene-3*β*-ol. Both compounds have not been described as natural or synthetic products before, to the best of our knowledge.

Compound **4** was obtained as a white amorphous solid, ${\left[\alpha \right]}_{D}^{25}$: −24.7 (CHCl${}_{3}$), UV$\lambda_{max}$: 220 nm and revealed by its +ESI/QqTOF mass spectrum with a quasi-molecular ion at *m/z* 303.2369 [M + H]${}^{+}$, calculated for C${}_{20}$H${}_{31}$O${}_{2}$: *m/z* 303.2346 (Figure S24, Supplementary Materials) to be an isomer of **2** and **3**. However, major differences in the NMR spectra showed the absence of the conjugated enone partial structure observed in **2** and **3**. Instead, the resonances of a trisubstituted double bond with resonances at *δ* 140.1 (quaternary, C-8) and 133.9 (CH, C-14), whose position between C-8 and C-14 was confirmed by the observed ${}^{5}$*J* long-range couplings of the corresponding methine proton (H-14) with the protons at the vinyl methylene group (t, ${}^{5}J_{14,16a}$ = 1.7 Hz, ${}^{5}J_{14,16b}$ = 1.7 Hz). The position of the hydroxyl group at C-7 was deduced from the couplings of H-7 with the protons at C-6 (dd at *δ* 4.25, with ${}^{3}$*J*${}_{7,6a}$ = 6.8 Hz, ${}^{3}$*J*${}_{7,6b}$ = 3.8 Hz). Occurrence of two relatively small couplings of H-7 can only be explained if it is in an equatorial (in this case *α*-position), and the OH hence occupies the (axial) *β*-position at C-7. The fact that **4** is an isomer of **2** and **3**, but lacks the carbonyl double bond or any other additional double bonds besides those at C-8=C14 and C-15=C-16 indicates that the compound must contain an additional ring. The resonances of oxygenated methine and methylene carbons at *δ* 81.0 and 64.4 ppm (C-2 and C-19, respectively) indicate that both carbons are bound to the remaining oxygen atom, which must hence form an ether bridge between these two carbons. The presence of a tetrahydrofuran ring involving C-2,3,4 and 19, as well as the oxygen atom was unambiguously confirmed by the ${}^{1}$H/${}^{13}$C-correlations observed in the HMBC spectrum (Figure 2, Figure S28, Supplementary Materials). The stereochemistry in this part of the molecule, i.e., the relative orientation of C-3 and C-19 forming the cyclohexane and tetrahydrofuran rings, could be deduced from an NOE between the oxymethylene proton H-19a and the methyl group CH${}_{3}$-20 (Figure 2), which is only possible if they are both on the same side of the molecule. CH${}_{3}$-20 adopts an *α*-orientation in *ent*-pimaranes, so that the same must hold true for the C-19-O-C-2 bridge. The relative configuration in a major part of the molecule could be determined from further NOESY correlations (Figure 2; Figure S30, Supplementary Materials). The relative configuration at C-13 (i.e., assignment of a pimarane or iso-pimarane) in the case of Compound **4** could not be solved on grounds of the NOESY spectrum. However, it has been reported that pimara-8(14),15-dienes and their *ent*-analogues show a deshielded resonance of C-17 (*δ*C about 29 ppm) in comparison with the corresponding iso-pimarane and *ent*-isopimarane derivatives (*δ*C around 24 ppm) [31]. C-17 resonates at 29.2 ppm in Compound **4** so that it can be assigned the relative configuration of a pimarane/*ent*-pimarane, i.e., the same as in Compounds **2** and **3**. Due to the close structural similarity to the *ent*-pimaranes **2** and **3**, the absolute stereochemistry should be that of an *ent*-pimarane, as well. Compound **4** thus represents *ent*-2*S*,4*S*-2,19-epoxy-pimara-8(14),15-diene-7*β*-ol, a hitherto unknown natural product.

### 2.3. In Vitro Antiprotozoal Activities

The high activity of the crude extract against *T. b. rhodesiense* is easily explained by the presence of budlein A as a major constituent in the extract (see above and Figure S1, Supplementary Materials), which has previously been reported by our group to possess exceptionally strong activity against this pathogen in vitro [26]. However, in our ongoing investigations to predict the activity of compounds from Asteraceae, using chemometric methods ([27,28]; a full account of this study will be published separately), diterpenes, such as **1** and **2**, were also suggested as active constituents of the dichloromethane extract of *A. discolor*. Therefore, the isolated diterpenes were tested against *T. b. rhodesiense*, *T. cruzi*, *L. donovani* and *P. falciparum*, as well as for cytotoxicity against mammalian cells (L6 cell line) using established protocols [33]. Moreover, pimarane and kaurane-type diterpenes have previously been reported to have some antiprotozoal activity [17,34,35]. The resulting IC${}_{50}$ values are reported in Table 3. The highest antiprotozoal activities were obtained for *ent*-pimaranes **1** against both *P. falciparum* and *L. donovani* and **2** against *P. falciparum*, against which they also showed significant selectivity (selectivity indices (SI) all > 10). Their activity and selectivity against *T. b. rhodesiense* and *T. cruzi* were considerably lower.

## 3. Discussion

Compound **2** (*ent*-7-oxo-pimara-8,15-diene-18-ol) showed a relatively selective activity against *P. falciparum* and yielded the highest SI among the tested compounds (IC${}_{50}$ = 3.85 μM; SI = 12.55; Table 3). There are only two reports on the antiprotozoal activity of isopimaranes, which also addressed the evaluation of the activity against *P. falciparum* [34,35]. In the work of Asili et al. [34], 8(9),15-isopimaradien-3*β*-ol showed the most prominent in vitro antiplasmodial effect against *P. falciparum* strain 3D7 (IC${}_{50}$ = 24.5 μM). The study associated the antiplasmodial activity with a pronounced membrane-modifying effect on erythrocytes, e.g., to induce echinocytosis and cell lysis. The mentioned compound differed from the remaining compounds evaluated in the series in that it contained only a single oxygen at one end of the molecule, resulting in an amphiphilic character. In a similar manner, the congener 8(14),15-isopimaradien-3*β*,19-diol, in which the polar groups are located at one end of the molecule, induced echinocytosis at low concentrations and caused pronounced cell lysis, indicating, therefore, an indirect antiplasmodial effect [34]. This has also been highlighted in further studies with other NPs [36,37]. From this aspect, the *ent*-pimarane **2** with oxygen substituents at C-18 and C-7 is less likely to induce antiplasmodial effects in that manner in comparison with Compound **1**, which presents such an amphiphilic character. However, the latter showed very similar activity with that of the former (**1**, IC${}_{50}$ = 3.54 μM; **2**, IC${}_{50}$ = 3.85 μM), suggesting that the activity may not underlie such indirect effects on the erythrocyte host cell. Thus, to understand the mechanisms of action for the antiplasmodial and antileishmanial activities of these compounds, further investigations have to be carried out.

Within pimarane/isopimarane types, only 1,2,11-trihydroxypimara-8(14),15-diene and 1,11-dihydroxypimara-8(14),15-diene displayed relevant antiplasmodial activity with IC${}_{50}$ values of 27.4 μM/mL and 9.8 μM/mL, respectively, against the *P. falciparum* K-1 strain [35]. Thus, Compounds **1** and **2** are the most active diterpenes of these types against *P. falciparum* so far. Among kaurene and labdane-type diterpenes, stronger effects have been reported against *T. b. rhodesiense* (0.76–1.49 μM) [38]. 11*α*-Hydroxy-*ent*-16-kauren-15-one was notably active against *T.b. brucei* with an IC${}_{50}$ value of 0.761 μM and a quite low SI (3.2), albeit the cytotoxicity was determined with a human embryonic cell line (MRC-5) [38].

Some *ent*-pimaranes from *Aldama arenaria* (Asteraceae) were previously reported to show in vitro activity against trypomastigote forms of *T. cruzi* (Y strain). The compounds with the highest effects were *ent*-15-pimarene-8*β*,19-diol and 3*β*-acetoxy-*ent*-8(14),15-pimaradiene, with IC${}_{50}$ of 116.5 μM and 149.3 μM, respectively [17]. However, this level of activity, similar to that of the diterpenes **1** and **4** against *T. cruzi*, may be considered negligibly low. Although the IC${}_{50}$ of **2** and **3** were considerably lower, their activity is still much too low to consider them as useful hits against *T. cruzi*.

By now, there are not many reports on the antiprotozoal activity of pimarane/isopimarane and kaurane-type diterpenes [25]. In this study, not only the significant activity of *ent*-kaurane **1** against both *P. falciparum* and *L. donovani* and *ent*-pimarane **2** against *P. falciparum* was highlighted, but also their high SI ranging from 11–16. Therefore, these compounds can be considered as hits to be included in further steps of the investigation for their antiprotozoal activity, i.e., to test them against the parasites in in vivo models.

## 4. Materials and Methods

### General Procedures

Analytical TLC was developed on silica gel plates 60 F${}_{254}$ (Merck Chemicals GmbH, Darmstadt, Germany) with EtOAc-hexane 2:1 (1.5% acetic acid) as the mobile phase. TLC plates were observed under UV-light at wavelengths of 254 nm and 360 nm, then sprayed with anisaldehyde-sulfuric acid reagent and heated on a hot plate [39]. Preparative HPLC separations were undertaken with a Jasco (Gross-Umstadt, Germany) prep.HPLC system (pump: PU-2087 plus; diode array detector MD 2018 plus; column thermostat CO 2060 plus; autosampler AS 2055 plus; LC Net II ADC Chromatography Data Solutions; sample injection loop: 2000 μL). Separations were performed using a preparative reverse phase column Reprosil 100 C-18 (5 μm, 250 mm × 20 mm, Macherey-Nagel, Düren, Germany) with binary gradients of the mobile phase. A flow rate of 12 mL/min and a column-thermostat temperature of 40 ${}^{\circ }$C were used in all HPLC preparative separations. Chromatograms were recorded at 210–220, 254 and 280 nm. Optical rotations were recorded in CHCl${}_{2}$ on a Jasco P-2000 polarimeter. UV spectra were extracted from the HPLC-UV-DAD chromatograms. CD spectra were recorded with a Jasco J-815 CD-spectropolarimeter in MeOH.

## 5. Plant Material

Leaves of *Aldama discolor* (Baker) E.E.Schill. & Panero (Syn. *Viguiera discolor*) were collected at Sacramento-MG, Brazil (GPS: 20${}^{\circ }$00’26.1” S and 047${}^{\circ }$24’30.3” W) in November 2012. Plants were oven-dried at 40 ${}^{\circ }$C for 48 h. Voucher specimens were deposited under reference D.P.V.FALEIRO 01 at the Herbarium SPFR, University of São Paulo, Ribei$\tilde{r}$ao Preto-SP, and at the Herbarium HUSC, University of Santos, Santos-SP, Brazil, and were identified by Dr. Mara Angelina Galvão Magenta.

### Chromatographic Separation of the Dichloromethane Extract of Aldama discolor Leaves: Isolation of Compounds ***1**–**4***

Dried leaves of *A. discolor* (100 g) were powdered and extracted with CH${}_{2}$Cl${}_{2}$ (3 × 1 L, 3 × 24 h) by maceration at room temperature to obtain the crude extract (20 g) after solvent evaporation. This was suspended in MeOH–H${}_{2}$O (9:1, 300 mL) and partitioned firstly with hexane (3 × 300 mL). The MeOH–H${}_{2}$O phase was evaporated until full removal of the methanol. The remaining solution was partitioned with CH${}_{2}$Cl${}_{2}$ (3 × 250 mL) after adding water to an equal volume. After evaporation of the solvents, the hexane-soluble fraction (6.8 g), the H${}_{2}$O-soluble fraction (3 g) and the CH${}_{2}$Cl${}_{2}$-soluble fraction (9.6 g) were obtained. The CH${}_{2}$Cl${}_{2}$-soluble fraction (9 g) was separated on a silica gel column (800 g) packed with the initial mobile phase. The sample was eluted with a gradient of the mobile phase increasing the polarity of the mixture hexane-EtOAc (7:3, *v*/*v*), as follows: 2.5 L (7:3), 2 L (1:1), 2 L (7:3), followed by 1 L of EtOAc; flow: 0.8–1 mL/min. The first 1.5 L of eluent were collected as a single fraction, followed by 130 fractions of around 50 mL. These were combined according to their TLC profiles. After the collection of these fractions, the column was further washed with EtOAc (2 L) and furnished a last fraction (4.49 g) containing budlein A (**5**) as a major constituent. Fractions F${}_{66–88}$ (130 mg) and F${}_{89–102}$ (204 mg) yielded Compounds **1** (3.6 mg), **2** (7.4 mg), **3** (2.4 mg), and **4** (12.4 mg) after purification by preparative HPLC. The mobile phase optimized for the HPLC prep. consisted of acidified water with 0.01% TFA (trifluoroacetic acid) (A) and MeOH (B) in gradient conditions: 60%–80% of B (15 min), 80%–90% of B (15–30 min), 90%–100% of B (30–37 min), 100% of B (37–44 min) and two more minutes to return to the initial mobile phase. The retention times of Compounds **1**–**4** were 28.4 min, 36.6 min, 21.8 min and 23.2 min, respectively.

## 6. Chromatographic Analysis of the Plant Extract and Pure Compounds by UHPLC-MS

The plant extract and its fractions were dissolved in MeCN at a defined concentration (5 mg/mL) and analyzed with UHPLC/ESI-QTOF MS/MS. Chromatographic separations were performed on a Dionex Ultimate 3000 RS Liquid Chromatography System with a Dionex Acclaim RSLC 120, C18 column (2.1 × 100 mm, 2.2 μm) using a binary gradient (A: water with 0.1% formic acid; B: acetonitrile with 0.1% formic acid) at 0.8 mL/min: 0–9.5 min: linear from 5% B–100% B; 9.5–12.5 min: isocratic 100% B; 12.5–12.6 min: linear from 100% B down to 5% B; 12.6–15 min: isocratic 5% B. The injection volume was 5 μL. Eluted compounds were detected using a Dionex Ultimate DAD-3000 RS over a wavelength range of 200–400 nm and a Bruker Daltonics micrOTOF-QII quadrupole/time-of-flight mass spectrometer equipped with an Apollo electrospray ionization source in positive mode at 5 Hz over a mass range of *m/z* 50–1000 using the following instrument settings: nebulizer gas nitrogen, 5 bar; dry gas nitrogen, 9 L/min, 220 ${}^{\circ }$C; capillary voltage 4500 V; end plate offset −500 V; transfer time 70 μs; collision gas nitrogen; collision energy and collision RF settings were combined for each single spectrum of 1000 summations as follows: 250 summations with 20% base collision energy, 130 Vpp+ 250 summations with 100% base collision energy, 500 Vpp + 250 summations with 20% base collision energy and 130 Vpp + 250 summations with 100% base collision energy and 500 Vpp. Base collision energy was 50 eV for precursor ions with a *m/z* less than 500 and then linearly interpolated against *m/z* up to a maximum of 70 eV for precursor ions with a *m/z* of up to 1000. Internal dataset calibration (HPCmode) was performed for each analysis using the mass spectrum of a 10 mM solution of sodium formiate in 50% isopropanol that was infused during LC re-equilibration using a divert valve equipped with a 20-μL sample loop.

### 6.1. Structural Determination of the Isolated Compounds

#### 6.1.1. General

Structural elucidation was carried out using the mass spectrometry data of compounds and one-, as well as two-dimensional NMR techniques: ${}^{1}$H, ${}^{13}$C, HSQC, HMBC, COSY and NOESY. The NMR spectra were recorded on an Agilent DD2 600 MHz spectrometer. All spectra were obtained in CDCl${}_{3}$ at room temperature. They were referenced to the solvent signals of CDCl${}_{3}$ (${}^{1}$H; 7.260 ppm) and CDCl${}_{3}$ (${}^{13}$C; 77.000 ppm). Spectra were processed and evaluated using MestReNOVA v. 10 (Mestrelab Research, Santiago de Compostela, Spain). Full structural elucidation of new structures, e.g., to clarify the relative configuration, was aided by molecular modeling using the Molecular Operations Environment (MOE, 2011.10). Structures were drawn in the different stereoisomeric forms, and each one was submitted to energy minimization using the MMFF94X force field and default parameters from the software. Following, the structures were subjected to a conformational search, using the low mode molecular dynamics (LMD) method, as implemented in MOE and the MMFF94Xforce field. For this, all items were set as default with an RMS gradient of 0.01 kcal/mol and an RMS distance of 0.1 Å. The obtained conformers were output into an MDBfile, and the distances between specific protons in the structure were measured whether either for the conformer with the lowest energy or for all conformers within an energy window of 4 kcal/mol above the most energetically-favorable geometry. Observed distances between the protons (⩽3 Å) of the most energetically-favorable conformers in agreement with the presence of NOEs were compared with the experimental data from NOESY spectra for the determination of the final structure.

#### 6.1.2. Assignment of Absolute Configuration for *ent*-Pimaranes

In order to decide unambiguously whether the isolated pimarane-type compounds belong to the normal or enantio (*ent*-)pimarane series, CD spectra of Compounds **2** and **3** were recorded and related to the theoretical spectrum of **3**/*ent*-**3** calculated by time-dependent density functional theory (TDDFT) computations. To this end, a 3D molecular model of *ent*-**4** was generated and geometry optimized as described previously. After energy minimization of six low energy conformers obtained in an LMD conformation search (MOE) with Gaussian W03 [40] using the B3LYP density functional and a 6-31G(d,p) basis set, the populations of the six conformers were calculated using their energy differences and the Boltzmann equation. They were thus estimated to represent 25.4%, 24.8%, 21.1%, 17.7%, 6.1% and 4.9% of the overall conformational equilibrium. TD-DFT calculations were performed for the first four conformers using the same density functional and basis set as above and considering the 30 lowest-energy electronic transitions for each conformer. The resulting rotatory strength vectors were converted into a simulated CD spectrum for each molecule species as described previously, e.g., in [41,42]. The individual spectra were averaged according to the equilibrium percentages mentioned above, which resulted in the simulated spectrum shown in Figure 3.

### 6.2. Analytical Data for Compounds ***1**–**5***

*ent*-3-*α*-hydroxy-kaur-16-en-18-ol (**1**): white amorphous solid; ${\left[\alpha \right]}_{D}^{24}$: −20.6 (CDCl${}_{3}$); UV (H${}_{2}$O-MeCN) $\lambda_{max}$: 225. +ESI MS (*m/z*): 229.1961, 269.2245 [M + H − 2(H${}_{2}$O)]${}^{+}$, 287.2402 [M + H − H${}_{2}$O]${}^{+}$; 305.2494 [M + H]${}^{+}$; 327.2322 [M + Na]${}^{+}$; ${}^{1}$H and ${}^{13}$C-NMR (CDCl${}_{3}$), see Table 2. 

*ent*-7-oxo-pimara-8,15-diene-18-ol (**2**): colorless gum, ${\left[\alpha \right]}_{D}^{24}$: +12.4 (CHCl${}_{3}$); UV$\lambda_{max}$: 220 and 254 nm. +ESI MS (*m/z*): 163.1145, 255.2149 [M + H − HCOH − H${}_{2}$O]${}^{+}$, 273.2222 [M + H − HCOH]${}^{+}$, 285.2222 [M + H − H${}_{2}$O]${}^{+}$, 303.2304 [M + H]${}^{+}$, 325.2153 [M + Na]${}^{+}$; ${}^{1}$H and ${}^{13}$C-NMR (CDCl${}_{3}$), see Table 2. 

*ent*-7-oxo-pimara-8,15-diene-3*β*-ol (**3**): white amorphous solid; ${\left[\alpha \right]}_{D}^{24}$: −49.2 (CDCl${}_{3}$); UV (H${}_{2}$O-MeCN) $\lambda_{max}$: 220 and 250 nm; +ESI MS (*m/z*): 303.2369 [M + H]${}^{+}$, 325.2156 [M + Na]${}^{+}$, 605.4628 [2M + Na]${}^{+}$; ${}^{1}$H and ${}^{13}$C-NMR (CDCl${}_{3}$), see Table 2. 

*ent*-2*S*,4*S*-2-19-epoxy-pimara-8(3),15-diene-7*β*-ol (**4**): white amorphous solid; ${\left[\alpha \right]}_{D}^{25}$: −24.7 (CHCl${}_{3}$); UV (H${}_{2}$O-MeCN) $\lambda_{max}$: 220 nm; +ESI MS (*m/z*): 227.1842, 267.2159 [M + H − 2(H${}_{2}$O)]${}^{+}$, 285.2275 [M + H − H${}_{2}$O]${}^{+}$, 303.2369 [M + H]${}^{+}$, 605.4666 [2M + H]${}^{+}$; ${}^{1}$H and ${}^{13}$C-NMR (CDCl${}_{3}$), see Table 2. 

budlein A (**5**): white amorphous solid; UV (H${}_{2}$O-MeCN) $\lambda_{max}$: 220 and 275 nm; +ESI MS (*m/z*): 275.0918 [M + H-acetate)]${}^{+}$, 375.1446 [M + H]${}^{+}$, 397.1253 [M + Na]${}^{+}$, 749.2790 [2M + H]${}^{+}$, 771.2596 [2M + Na]${}^{+}$.

### 6.3. In Vitro Assays for the Antiprotozoal Activity of the Crude Extract and Pure Compounds

In vitro tests for the activity against *Trypanosoma brucei rhodesiense* (bloodstream trypomastigotes, STIB 900 strain), *T. cruzi* (amastigotes, Tulahuen C4 strain), *Leishmania donovani* (amastigotes, MHOM-ET-67/L82 strain), *Plasmodium falciparum* (intraerythrocytic forms, NF54 IEF strain), as well as for cytotoxicity against mammalian cells (L6-cell-line from rat-skeletal myoblasts) were performed according to established protocols as reported previously, e.g, in [33].

## 7. Conclusions

The investigation of the dichloromethane extract of *A. discolor* leaves led to the isolation of four structurally and biosynthetically related diterpenes, namely three new *ent*-pimar-15-ene derivatives (**2**, **3** and **4**) as well as a known ent-kaurane (**1**). Furthermore, the known sesquiterpene lactone budlein A (**5**) was identified as a major compound. The strong growth inhibition of *A. discolor* extracts towards *T. b. rhodesiense* can be explained by the presence of budlein A as a major compound, since this STL reportedly possesses strong anti-*T. b. rhodesiense* activity [26]. Among the isolated diterpenes, *ent*-pimarane **2** showed considerable in vitro activity against *P. falciparum*, while *ent*-kaurane **1** presented the highest in vitro activity against both *P. falciparum* and *L. donovani*. Quite importantly, they returned significant SI >10, which makes them interesting candidates for in vivo tests. In order to better understand their mechanism of action, further studies will be necessary.

## Figures and Tables

**Table 1:** In vitro growth inhibition (%) of *Aldama discolor* crude extracts against *T. b. rhodesiense*, *T. cruzi*, *L. donovani* and *P. falciparum*. DCM, dichloromethane.

Extract	*T. b. rhodesiense*		*T. cruzi*		*L. donovani*		*P. falciparum*		Yield (%) ${}^{a}$
	2 µg/mL	10 µg/mL	2 µg/mL	10 µg/mL	2 µg/mL	10 µg/mL	2 µg/mL	10 µg/mL	
Hex	31.2	100.0	0.0	6.8	25.0	86.9	-	99.8	3.0
DCM	100.0	100.0	0.0	0.0	21.1	86.4	5.7	99.7	28.0
EtOAc	100.0	99.8	6.9	14.8	14.2	87.8	17.8	100.0	25.2
MeOH	100.0	100.0	0.0	5.4	34.1	69.0	45.2	87.4	9.4

${}^{a}$ Percentage of dried crude extract in relation to the pulverized dried plant material. All extracts were prepared with 500 mg of the plant material.

**Table 2:** ${}^{13}$C-NMR and ${}^{1}$H-NMR spectroscopic data for **1**–**4** (150 and 600 MHz, CDCl${}_{3}$).

	1 ${}^{a}$			2			3			4		
	*δ* ${}^{13}$C	*δ* ${}^{1}$H	*J* (Hz)	*δ* ${}^{13}$C	*δ* ${}^{1}$H	*J* (Hz)	*δ* ${}^{13}$C	*δ* ${}^{1}$H	*J* (Hz)	*δ* ${}^{13}$C	*δ* ${}^{1}$H	*J* (Hz)
1	38.6	*β* 0.89 ddd		35.5	a 1.84 ddd	16.6, 12.8, 3.8	34.3	*α* 1.88 dt	13, 3.5	36.8	*α* 1.79 m	13, 11, 3.6
		*α* 1.87 ddd			b 1.28 ddd	16.6, 12.8, 4.1		*β* 1.42 ddd	13, 4.2, 3.5		*β* 1.21 m	13, 6, 2.7
2	27.9	*β* 1.84 ddd	13.4, 11.8, 3.6	18.3	a 1.65 dt	13.8, 3.8	27.4	*α* 1.69 ddd	13.3, 11.7, 3.5	81	*α* 3.53 ddd	11.5, 4.6, 1.3
		*α* 1.71 ddd	13.4, 8, 4.4		b 1.71 dt	13.8, 3.5		*β* 1.77 dq	13.3, 3.9			
3	81.1	3.42 ddd	11.8, 4.4, 1.3	34.6	a 1.33 ddt	12.8, 3.8, 1.3	78.1	3.29 dd	11.7, 4.4	28.1	a 1.74 m	
					b 1.52 ddd	13.8, 12.8, 4.2					b 1.78 ddd	13.2, 3.6, 1.8
4	43.1			37.7			38.9			42.4		
5	55.9	0.87 ddd	11.8, 6, 1.7	43.2	2.08 dd	13.4, 4.5	49.1	1.69 dd	11.7, 3.9	47.5	1.71 m	
6	20.2	*β* 1.30 ddd	16, 11.8, 3.8	35.3	*α* 2.37 m		35.1	*α* 2.42 dd	17.7, 11.7	28.9	*α* 1.55 m	
		*α* 1.64 m			*β* 2.42 m			*β* 2.51 dd	17.7,3.9		*β* 1.88 dt	13.9, 3.8
7	41.5	*β* 1.47 m		199.8			199.8			72.8	*α* 4.25 dd	6.8, 3.8
			*α* 1.51 m				175.2			176.2		
8	44.1			129.1			129.5			140.1		
9	56	1.03 d	6.9	165.8			164.7			46.7	2.05 ddd	10.2, 6.4, 2.1
10	38.9			39.5			39.5			38.1		
11	18.5	*β* 1.62 m		22.8	*α* 2.20 m		22.8	*α* 2.19 m		18.9	*α* 1.54 m	
		*α* 1.52 ddd	13.4, 6.9, 3.6		*β* 2.31 m			*β* 2.27 m			*β* 1.30 m	
12	39.7	*β* 1.91 d	11.5	33.1	*α* 1.47 m		33	*α* 1.43 m		35.4	*α* 1.57 m	
		*α* 1.09 ddt	11.5, 5, 1.8		*β* 1.44 m			*β* 1.46 m			*β* 1.23 m	
13	44	2.64 t	5	34.3			34.2			38.8		
14	33.2	pax 1.47 m		33.6	*β* 1.99 dq	17, 2.3	33.6	*α* 2.30 dt	17, 2	133.9	5.47 t	1.7
		peq 1.63 m			*α* 2.31 dq	17, 2.3		*β* 1.99 dq	17, 1.4			
15	49	2.05 t	2.5	147.4	5.75 dd	17.7, 10.6	147.2	5.74 dd	17.2, 10.7	146.5	5.68 dd	17.3, 10.4
16	155.7			110.8	4.89 dd	17.7, 1	110.9	4.88 dd	17.2, 1.4	113.5	4.82 dd	17.3, 1.7
					4.91 dd	10.6, 1		4.9	10.7, 1.4		4.97 dd	10.4, 1.7
17	103.3	4.73 ddt	2.5, 1.8, 1.3	24.8	0.95 s		24.8	0.94 s		29.2	1.02 s	
		4.79 m					119			119		
18	22.8	1.22 s		70.9	a 3.13 d	10.9	27.5	1.0 s		22.4	1.27 s	
					b 3.41 d	10.9						
19	64.5	a 4.20 d	11.2	17.5	0.87 s		15.3	0.89 s		64.4	a 4.25 d	11.1
		b 3.31 dd	11.2, 1.3								b 3.34 dd	11.1, 1.5
20	18.3		0.98 s	18.8	1.10 s		18.3	1.06 s		15.2	0.65 s	

${}^{a}$ Assignments of the proton shifts were compared with those described by Lloyd et al. [29] and Dutra et al. [30].

**Table 3:** IC${}_{50}$ (geometric mean of 2 experiments ±SE) of the isolated compounds (μM) tested against *T. b. rhodesiense*, *T. cruzi*, *L. donovani*, *P. falciparum* and L6 cells. The selectivity index (SI) determined with L6 cells is also shown.

Comp.	*T. b. rhodesiense*	SI	*T. cruzi*	SI	*L. donovani*	SI	*P. falciparum*	SI	L6 Cells
**1**	20.1 ± 0.5	2	55.6 ${}^{a}$	1	2.5 ± 1.5	16	3.5 ± 0.2	11	40.2 ± 5.5
**2**	24.3 ${}^{a}$	2	15.4 ${}^{a}$	3	18.2 ${}^{a}$	3	3.8 ${}^{a}$	13	48.3 ± 5.9
**3**	NA		19.4 ${}^{a}$	4	13.8 ${}^{a}$	5	16.5 ± 4.9	4	69.3 ± 10.9
**4**	47.3 ± 4.1	2	58.9 ${}^{a}$	2	21.9 ${}^{a}$	5	16.1 ± 6.1	6	101.0 ± 45.7
**Pos. controls**	0.025 ± 0.0005		0.653 ± 0.135		0.049 ± 0.001		0.003 ± 0.001		0.008 + 0.001

*T. b. rhodesiense* (STIB 900 strain, trypomastigotes, pos.control: melarsoprol); *T. cruzi* (Tulahuen C4 strain, amastigotes, pos. control: benznidazole); *L. donovani* (MHOM-ET-67/L82 strain, amastigotes, pos. control: miltefosine); *P. falciparum* (NF54 strain, intra-erythrocytic stages, pos. control: chloroquine), cytotoxicity (L6-cell-line from rat-skeletal myoblasts, pos. control: podophyllotoxin); ${}^{a}$ only one experiment was carried out to obtain the IC${}_{50}$; NA: not assayed due to the indication of a lack of activity in a prediction study (unpublished data).

## Supplementary Materials

Supplementary materials can be accessed at: http://www.mdpi.com/1420-3049/21/9/1237/s1.

## References

[1] Schilling E.E., Panero J.L. (2011). A revised classificationcation of subtribe Helianthinae (Asteraceae: Heliantheae). I. Basal lineages. Bot. J. Linn. Soc..

[2] Blake S.F. (1918). A Revision of the Genus Viguiera.

[3] Meragelman K.M., Espinar L.A., Sosa V.E., Uriburu M.L., de la Fuente J.R. (1996). Terpenoid constituents of *Viguiera tucumanensis*. Phytochemistry.

[4] Da Costa F.B., Schorr K., Arakawa N.S., Schilling E.E., Spring O. (2001). Infraspecific variation in the chemistry of glandular trichomes of two Brazilian *Viguiera* species (Heliantheae; Asteraceae). J. Braz. Chem. Soc..

[5] Delgado G., Vivar A.R.D.E., Herz W., Blake V. (1982). Sesquiterpene lactones from *Viguiera* species. Phytochemistry.

[6] Delgado G., Cirdenas H., Pelez G., Vivar A.R.D., Pereda-Miranda R. (1984). Terpenoids from *Viguiera excelsa* and *Vigueira oaxacana*. J. Nat. Prod..

[7] Spring O., Zipper R., Klaiber I., Reeb S., Vogler B. (2000). Sesquiterpene lactones in *Viguiera eriophora* and *Viguiera puruana* (Heliantheae; Asteraceae). Phytochemistry.

[8] Gao F., Miski M., Gage D.A., Norris J.A., Mabry T.J. (1991). Terpenoids from *Viguiera potosina*. J. Nat. Prod..

[9] Gao F., Miski M., Gage D.A., Mabry T.J. (1985). Terpenoid constituents of *Viguiera dentata*. J. Nat. Prod..

[10] Da Costa F.B., Albuquerque S., Vichnewski W. (1996). Diterpenes and synthetic derivatives from *Viguiera aspillioides* with trypanomicidal activity. Planta Med..

[11] Delgado G., Vivar A.R.D.E., Ortega A., Cardenas J. (1983). Diterpenoids from *Viguiera insignis*. Phytochemistry.

[12] Delgado G., De Vivar A.E., Cardenas J., Pereda-Miranda R., Huerta E. (1984). *ent*-Beyerene and ent-atisene diterpenes from *Viguiera insignis*. Phytochemistry.

[13] Zamilpa A., Tortoriello J., Navarro V., Delgado G., Alvarez L. (2002). Antispasmodic and antimicrobial diterpenic acids from *Viguiera hypargyrea* roots. Planta Med..

[14] Bohlmann F., Zdero C., Schmeda-Hirschmann G., Jakupovic J., Castro V., King R.M., Robinson H. (1984). Heliangolide, trachyloban- und villanovan-derivate aus *Viguiera* arten. Liebigs Annal. Chem..

[15] Guerrero C., Nava A.L., Quevedo F., Toscano R.A., Soriano-Garcia M. (1986). Further constituents of *Viguiera stenoloba* and *Viguiera pinnatilobata*. Rev. Latinoam. Quim..

[16] Ambrosio S.R., Schorr K., da Costa F.B. (2004). Terpenoids of *Viguiera arenaria* (Asteraceae). Biochem. Syst. Ecol..

[17] Ambrosio S.R., Arakawa N.S., Esperandim V.R., de Albuquerque S., da Costa F.B. (2008). Trypanocidal activity of pimarane diterpenes from *Viguiera arenaria* (Asteraceae). Phytother. Res..

[18] Villarreal M.L., Alvarezb L., Alonsoa D., Navarroa V., Garciab P., Delgadoc G. (1994). Cytotoxic and antimicrobial screening of selected terpenoids from Asteraceae species. J. Ethnopharmacol..

[19] Carvalho T.C., Furtado N.A.J.C., Veneziani R.C.S., Heleno V.C.G., Souza M.G.M., Martins C.H.G., Franca U.D., Campinas D. (2011). Antimicrobial activity of diterpenes from *Viguiera arenaria* against Endodontic Bacteria. Molecules.

[20] Souza T., Furtado N.A.J.C., Heleno V.C.G., Martins C.H.G., Da F.B., Severiano M.E., Silva A.N., Veneziani R.C.S., Ambrósio S.R. (2009). Antimicrobial *ent*-pimarane diterpenes from *Viguiera arenaria* against gram-positive bacteria. Fitoterapia.

[21] Chagas-Paula D.A., Oliveira T.B., Faleiro D.P.V., Oliveira R.B., Costa F.B.D. (2015). Outstanding anti-inflammatory potential of selected Asteraceae species through the potent dual inhibition of Cyclooxygenase-1 and 5-Lipoxygenase. Planta Med..

[22] Marquina S., Maldonado N., Gardun M.L., Aranda E., Villarreal M.L., Navarro V., Bye R., Delgado G., Alvarez L. (2001). Bioactive oleanolic acid saponins and other constituents from the roots of *Viguiera decurrens*. Phytochemistry.

[23] Guillet G., Chauret D., Arnason J.T. (1997). Phototoxic polyacetylenes from *Viguiera annua* and adaptations of a Chrysomelide Beetle, *Zygogramma continua*, feeding on this plant. Phytochemistry.

[24] Ambrosio S.R., Tirapelli C.R., Fernando B., Oliveira A.M.D. (2006). Kaurane and pimarane-type diterpenes from the *Viguiera* species inhibit vascular smooth muscle contractility. Life Sci..

[25] Schmidt T.J., Khalid S.A., Romanha A.J., Alves T.M., Biavatti M.W., Brun R., da Costa F.B., de Castro S.L., Ferreira V.F., de Lacerda M.V. (2012). The potential of secondary metabolites from plants as drugs or leads against protozoan neglected diseases—Part I. Curr. Med. Chem..

[26] Schmidt T.J., da Costa F.B., Lopes N.P., Kaiser M., Brun R. (2014). Silico prediction and experimental evaluation of furanoheliangolide sesquiterpene lactones as potent agents against *Trypanosoma brucei rhodesiense*. Antimicrob. Agents Chemother..

[27] Nogueira M.S. (2016). The Use of Chemometric and Chemoinformatic Tools for Identification and Targeted Isolation of Compounds from Asteraceae with Antiprotozoal Activity. Ph.D. Thesis.

[28] Nogueira M.S., da Costa F.B., Magenta M.A., Kaiser M., Brun R., Schmidt T.J. (2013). Screening of some Asteraceae to discover new active compounds against *Trypanosoma brucei* by metabolite profiling and PLS analysis. Planta Med..

[29] Lloyd H.A., Fallis A. (1967). Terpene alcohols of *Helichrysum dendroideum*. Tetr. Lett..

[30] Dutra L.M., Bomfim L.M., Rocha S.L.A., Nepel A., Soares M.B.P., Barison A., Costa E.V., Bezerra D.P. (2014). *ent*-Kaurane diterpenes from the stem bark of *Annona vepretorum* (Annonaceae) and cytotoxic evaluation. Bioorgan. Med. Chem. Lett..

[31] Seca A.M.L., Pinto D.C.G.A., Silva A.M.S. (2008). Structural elucidation of pimarane and isopimarane diterpenoids: The ^13^C-NMR contribution. Nat. Prod. Commun..

[32] Lightner D.A., Gurst E.J. (2000). Organic Analysis and Stereochemistry from Circular Dichroism Spectroscopy.

[33] Schmidt T.J., Nour A.M.M., Khalid S.A., Kaiser M., Brun R. (2009). Quantitative structure—Antiprotozoal activity relationships of sesquiterpene lactones. Molecules.

[34] Asili J., Lambert M., Ziegler H.L., Stærk D., Sairafianpour M., Witt M., Asghari G., Ibrahimi I.S., Jaroszewski J.W. (2004). Erythrocyte membrane and on *Plasmodium falciparum* growth in the erythrocyte host cells. J. Nat. Prod..

[35] Thongnest S., Mahidol C., Sutthivaiyakit S., Ruchirawat S. (2005). Oxygenated pimarane diterpenes from *Kaempferia marginata*. J. Nat. Prod..

[36] Ziegler H.L., Franzyk H., Sairafianpour M., Tabatabai M., Tehrani M.D., Bagherzadeh K., Hägerstrand H., Stærk D., Jaroszewski J.W. (2004). Erythrocyte membrane modifying agents and the inhibition of *Plasmodium falciparum* growth: Structure-activity relationships for betulinic acid analogues. Bioorgan. Med. Chem..

[37] Ziegler H.L., Hansen H.S., Staerk D., Christensen S.B., Hägerstrand H., Jaroszewski J.W. (2004). The antiparasitic compound licochalcone A is a potent echinocytogenic agent that modifies the erythrocyte membrane in the concentration range where antiplasmodial activity is observed. Antimicrob. Agents Chemother..

[38] Otoguro K., Iwatsuki M., Ishiyama A., Namatame M., Nishihara-Tukashima A., Kiyohara H., Hashimoto T., Asakawa Y., Mura S., Yamada H. (2011). In vitro antitrypanosomal activity of plant terpenes against *Trypanosoma brucei*. Phytochemistry.

[39] Wagner H., Bladt S. (2001). Plant Drug Analysis: A Thin Layer Chromatography Atlas.

[40] Frisch M.J., Trucks G.W., Schlegel H.B., Scuseria G.E., Robb M.A., Cheeseman J.R., Montgomery J.A., Vreven T., Kudin K.N., Burant J.C. (2004). Gaussian 03, Revision C.02, 2004.

[41] Schmidt T., Vößing S., Klaes M., Grimme S. (2007). An aryldihydronaphthalene lignan with a novel type of ring system and further new lignans from *Linum perenne* L.. Planta Med..

[42] Barbic M., Schmidt T.J., Jügenliemk G. (2012). Novel phenyl-1-benzoxepinols from butcher’s broom (*Rusci rhizoma*). Chem. Biodivers..

