# Supplementary material for: ent-Pimarane and ent-Kaurane Diterpenes from Aldama discolor (Asteraceae) and Their Antiprotozoal Activity
Source: Molecules. 2016 Sep 15;21(9):1237. doi: 10.3390/molecules21091237 (PMC6274167; doi:10.3390/molecules21091237)
Supplement: Supplementary file 1 [file molecules-21-01237-s001.pdf]

Supplementary Materials: *ent*-Pimarane and *ent*-Kaurane Diterpenes from *Aldama discolor* (Asteraceae) and Their Antiprotozoal Activity

Mauro S. Nogueira, Fernando B. Da Costa, Reto Brun, Marcel Kaiser and Thomas J. Schmidt

1. Supporting Information

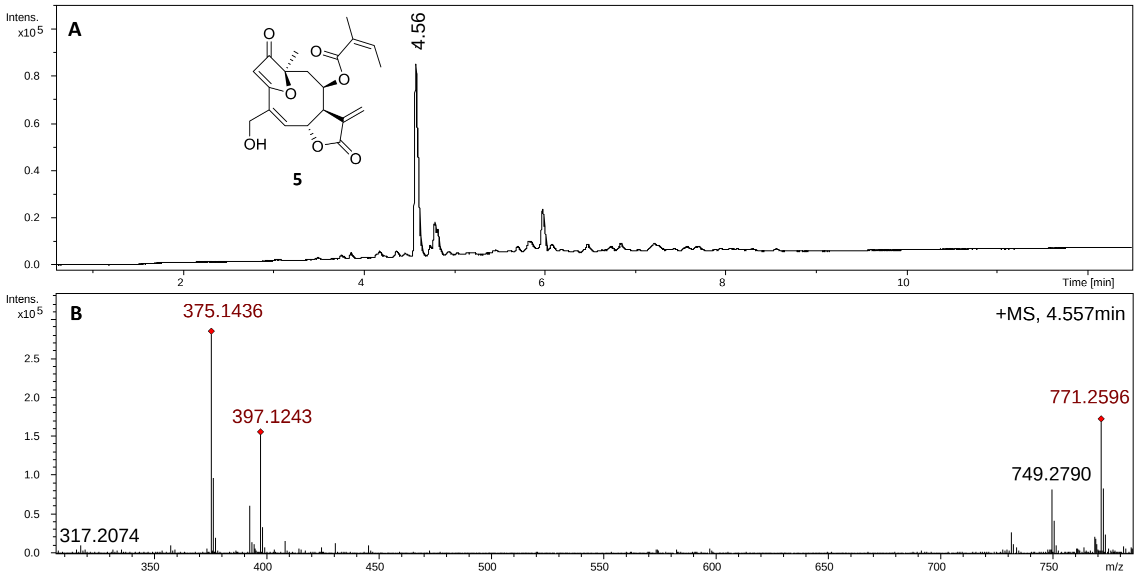

Figure S1. (**A**) LC-MS-chromatogram of the last fraction (elution with EtOAc) of the CC on silica gel of the dichloromethane extract of *A. discolor* leaves; (**B**) +ESI-MS of its major compound, budlein A. $[M + H]^+$: 375.1436 ($C_{20}H_{23}O_7$).

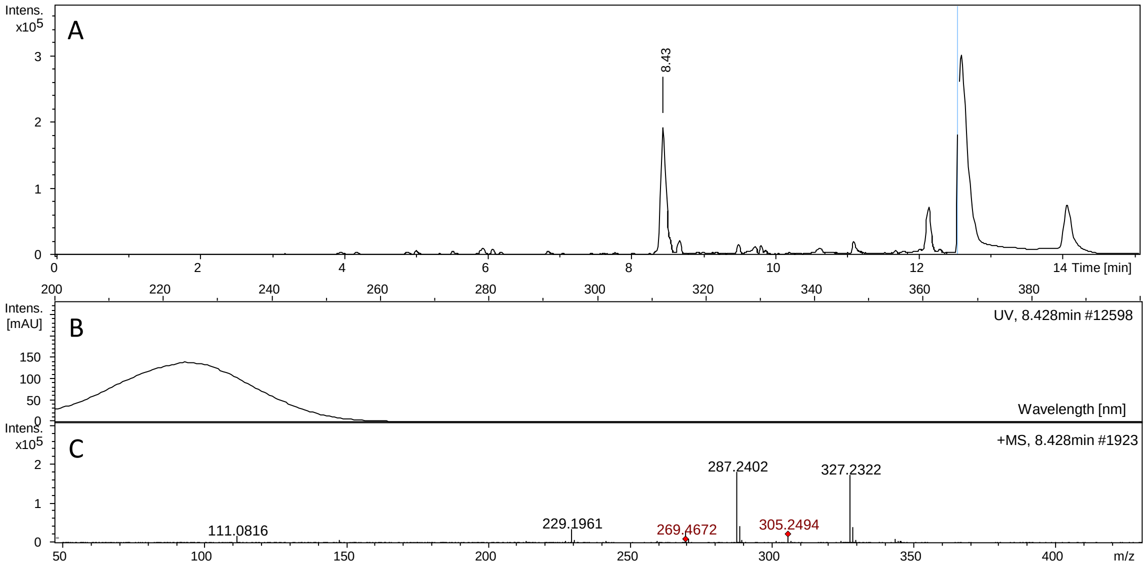

Figure S2. +ESI-QqTOF-MS chromatogram (**A**); UV spectrum (**B**); +ESI-MS (**C**) of compound **1**, $[M + H]^+$: 305.2494 ($C_{20}H_{33}O_2$) and $[M + Na]^+$: 327.2322 ($C_{20}H_{32}O_2Na$). Internal calibrant sodium formiate: 12.6 min.

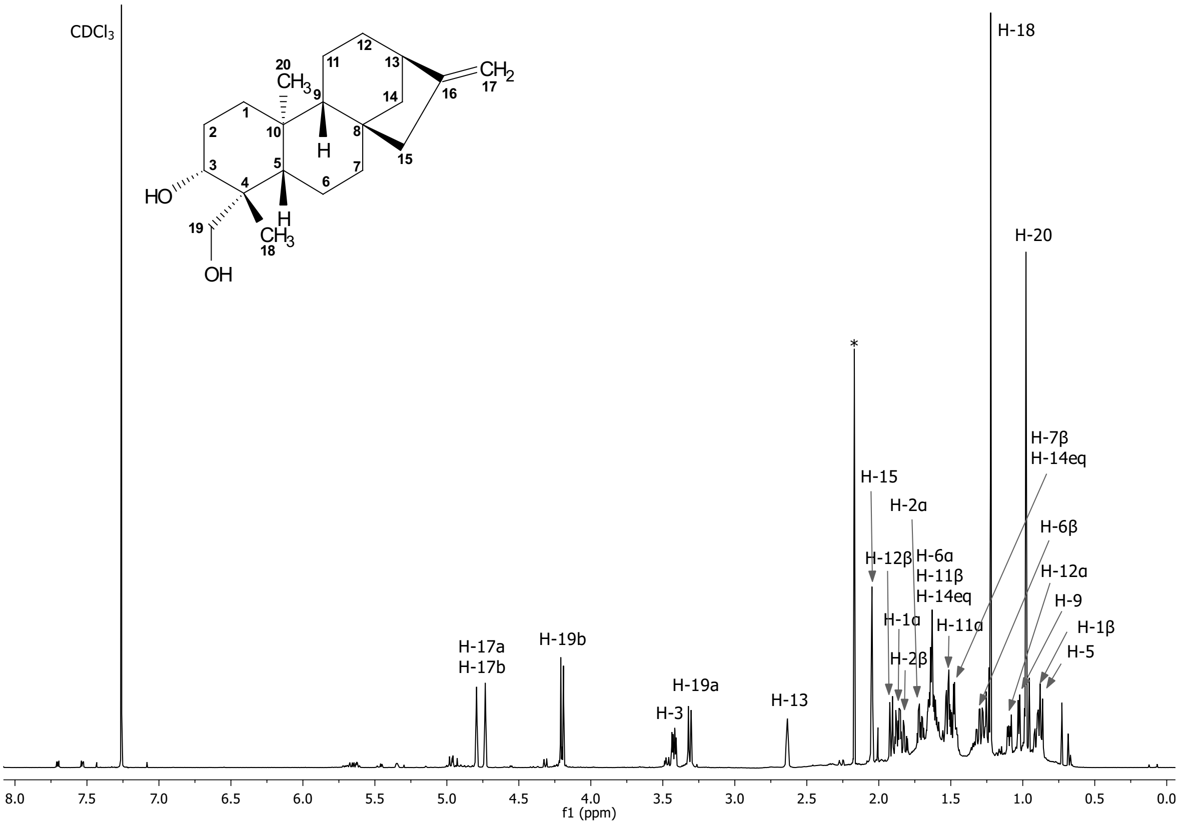

Figure S3. ^{1}H NMR spectrum of compound **1** ($CDCl_3$, 600 MHz). *-CH_3 protons from acetone.

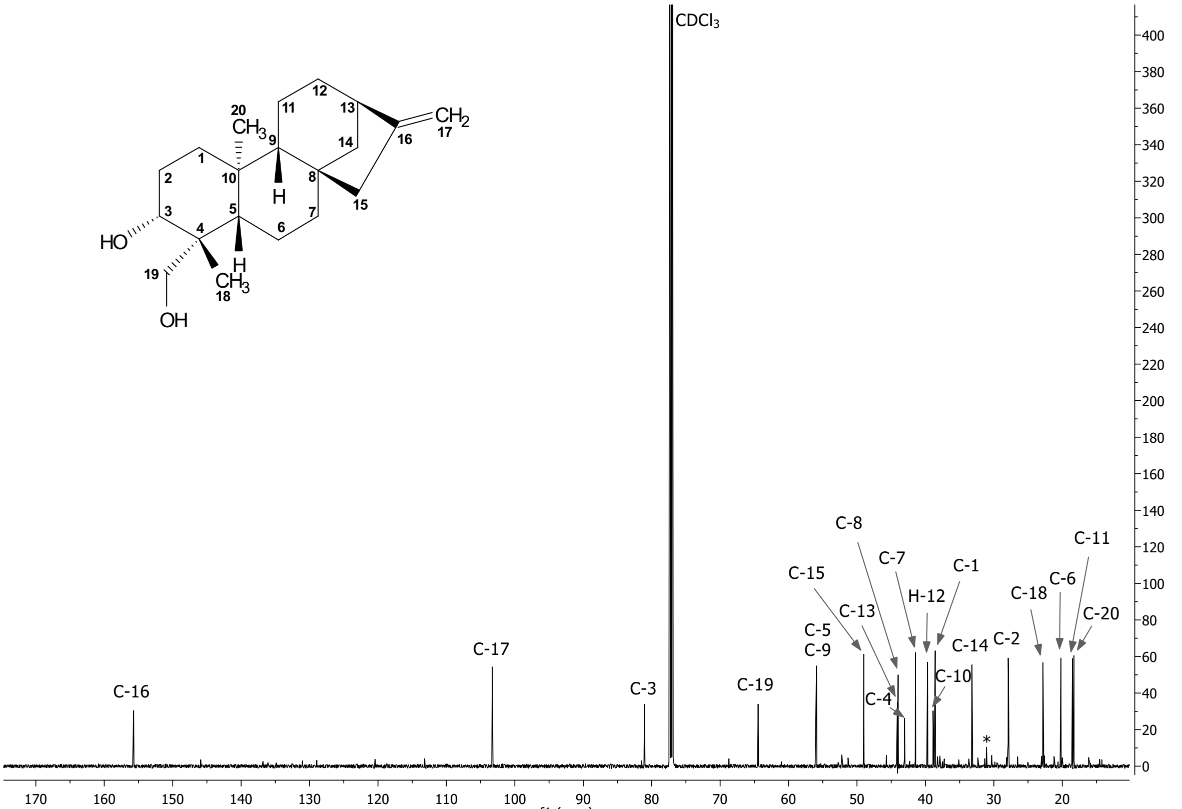

Figure S4. ^{13}C NMR spectrum of compound **1** ($CDCl_3$, 150 MHz). *-CH_3 from acetone.

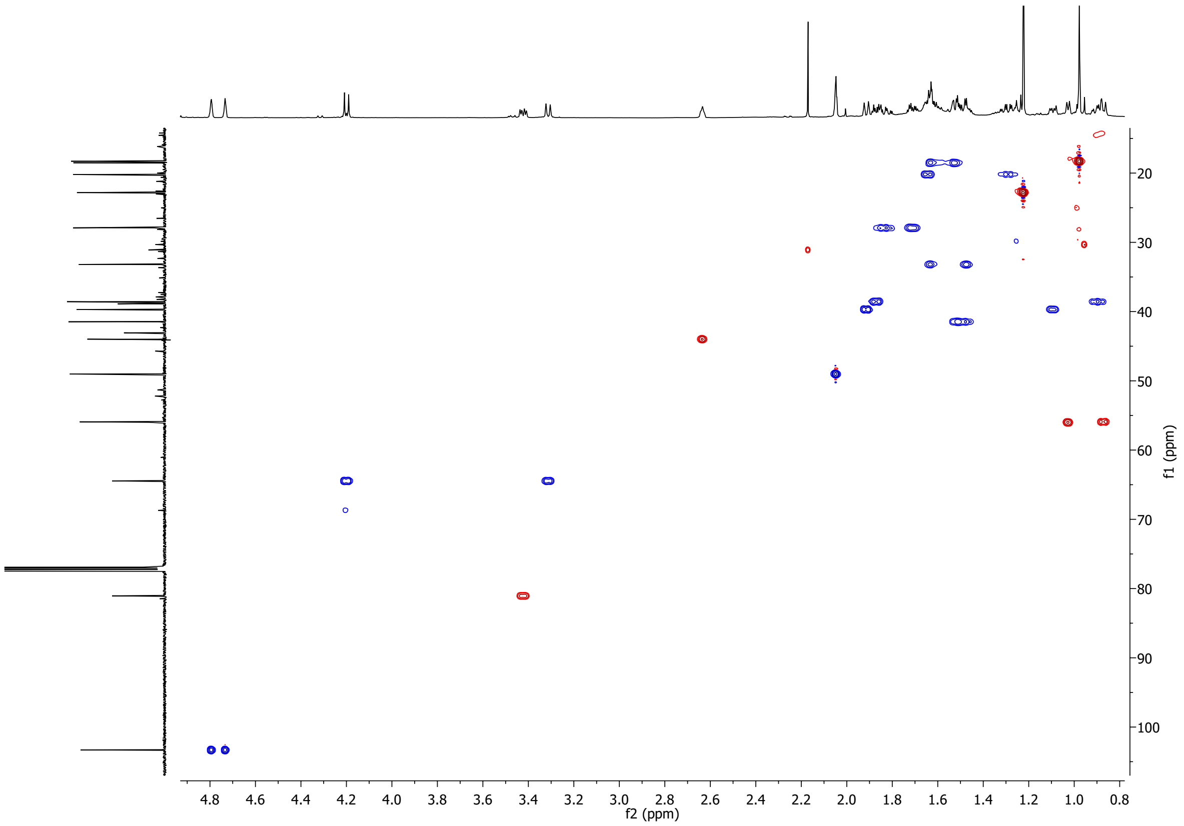

Figure S5. $^{1}H/^{13}$-HSQC spectrum of compound **1** (600/150 MHz, $CDCl_3$).

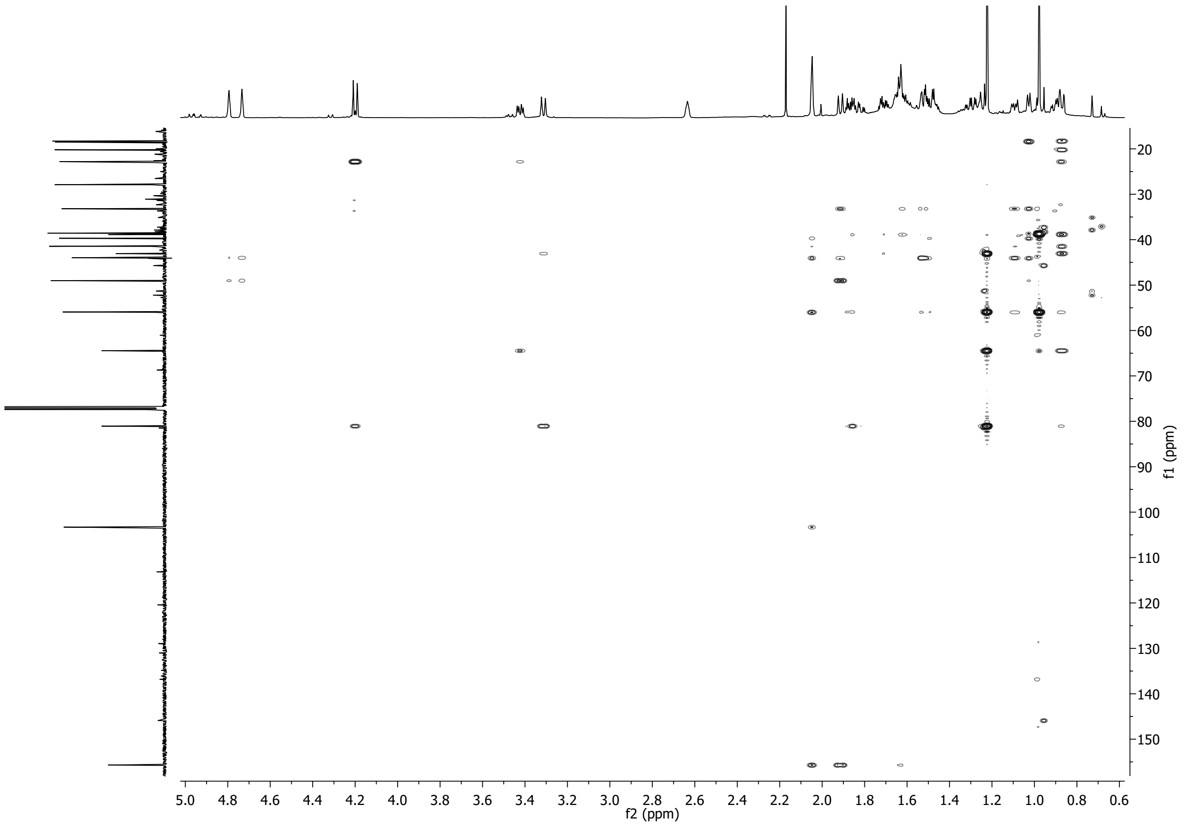

Figure S6. $^{1}H/^{13}$-HMBC spectrum of compound **1** (600/150 MHz, $CDCl_3$).

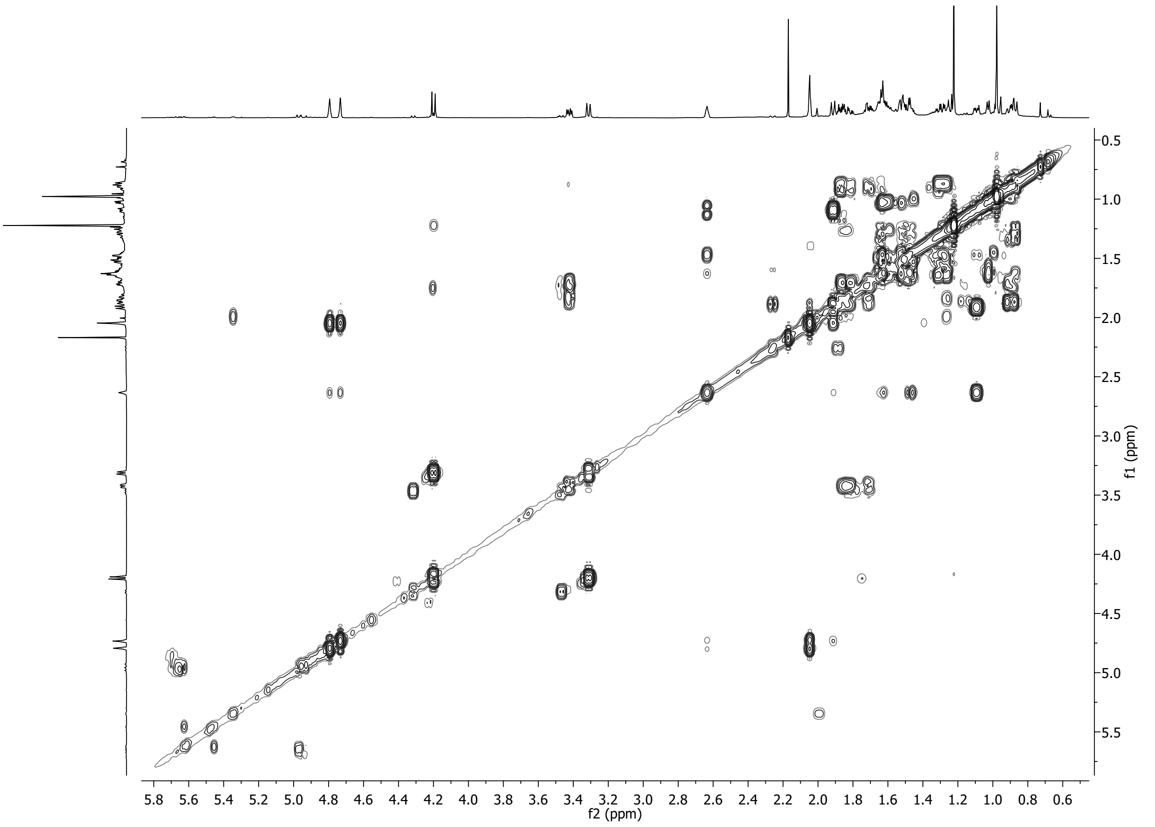

Figure S7. ^{1}H/^{1}H-COSY spectrum of compound **1** (600 MHz, $CDCl_3$).

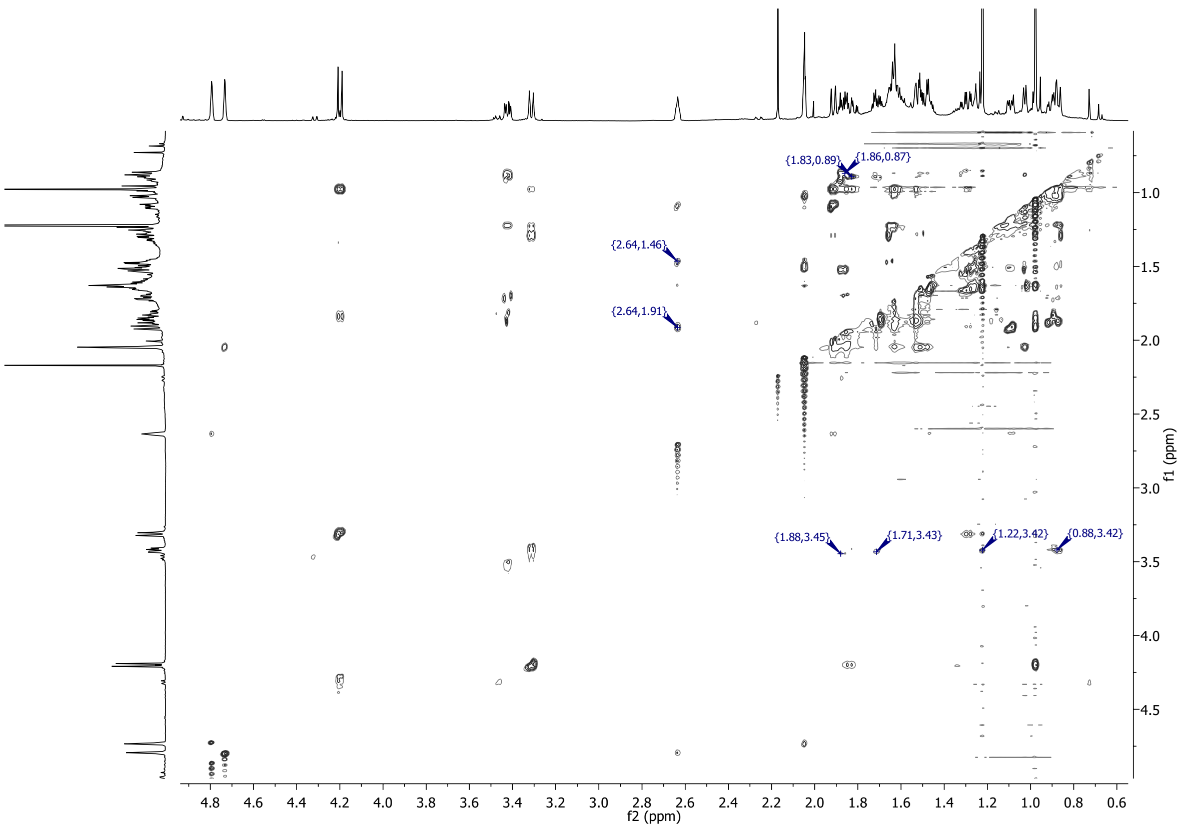

Figure S8. ^{1}H/^{1}H-NOESY spectrum of compound **1** (600 MHz, $CDCl_3$).

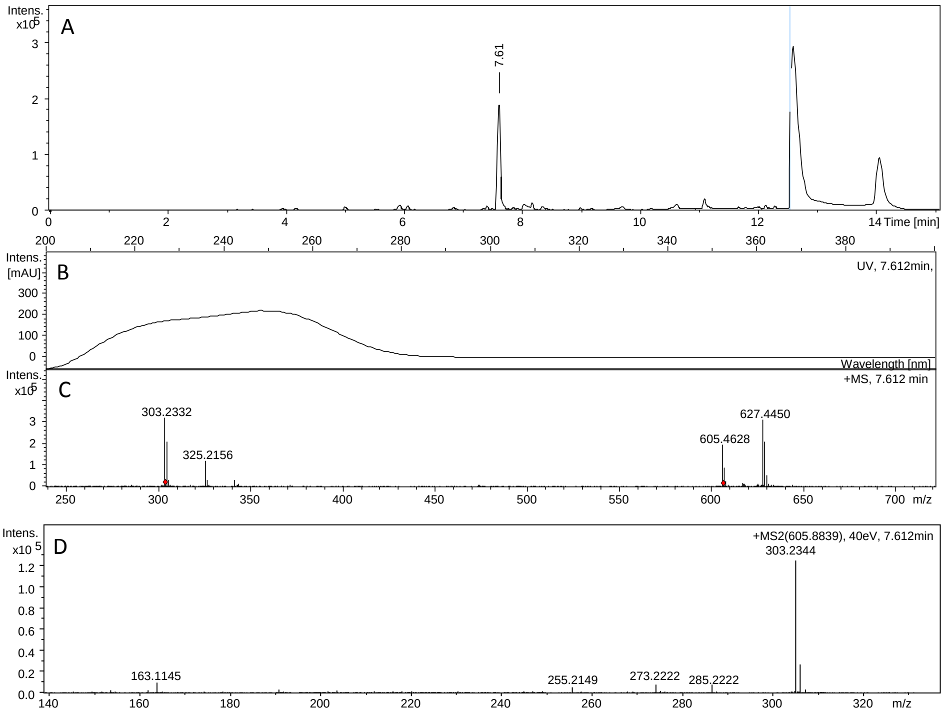

Figure S9. +ESI-QqTOF-MS chromatogram (**A**); UV spectrum (**B**); +ESI-MS (**C**) and +ESI-MS2 (**D**) spectra of compound **2**, $[M + H]^+$: 303.2346 ($C_{20}H_{31}O_2$). Internal calibrant sodium formiate: 12.6 min.

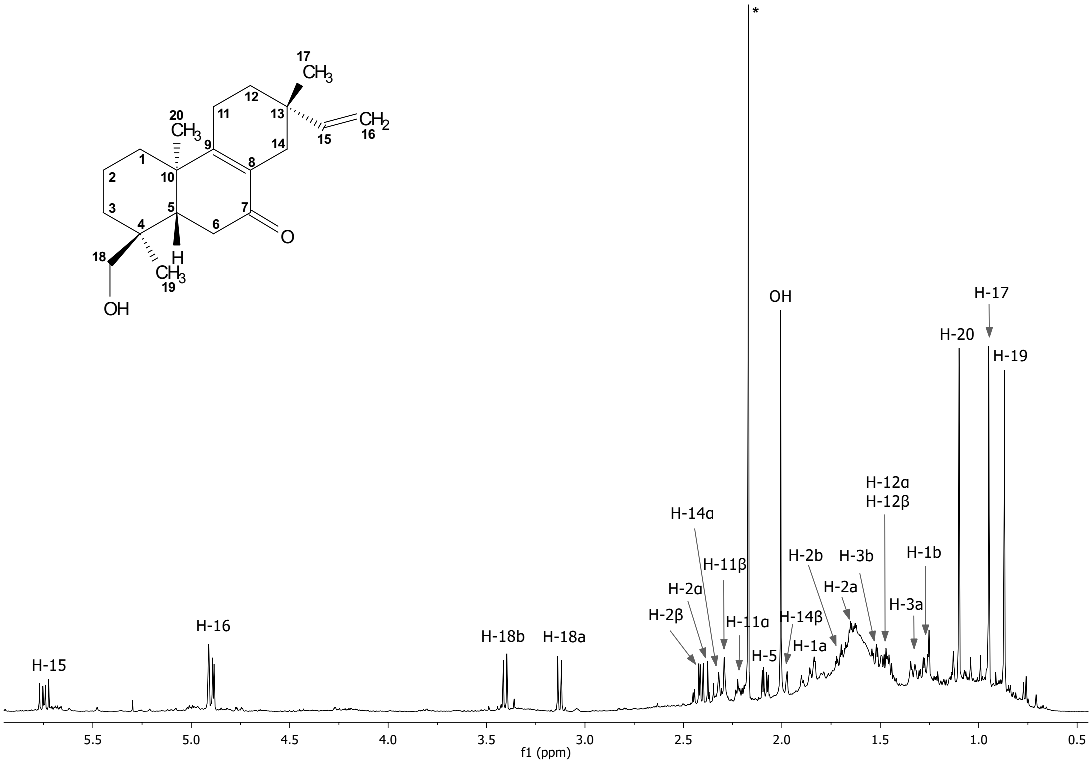

Figure S10. ^1H NMR spectrum of compound **2** ($CDCl_3$, 600 MHz). *-CH_3 protons from acetone.

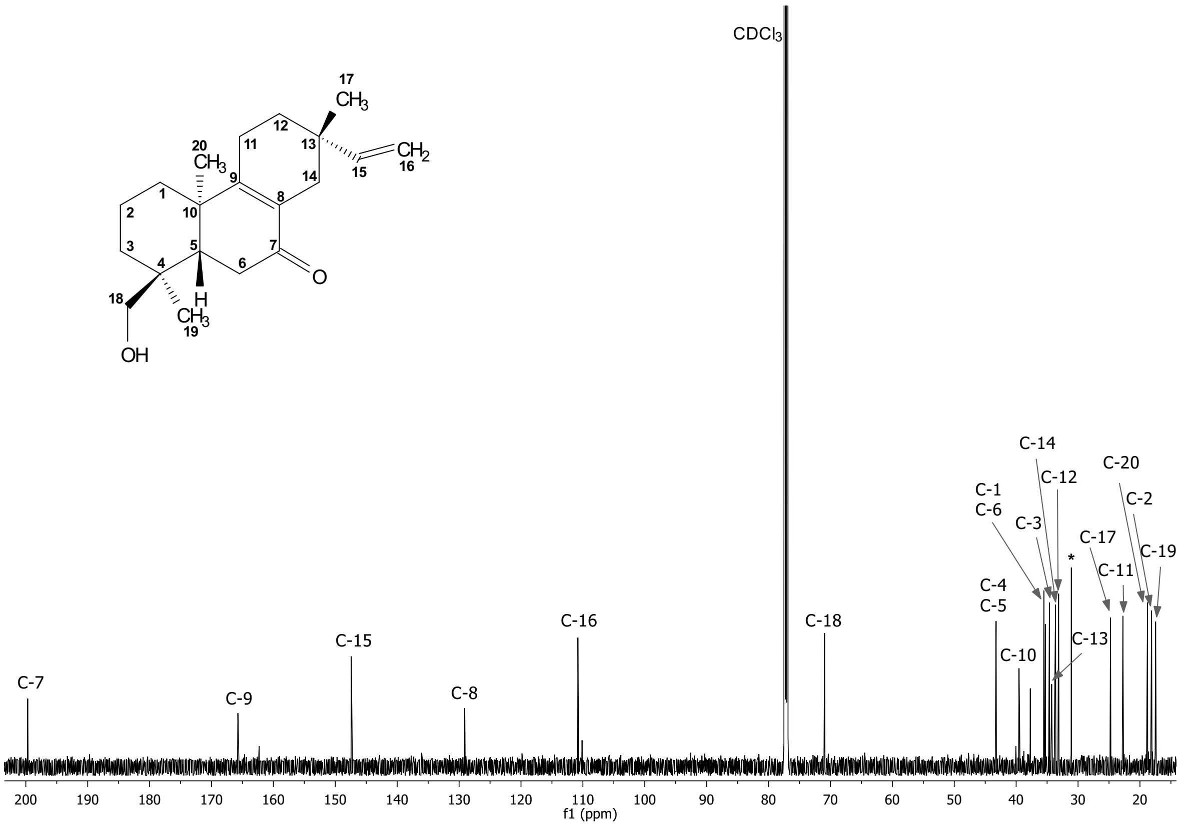

Figure S11. ^{13}C NMR spectrum of compound **2** ($CDCl_3$, 150 MHz). *-CH_3 from acetone.

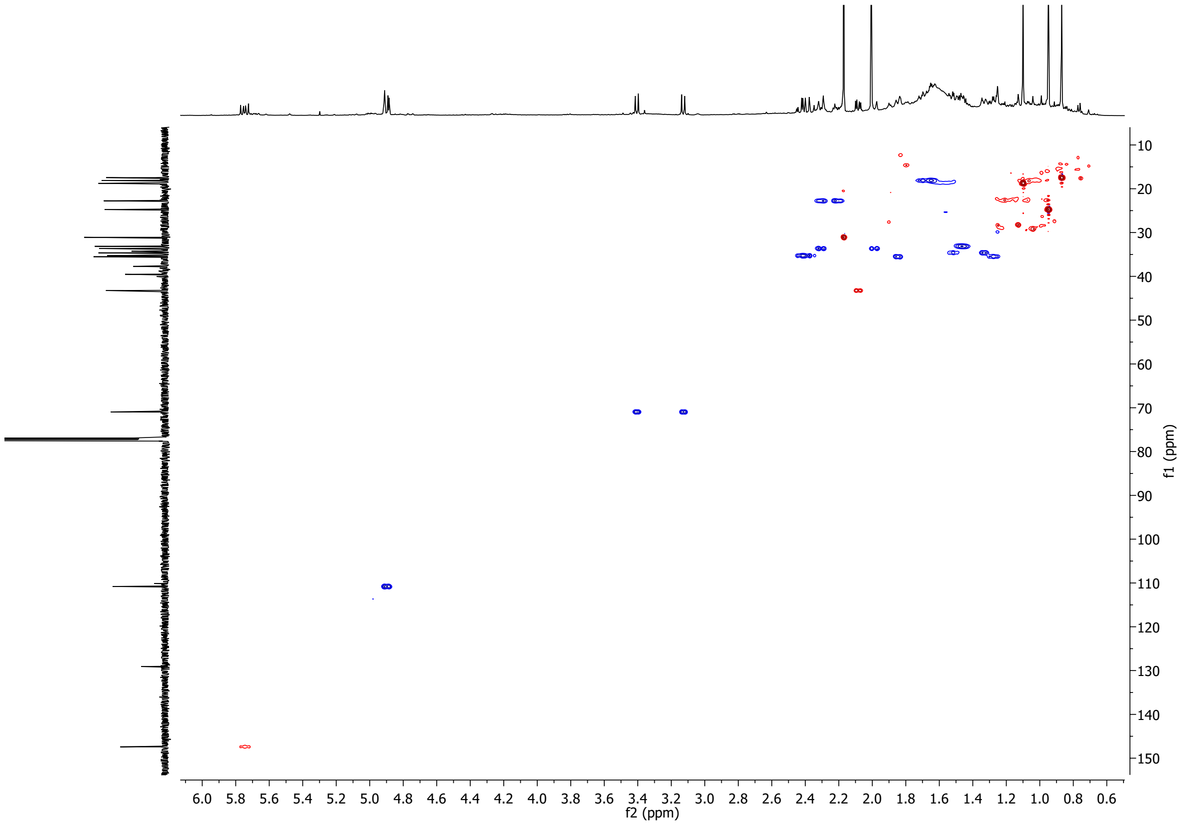

Figure S12. $^{1}H/^{13}$-HSQC spectrum of compound **2** (600/150 MHz, $CDCl_3$).

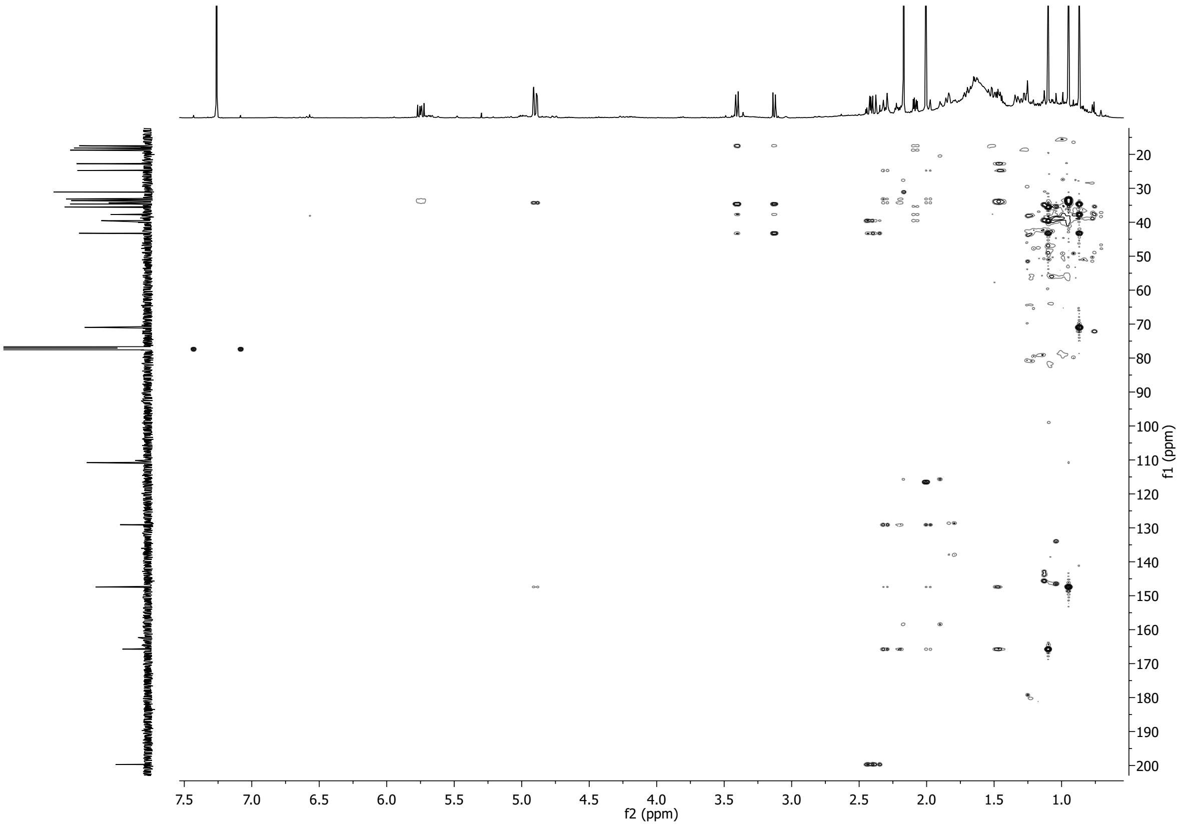

Figure S13. $^{1}H/^{13}$-HMBC spectrum of compound **2** (600/150 MHz, $CDCl_3$).

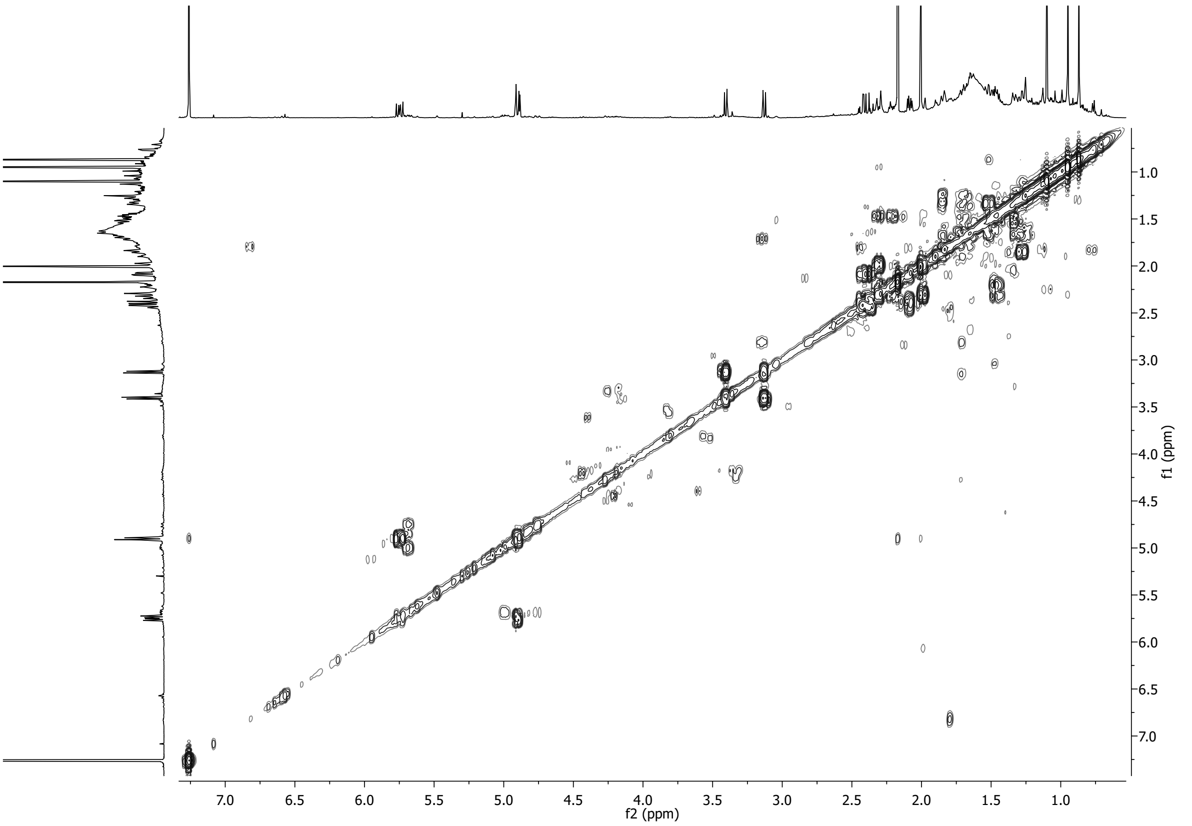

Figure S14. $^{1}H/^{1}H$-COSY spectrum of compound **2** (600 MHz, $CDCl_3$).

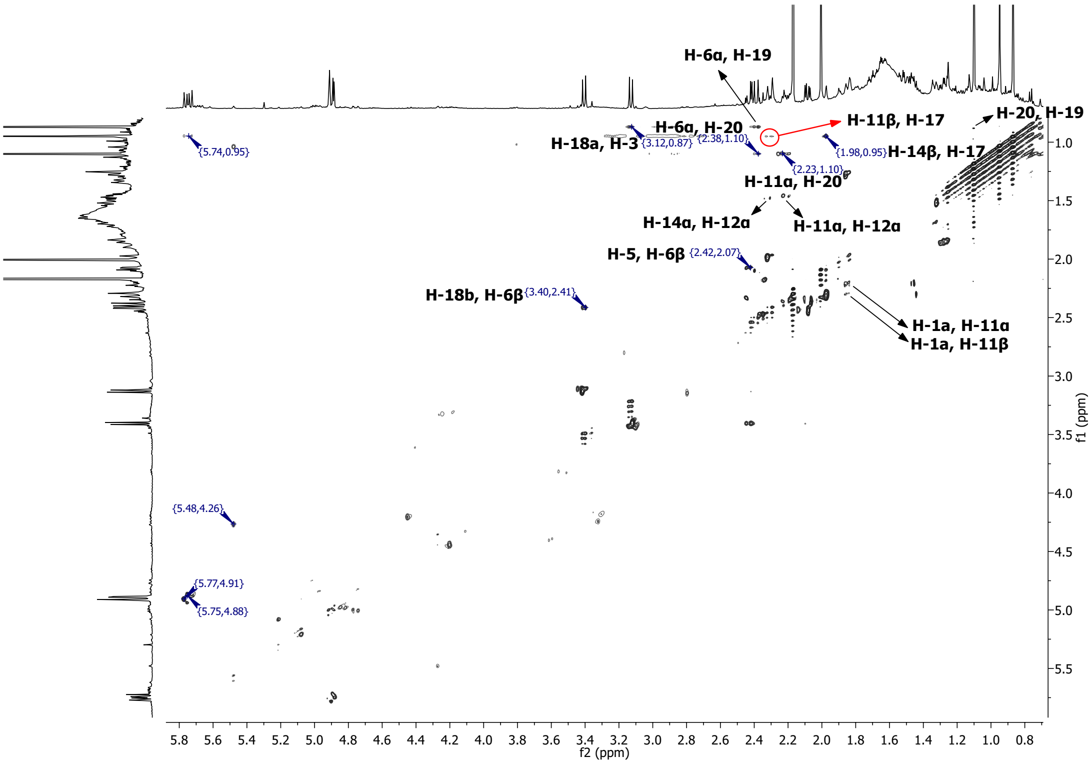

Figure S15. $^1H/^1H$-NOESY spectrum of compound **2** (600 MHz, $CDCl_3$). Cross-correlated peak (red circle) enabled the determination of the stereochemistry at C-13.

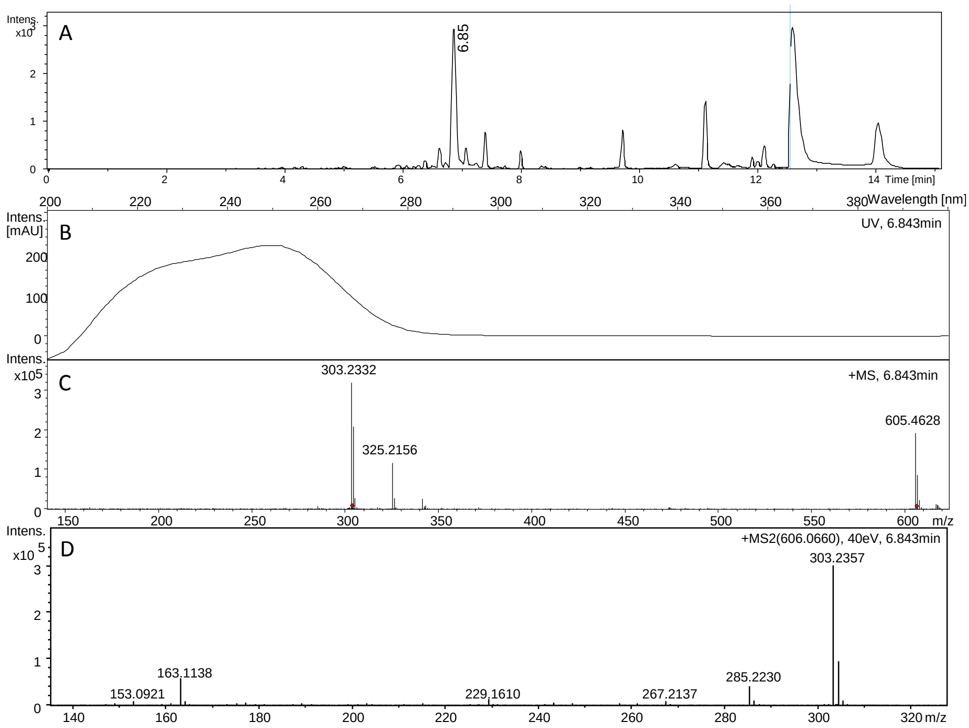

Figure S16. +ESI-QqTOF-MS chromatogram (**A**); UV spectrum (**B**); +ESI-MS (**C**) and +ESI-MS2 (**D**) spectra of compound **3**, $[M + H]^+$: 303.2332 ($C_{20}H_{31}O_2$). Internal calibrant sodium formiate: 12.6 min.

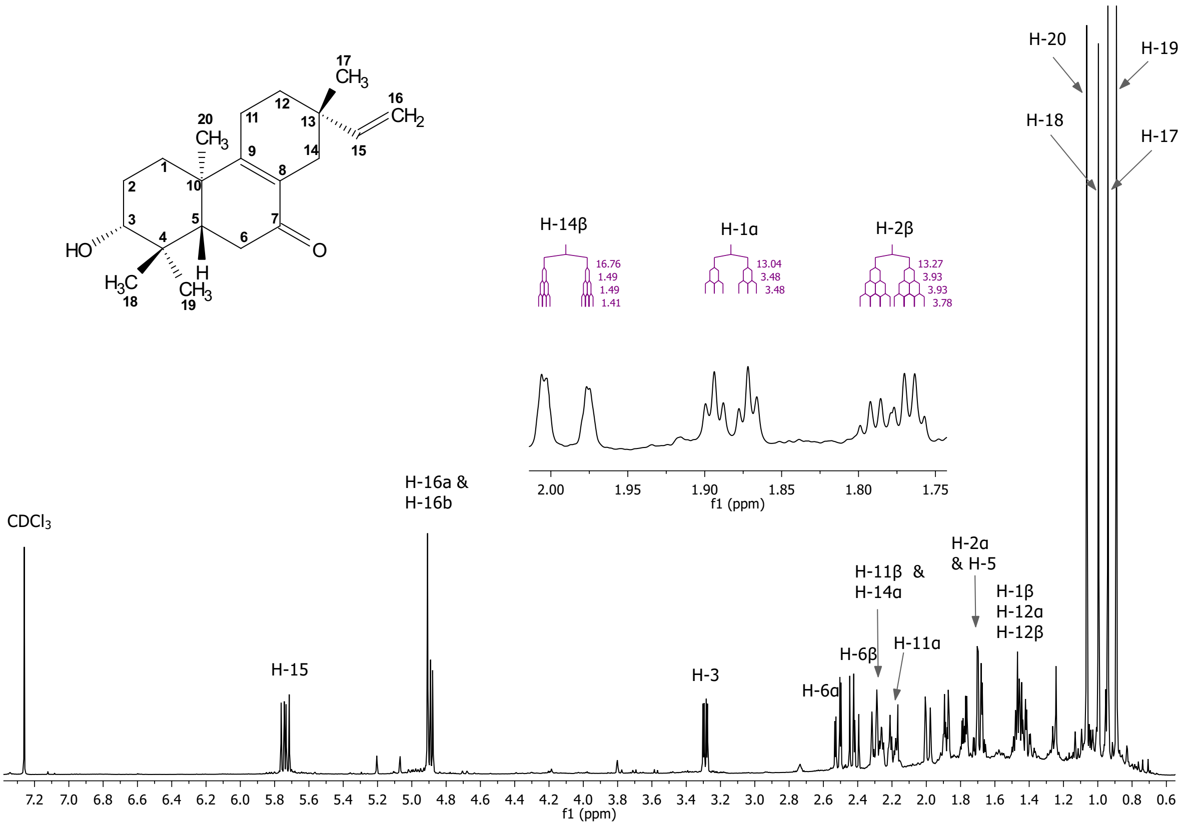

Figure S17. ^{1}H NMR spectrum of compound **3** ($CDCl_3$, 600 MHz).

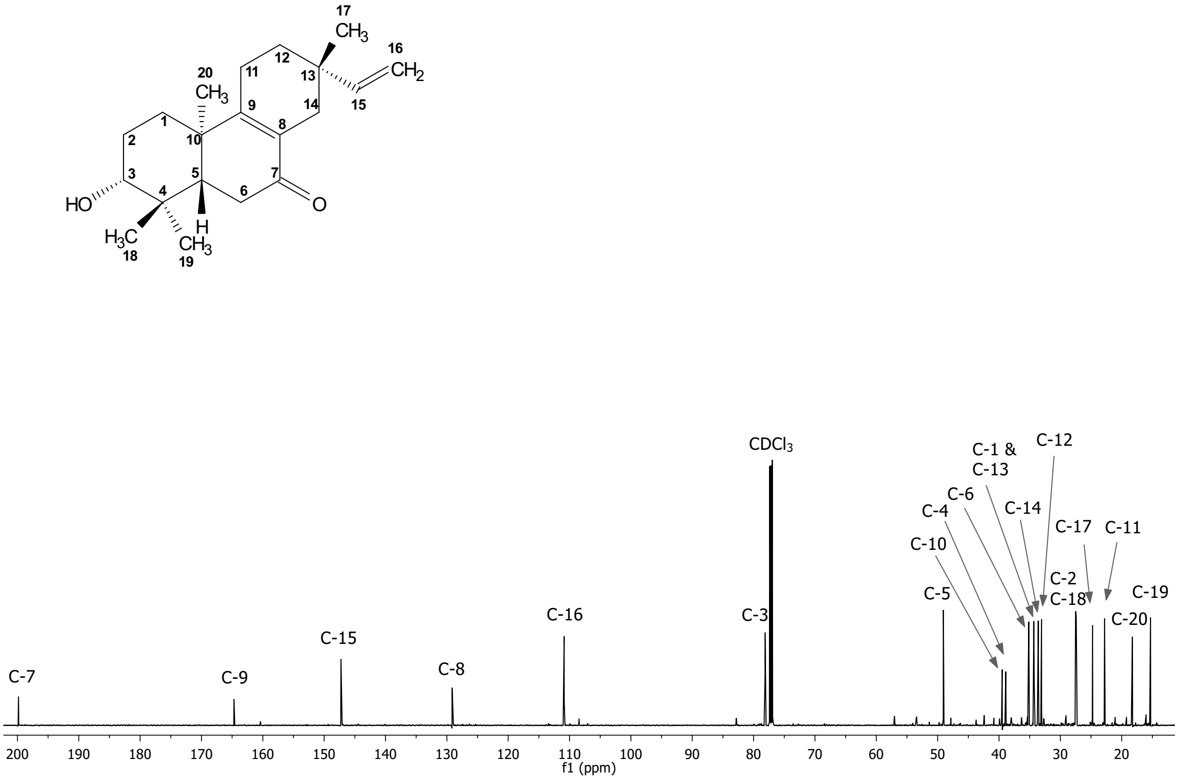

Figure S18. ^{13}C NMR spectrum of compound **3** ($CDCl_3$, 150 MHz).

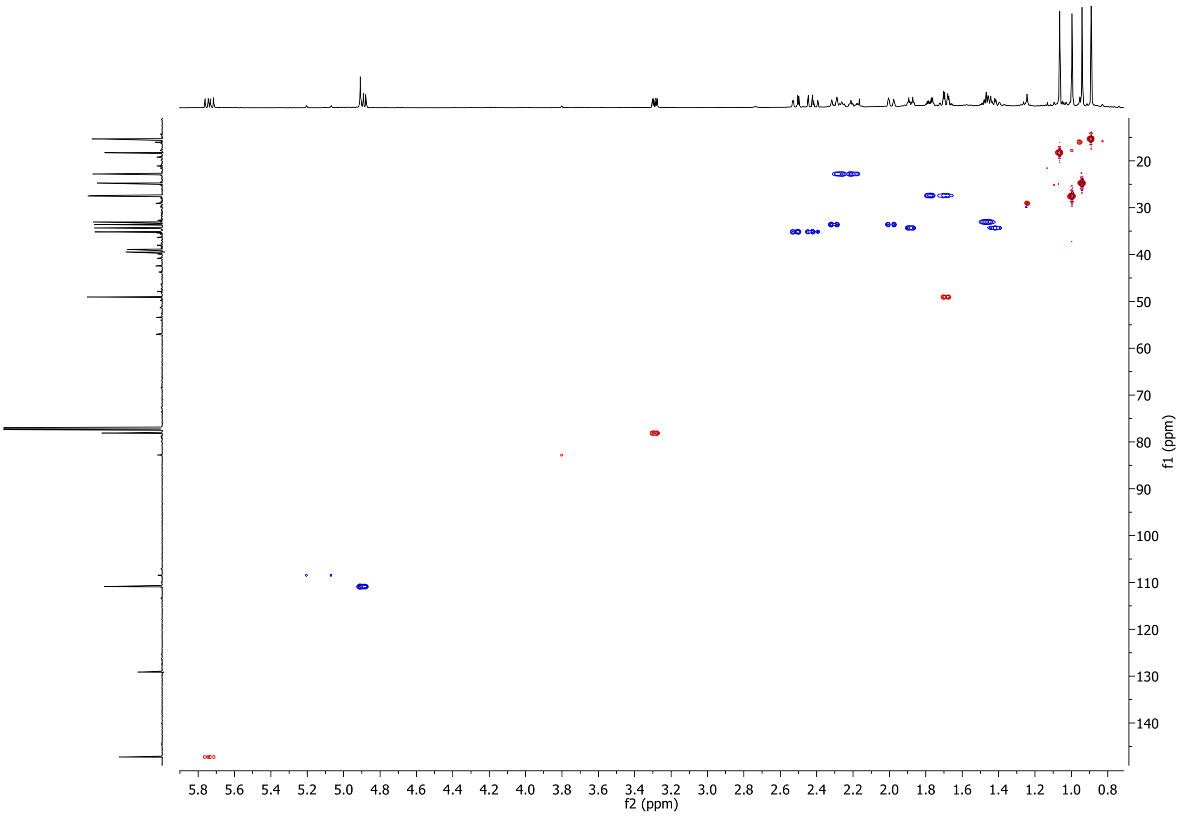

Figure S19. $^{1}H/^{13}$-HSQC spectrum of compound **3** (600/150 MHz, $CDCl_3$).

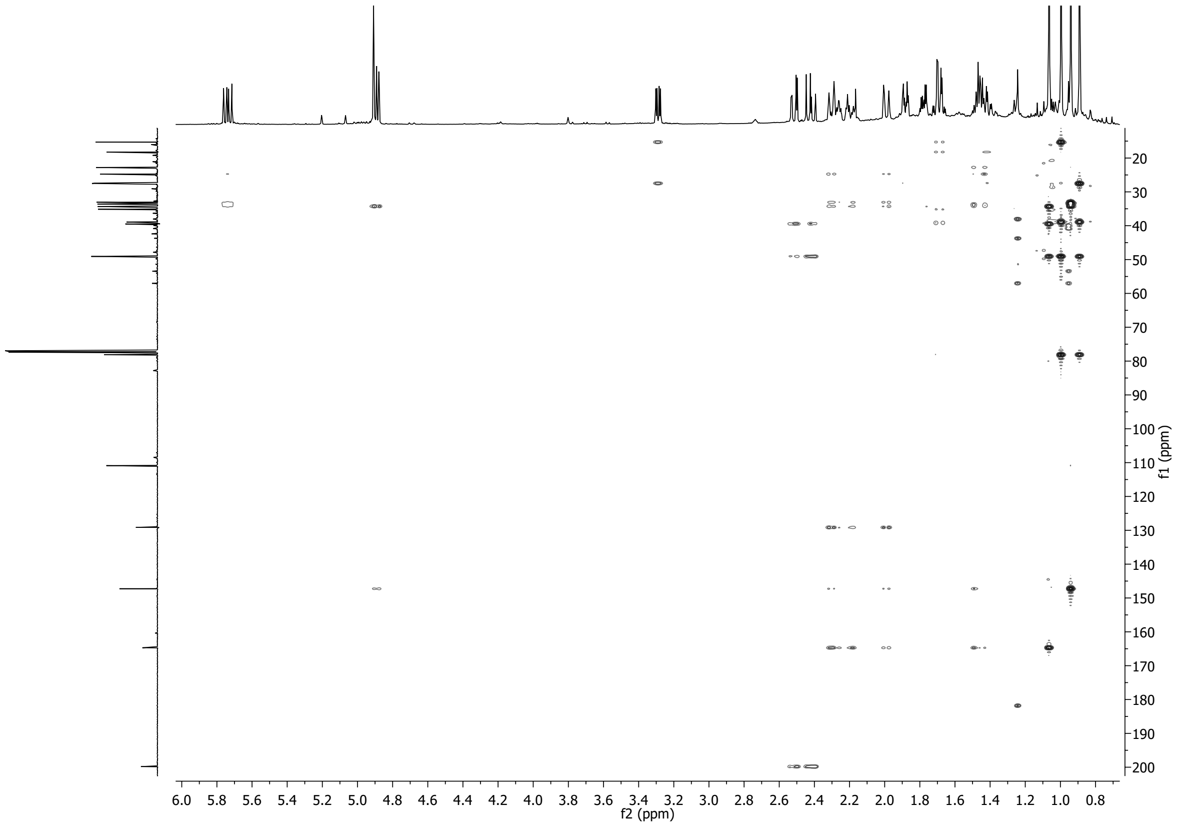

Figure S20. $^{1}H/^{13}$-HMBC spectrum of compound **3** (600/150 MHz, $CDCl_3$).

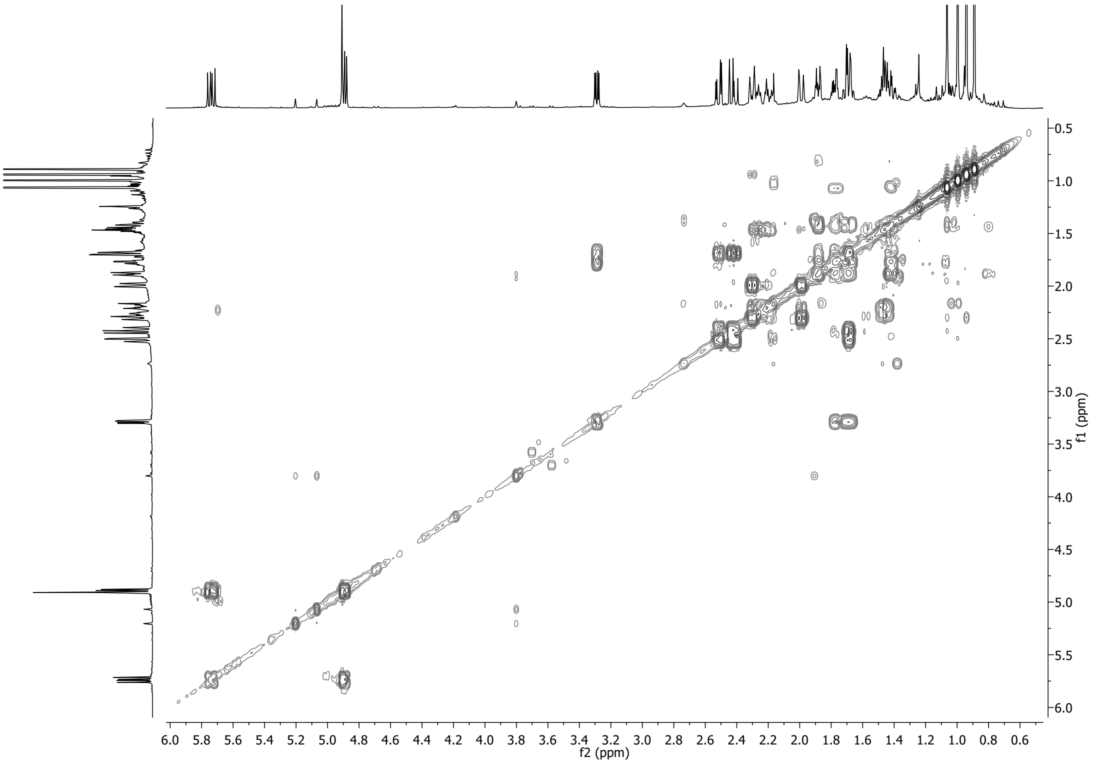

Figure S21. $^1H/^1H$-COSY spectrum of compound **3** (600 MHz, $CDCl_3$).

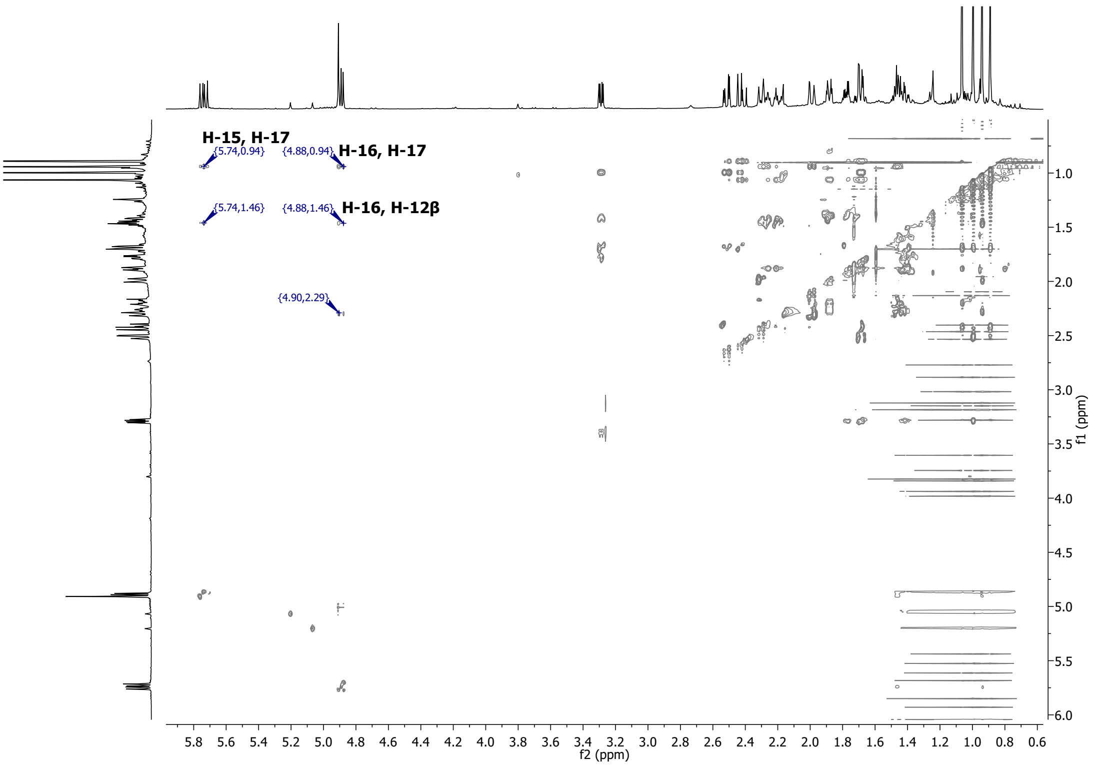

Figure S22. Partial $^1H/^1H$-NOESY spectrum of compound **3** (600 MHz, $CDCl_3$) highlighting correlations in the downfield region.

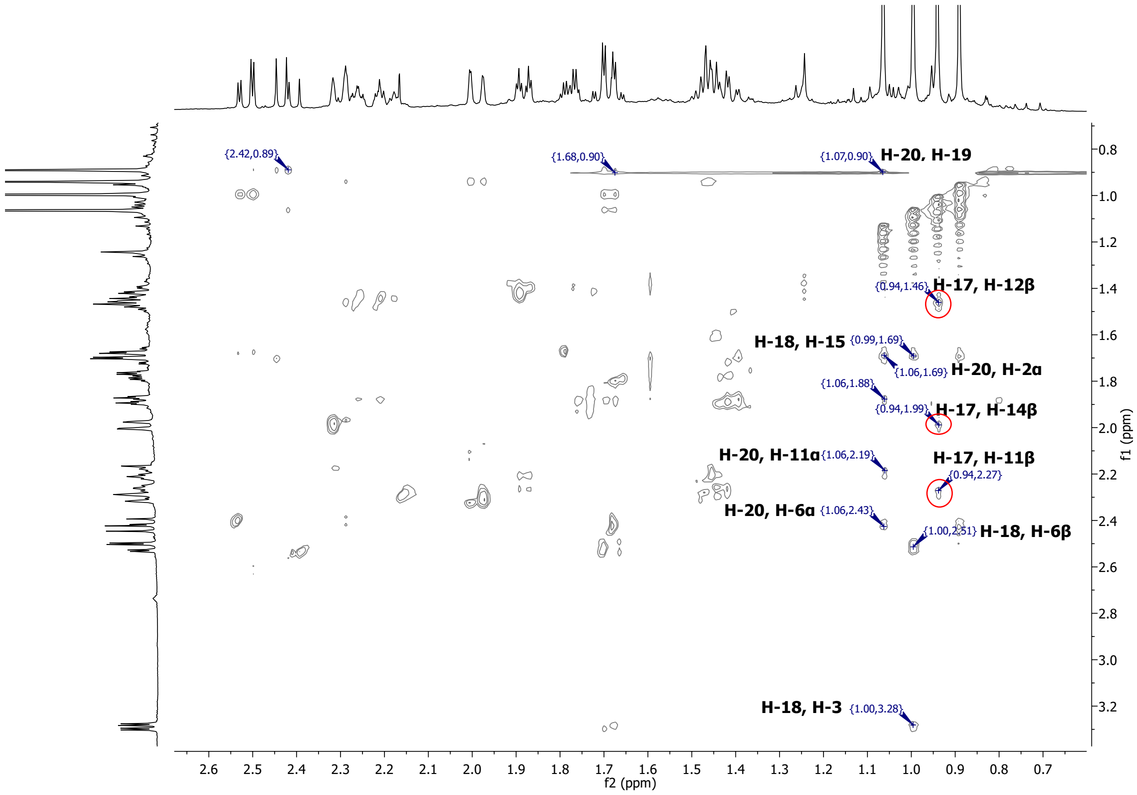

Figure S23. Partial $^{1}H/^{1}H$-NOESY spectrum of compound **3** (600 MHz, $CDCl_3$) showing correlations in the upfield region. Stereochemistry at C-13 in compound **4** is justified by the NOE effects circled in red.

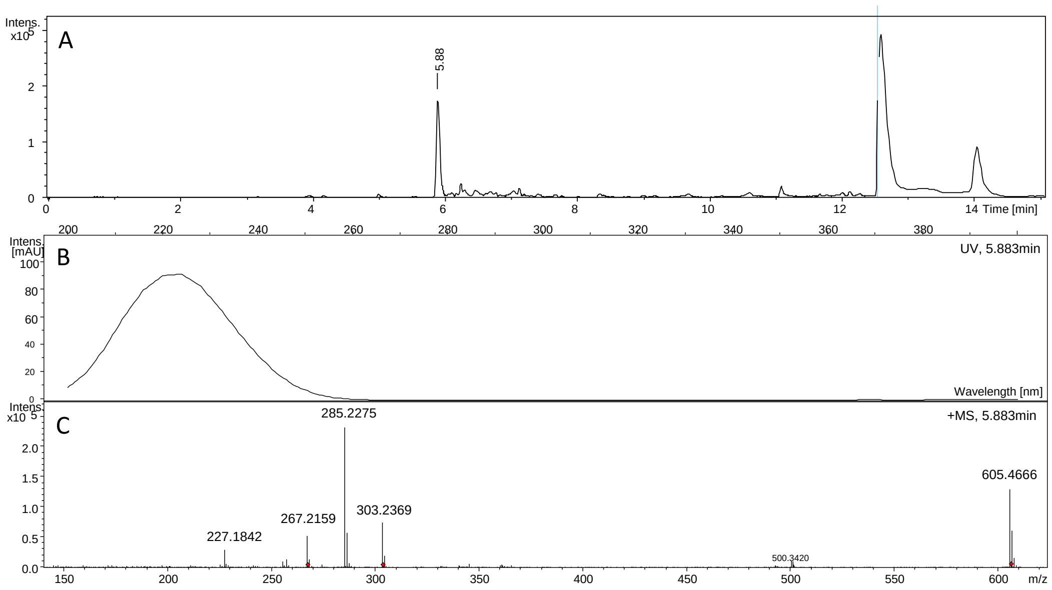

Figure S24. +ESI-QqTOF-MS chromatogram (**A**); UV spectrum (**B**); +ESI-MS (**C**) and +ESI-MS2 (**D**) spectra of compound **4**, $[M + H]^{+}$: 303.2369 ($C_{20}H_{31}O_2$). Internal calibrant sodium formiate: 12.6 min.

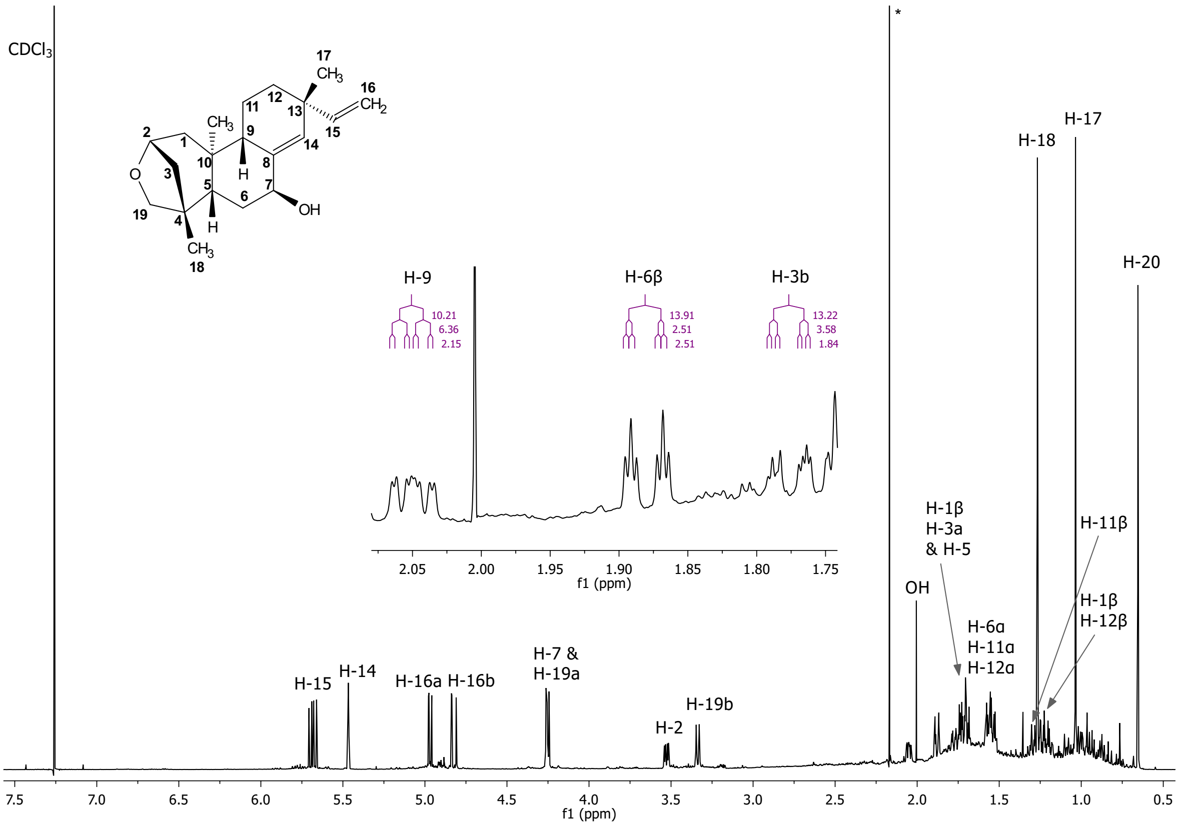

Figure S25. ^{1}H NMR spectrum of compound **4** ($CDCl_3$, 600 MHz). *-CH_3 protons from acetone.

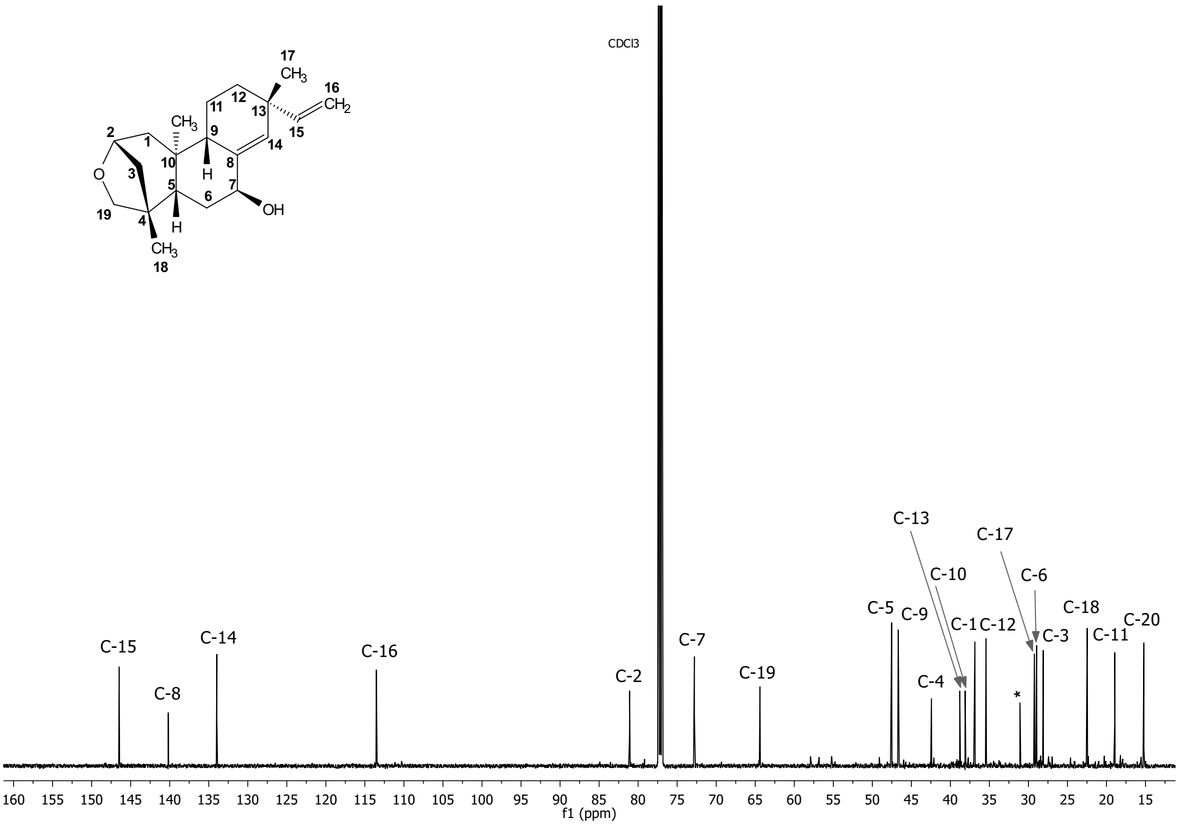

Figure S26. ^{13}C NMR spectrum of compound **4** ($CDCl_3$, 150 MHz). *-CH_3 from acetone.

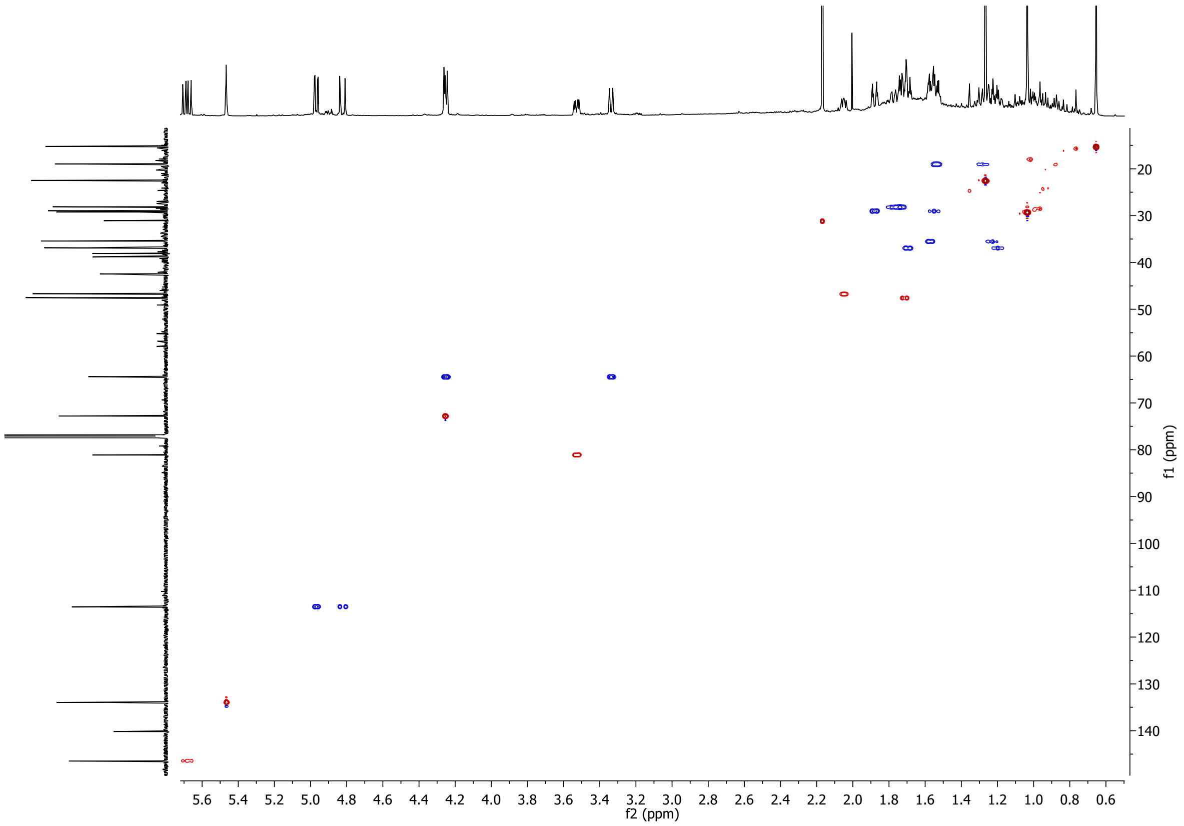

Figure S27. $^{1}H/^{13}C$-HSQC spectrum of compound **4** (600/150 MHz, $CDCl_3$).

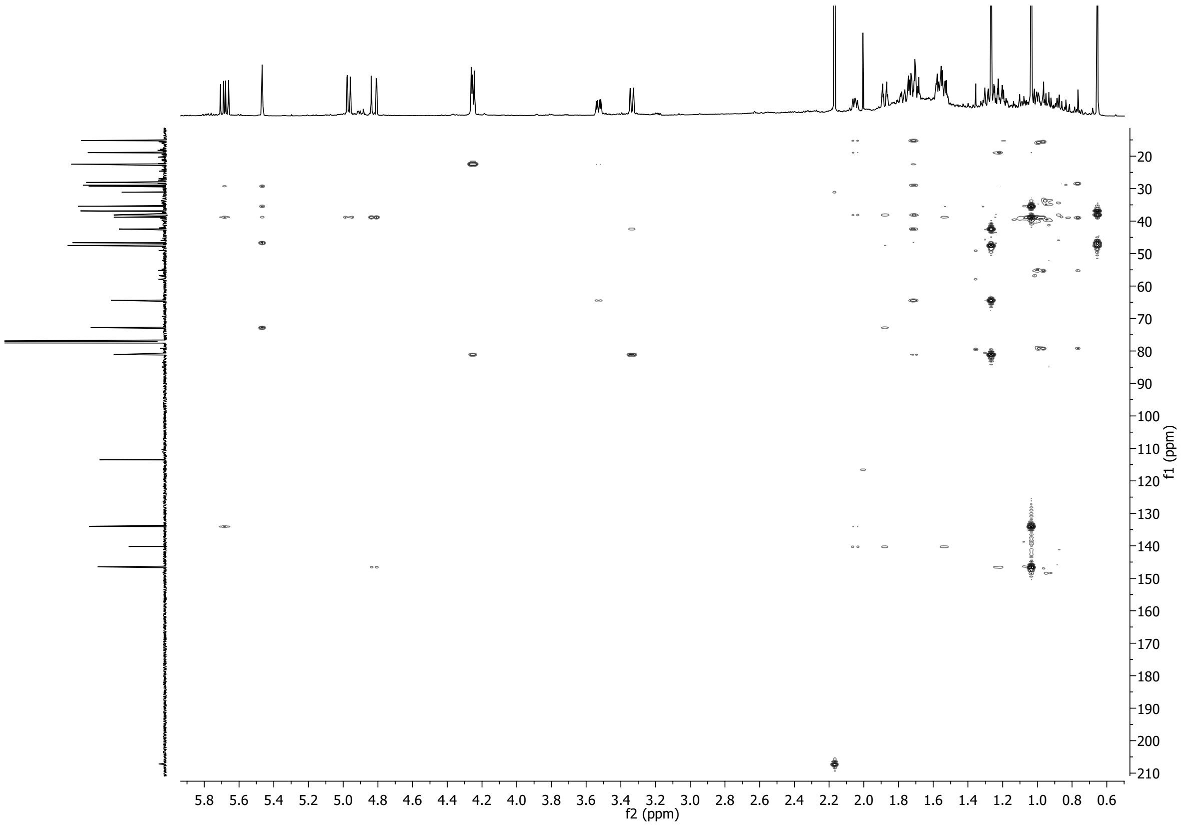

Figure S28. $^{1}H/^{13}C$-HMBC spectrum of compound **4** (600/150 MHz, $CDCl_3$).

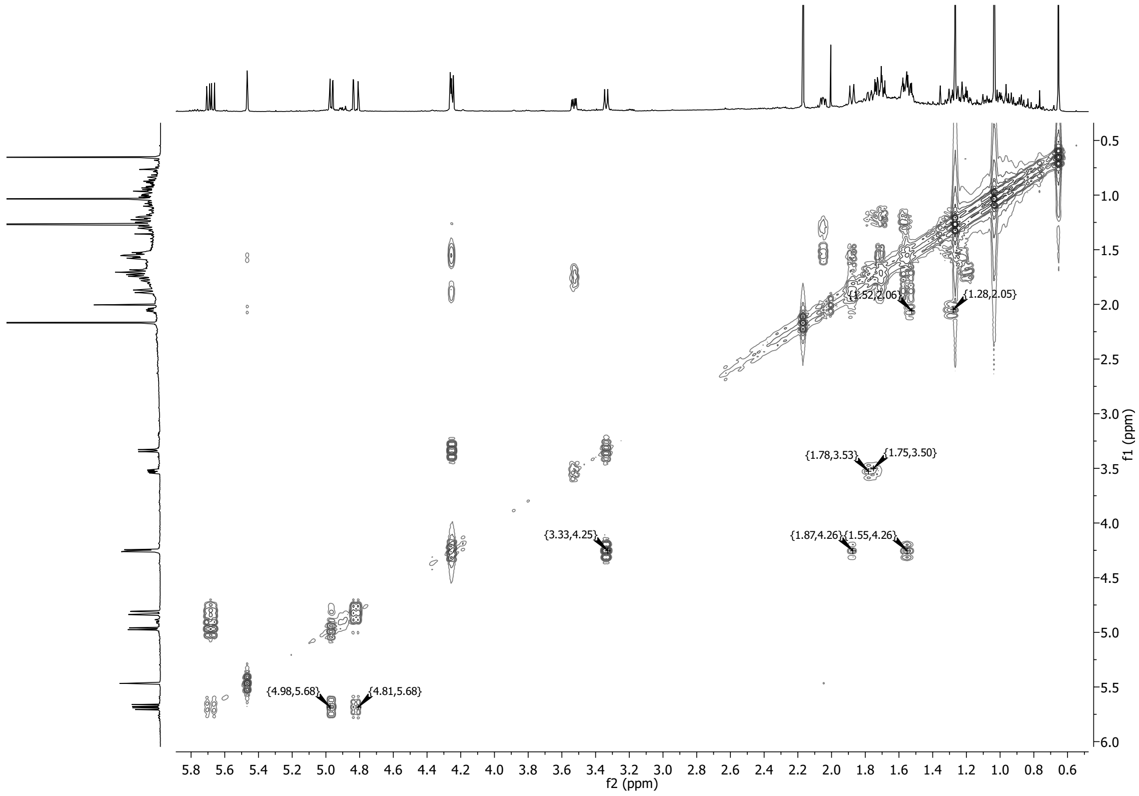

Figure S29. $^1H/^1H$-COSY spectrum of compound **4** (600 MHz, $CDCl_3$).

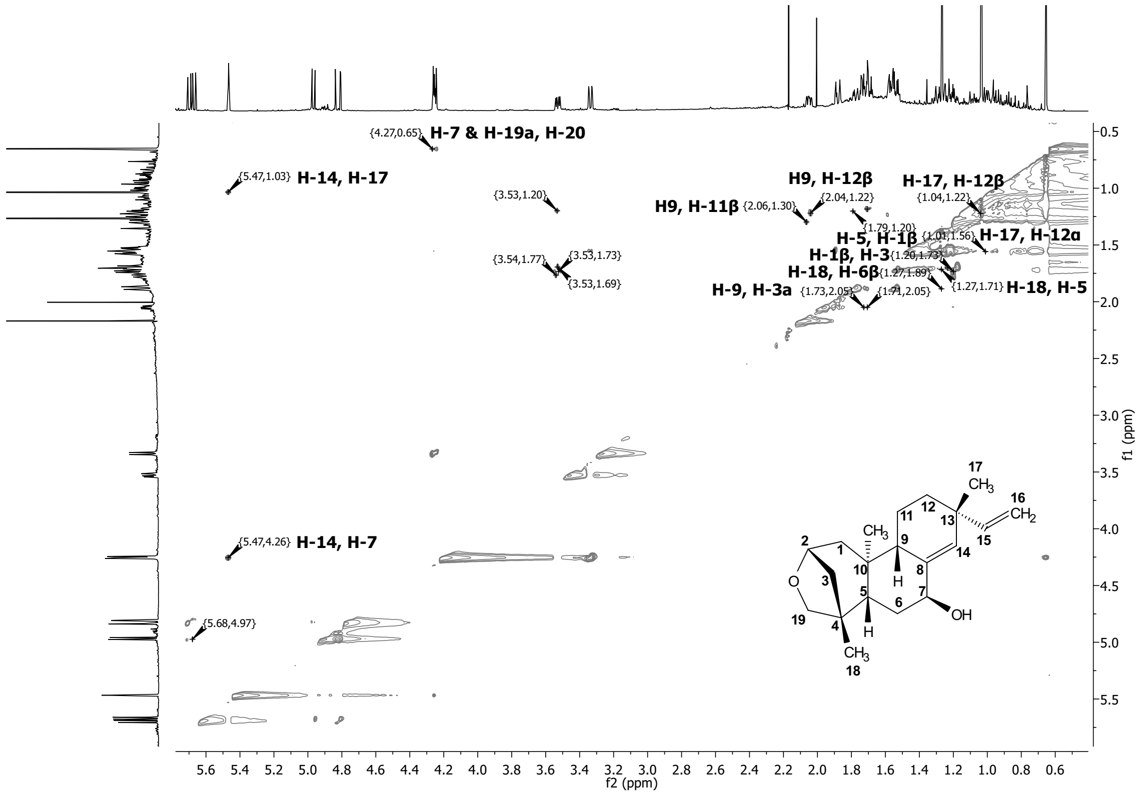

Figure S30. $^1H/^1H$-NOESY spectrum of compound **4** (600 MHz, $CDCl_3$).
