# Supplementary material for: ent-Pimarane and ent-Kaurane Diterpenes from Aldama discolor (Asteraceae) and Their Antiprotozoal Activity
Source: Molecules. 2016 Sep 15;21(9):1237. doi: 10.3390/molecules21091237 (PMC6274167; doi:10.3390/molecules21091237)
Supplement: Supplementary file 2 [file molecules-21-01237-s002.zip › Fig9_1H_NMR_S_14.pdf]

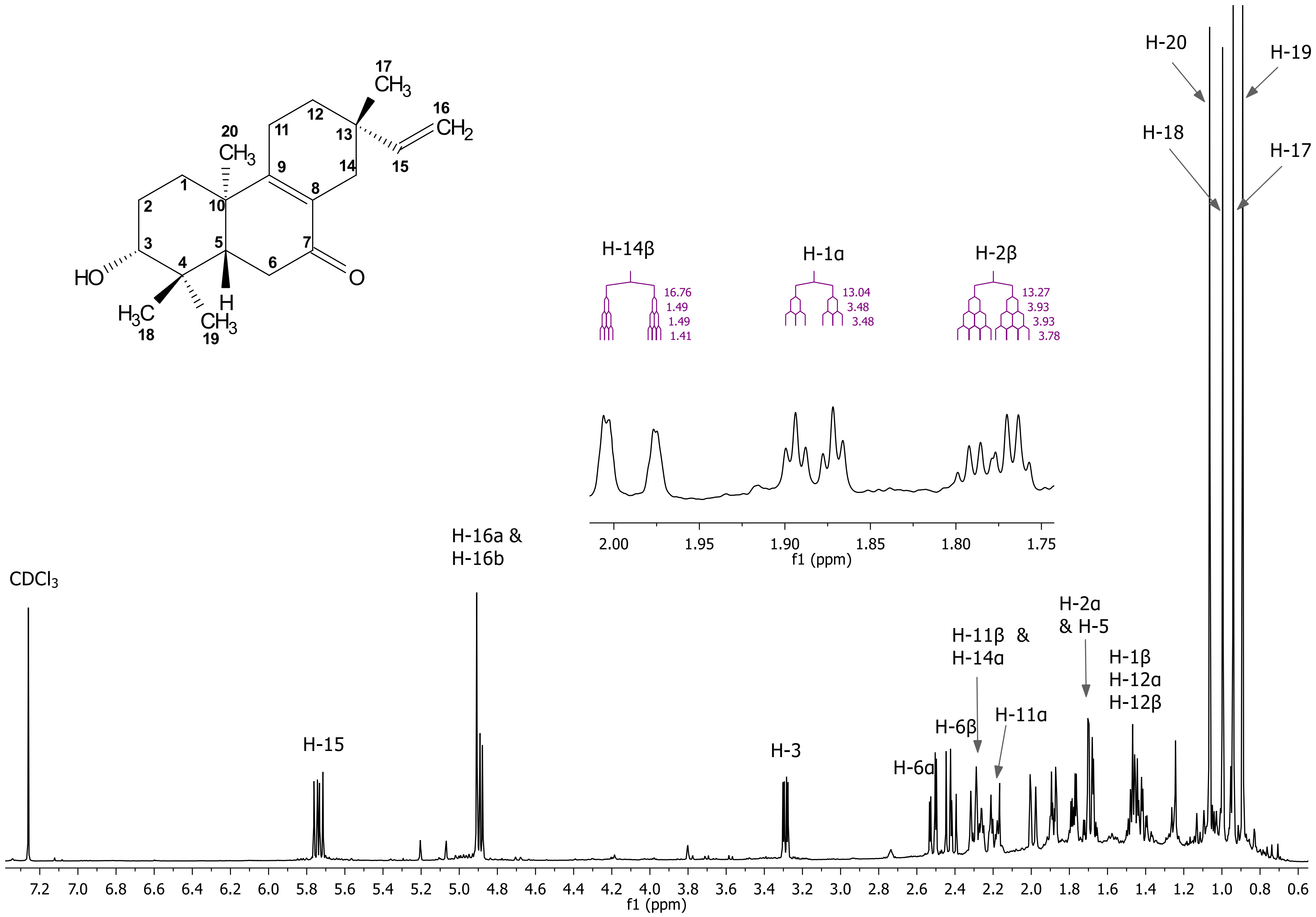
17
CH3
16
CH2
20
CH3
11
12
13
14
15
9
8
1
2
10
3
5
7
4
6
HO
O
H
H3C
CH3
18
19
H-14β
16.76
1.49
1.49
1.41
H-1α
13.04
3.48
3.48
H-2β
13.27
3.93
3.93
3.78
2.00
1.95
1.90
1.85
1.80
1.75
f1 (ppm)
H-20
H-19
H-18
H-17
CDCl3
H-16a &
H-16b
H-15
H-3
H-6α
H-6β
H-11β &
H-14α
H-11α
H-2α
& H-5
H-1β
H-12α
H-12β
7.2
7.0
6.8
6.6
6.4
6.2
6.0
5.8
5.6
5.4
5.2
5.0
4.8
4.6
4.4
4.2
4.0
3.8
3.6
3.4
3.2
3.0
2.8
2.6
2.4
2.2
2.0
1.8
1.6
1.4
1.2
1.0
0.8
0.6
f1 (ppm)
