# Supplementary material for: ent-Pimarane and ent-Kaurane Diterpenes from Aldama discolor (Asteraceae) and Their Antiprotozoal Activity
Source: Molecules. 2016 Sep 15;21(9):1237. doi: 10.3390/molecules21091237 (PMC6274167; doi:10.3390/molecules21091237)
Supplement: Supplementary file 2 [file molecules-21-01237-s002.zip › logo-mdpi-eps-converted-to.pdf]

MDPI
