# Supplementary material for: ent-Pimarane and ent-Kaurane Diterpenes from Aldama discolor (Asteraceae) and Their Antiprotozoal Activity
Source: Molecules. 2016 Sep 15;21(9):1237. doi: 10.3390/molecules21091237 (PMC6274167; doi:10.3390/molecules21091237)
Supplement: Supplementary file 2 [file molecules-21-01237-s002.zip › Supporting_Information.pdf]

Article

ent-Pimarane and *ent*-Kaurane Diterpenes from *Aldama discolor* (Asteraceae) and their Antiprotozoal Activity

Mauro S. Nogueira[1], Fernando B. Da Costa[2], Reto Brun[3,4], Marcel Kaiser[3,4] and Thomas J. Schmidt[1,*]

1 Institute of Pharmaceutical Biology and Phytochemistry (IPBP), University of Münster, PharmaCampus Corrensstraße 48,Münster D-48149, Münster, Germany; nogueira@uni-muenster.de (M.N.); thomschm@uni-muenster.de (T.J.S.)
2 AsterBioChem Research Team, Laboratory of Pharmacognosy, School of Pharmaceutical Sciences of Ribeirão Preto, USP, Av. do Café s/n, 14040-903, Ribeirão Preto-SP, Brazil; febcosta@fcfrp.usp.br
3 Swiss Tropical and Public Health Institute (Swiss TPH), Socinstr. 57, Basel CH-4051, Switzerland; marcel.kaiser@unibas.ch
4 University of Basel, Petersplatz 1, Basel CH-4003, Switzerland
* Correspondence: thomschm@uni-muenster.de; Tel.: +49-251-83-33378

Academic Editor: Dereck J. McPhee
Version August 31, 2016 submitted to Molecules; Typeset by LaTeX using class file mdpi.cls

1. Supporting Information

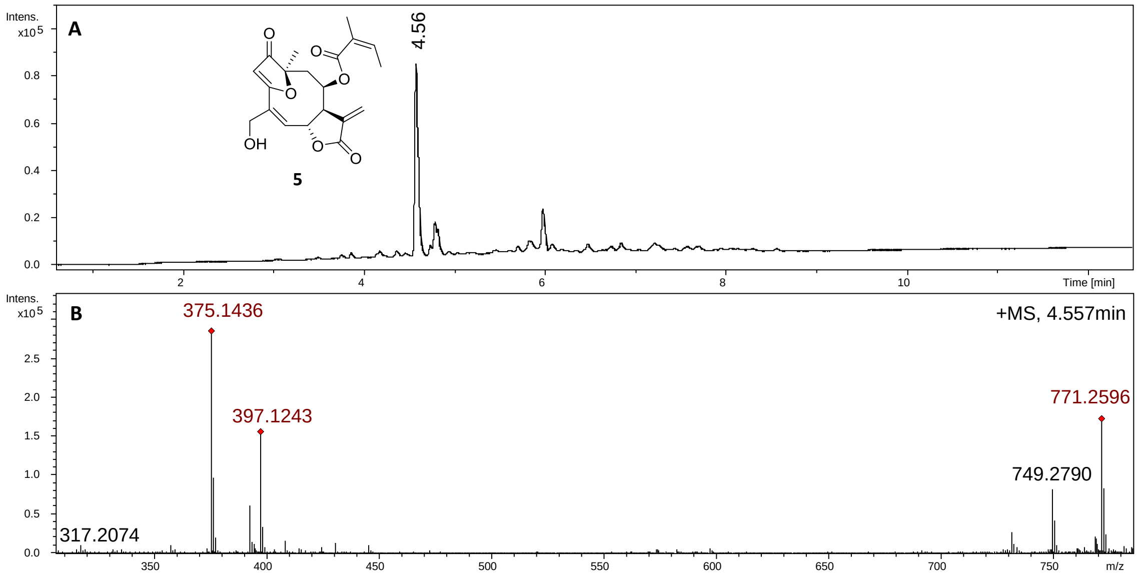

Figure S1. A: LC-MS-chromatogram of the last fraction (elution with EtOAc) of the CC on silica gel of the dichloromethane extract of *A. discolor* leaves. B: +ESI-MS of its major compound, budlein A. $[M+H]^+$: 375.1436 ($C_{20}H_{23}O_7$).

© 2016 by the authors. Submitted to *Molecules* for possible open access publication under the terms and conditions of the Creative Commons Attribution license (http://creativecommons.org/licenses/by/4.0/).

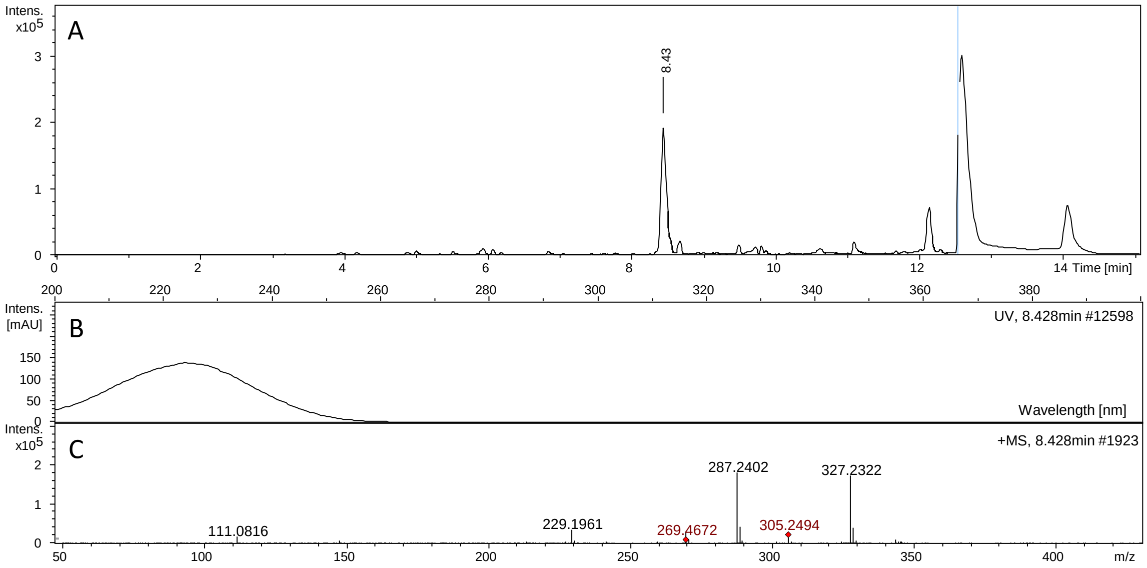

Figure S2. +ESI-QqTOF-MS chromatogram (A), UV spectrum (B), +ESI-MS (C) of compound **1**, $[M+H]^+$: 305.2494 ($C_{20}H_{33}O_2$) and $[M+Na]^+$: 327.2322 ($C_{20}H_{32}O_2Na$). Internal calibrant sodium formiate: 12.6 min.

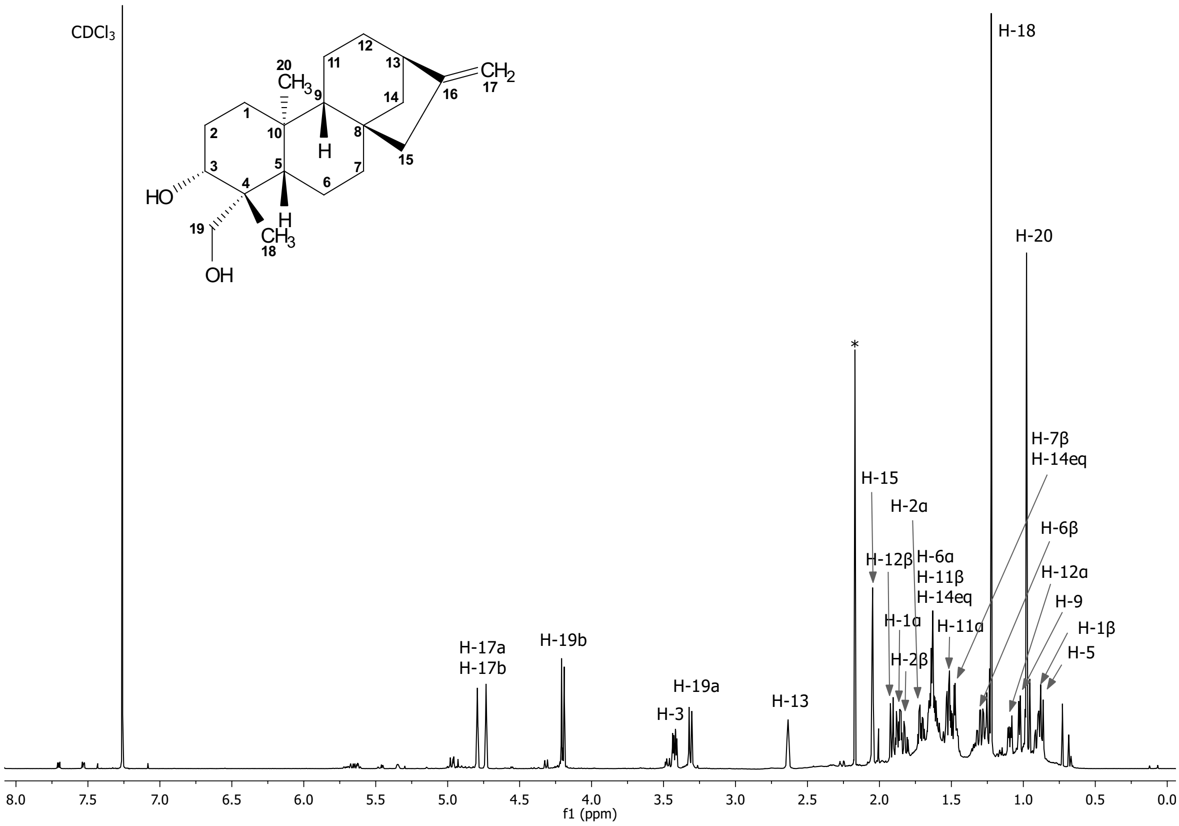

Figure S3. ^1H NMR spectrum of compound **1** ($CDCl_3$, 600 MHz). *-CH_3 protons from acetone.

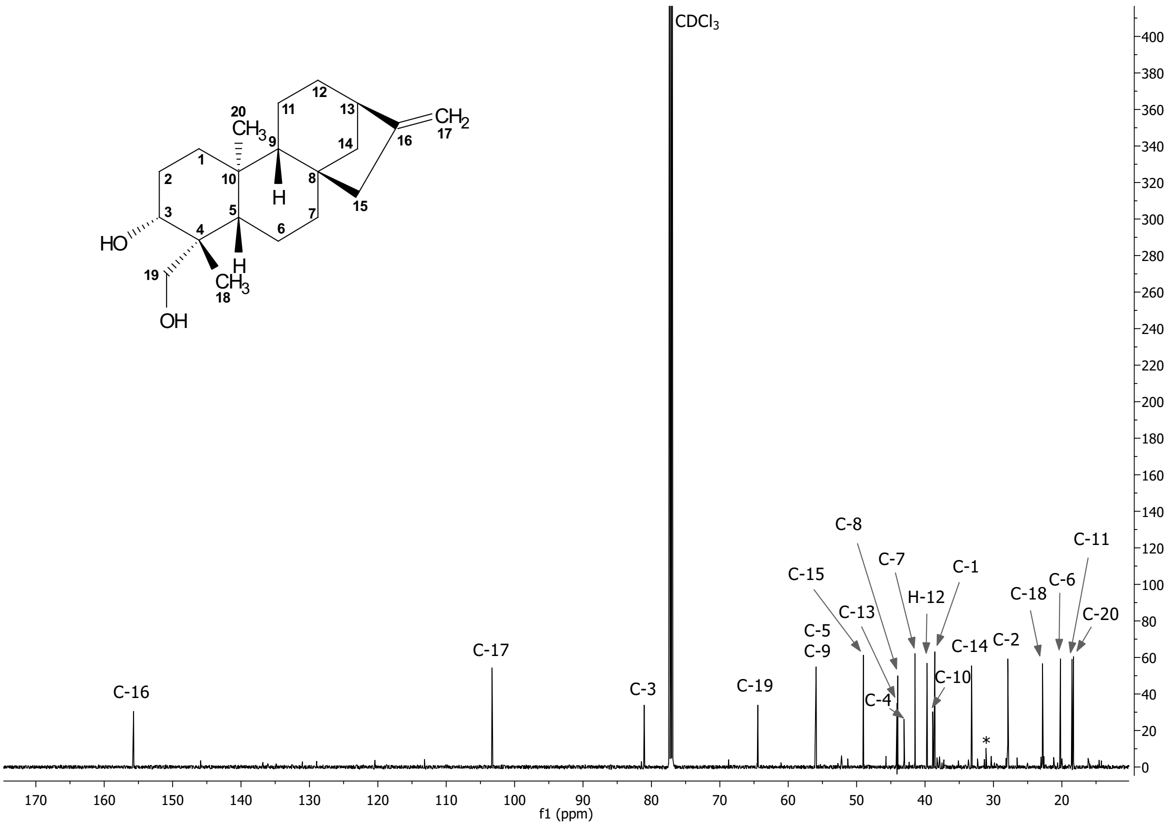

Figure S4. ^{13}C NMR spectrum of compound **1** ($CDCl_3$, 150 MHz). *-CH_3 from acetone.

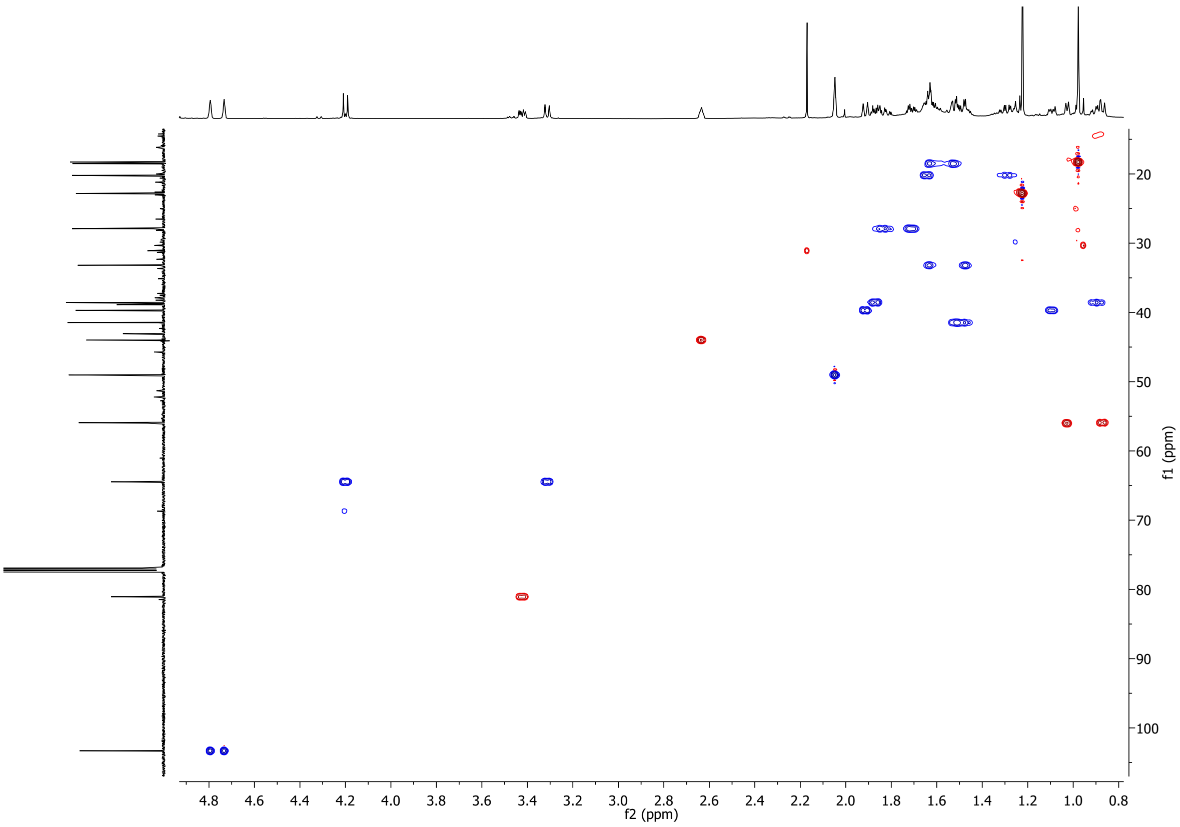

Figure S5. ^{1}H/^{13}-HSQC spectrum of compound **1** (600/150 MHz, $CDCl_3$).

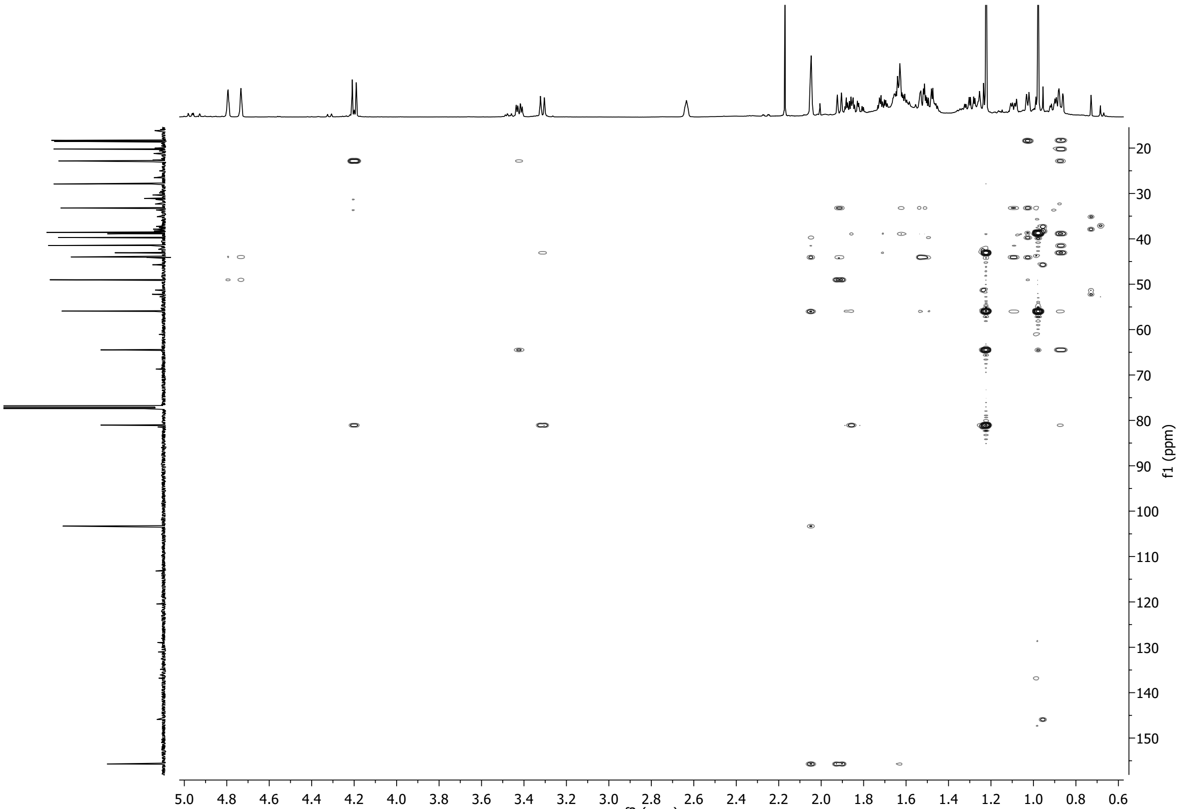

Figure S6. $^{1}H/^{13}$-HMBC spectrum of compound **1** (600/150 MHz, $CDCl_3$).

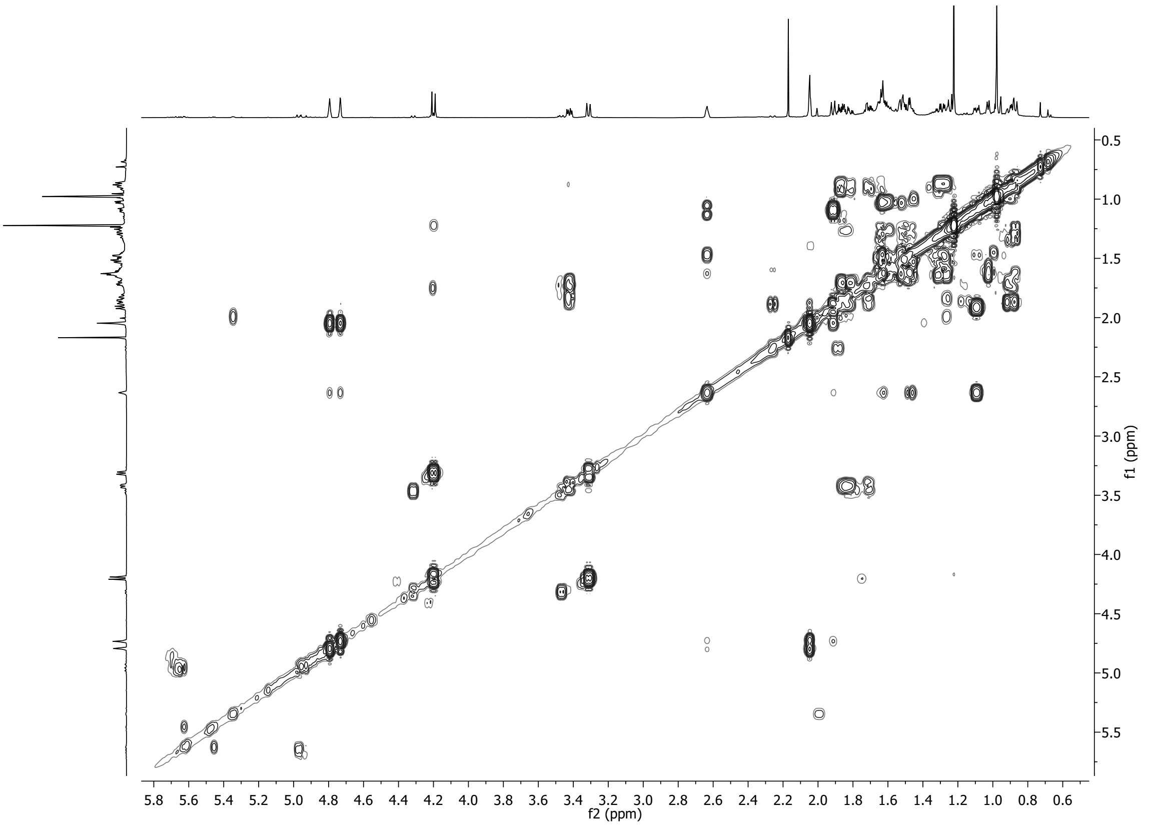

Figure S7. $^{1}H/^{1}H$-COSY spectrum of compound **1** (600 MHz, $CDCl_3$).

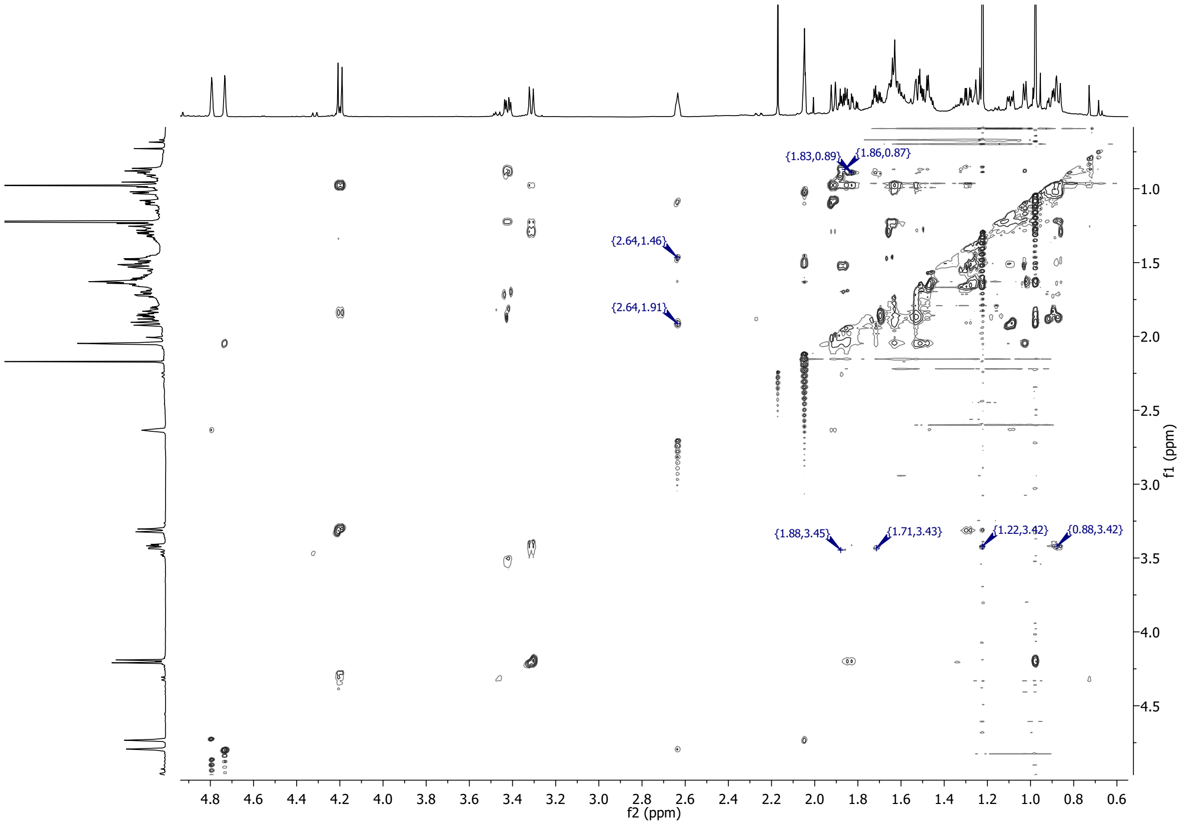

Figure S8. $^1H/^1H$-NOESY spectrum of compound **1** (600 MHz, $CDCl_3$).

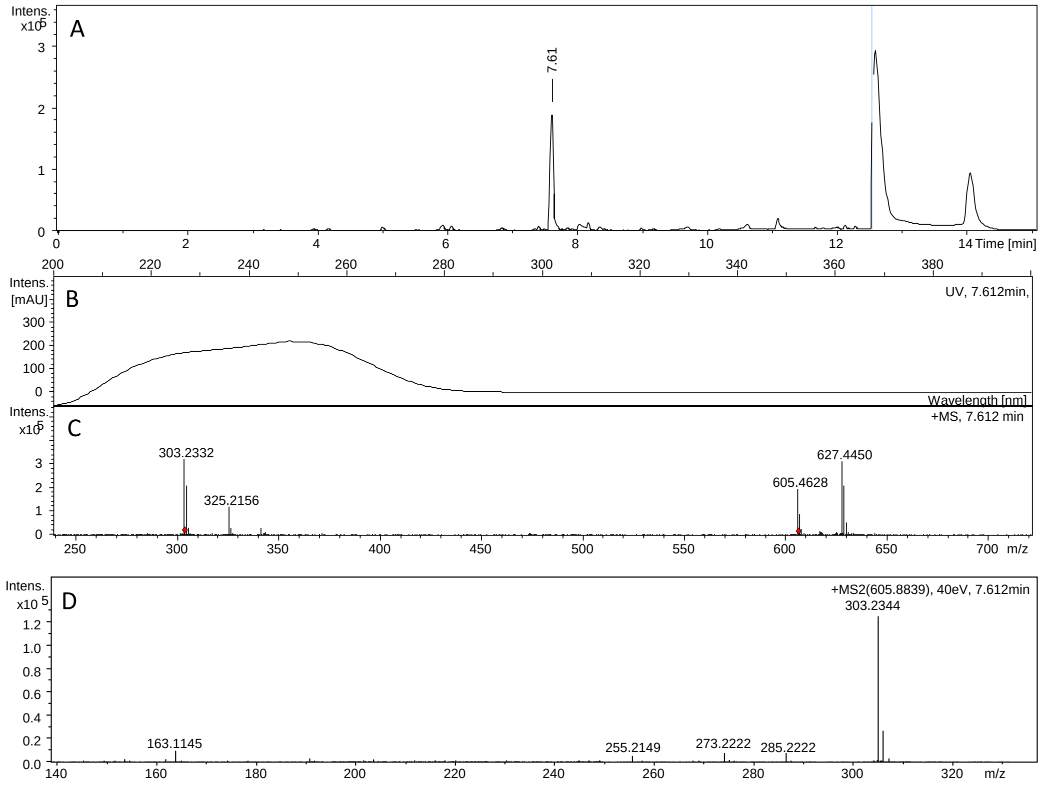

Figure S9. +ESI-QqTOF-MS chromatogram (A), UV spectrum (B), +ESI-MS (C) and +ESI-MS2 (D) spectra of compound **2**, $[M+H]^+$: 303.2346 ($C_{20}H_{31}O_2$). Internal calibrant sodium formiate: 12.6 min.

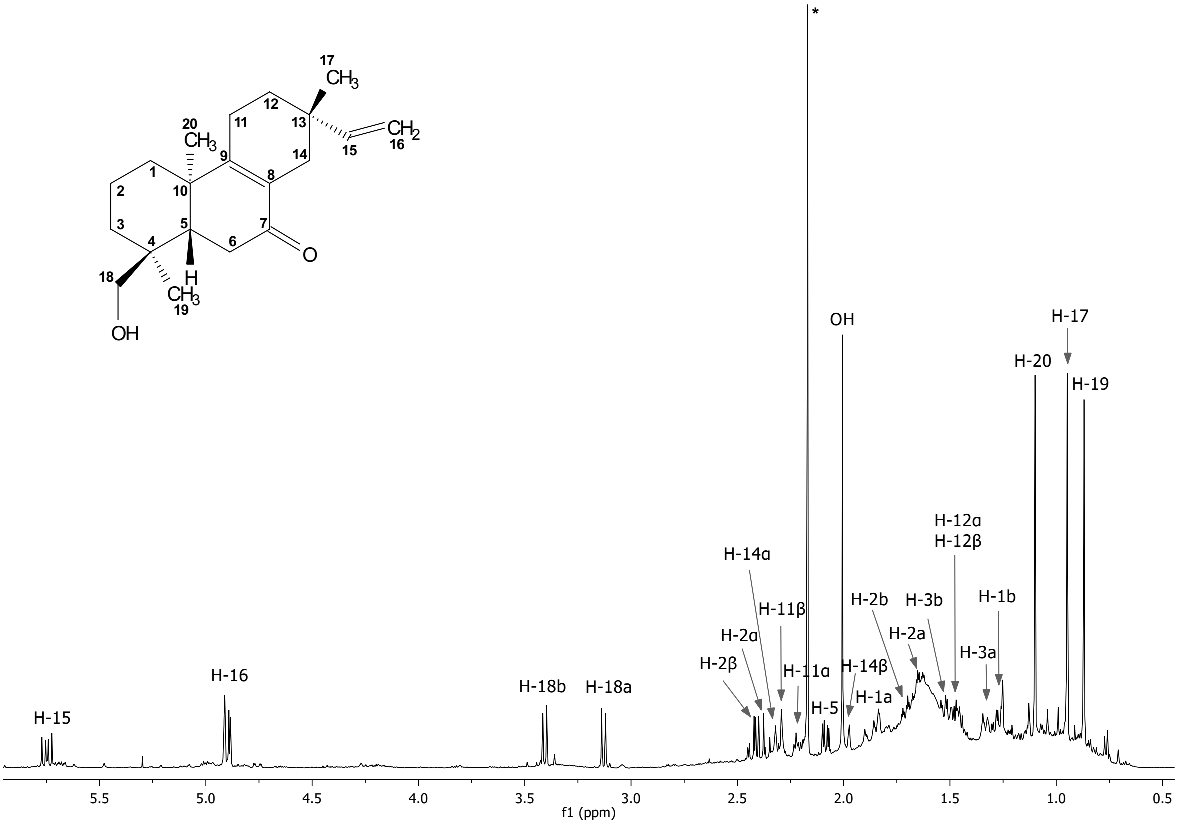

Figure S10. ^{1}H NMR spectrum of compound **2** ($CDCl_3$, 600 MHz). *-CH_3 protons from acetone.

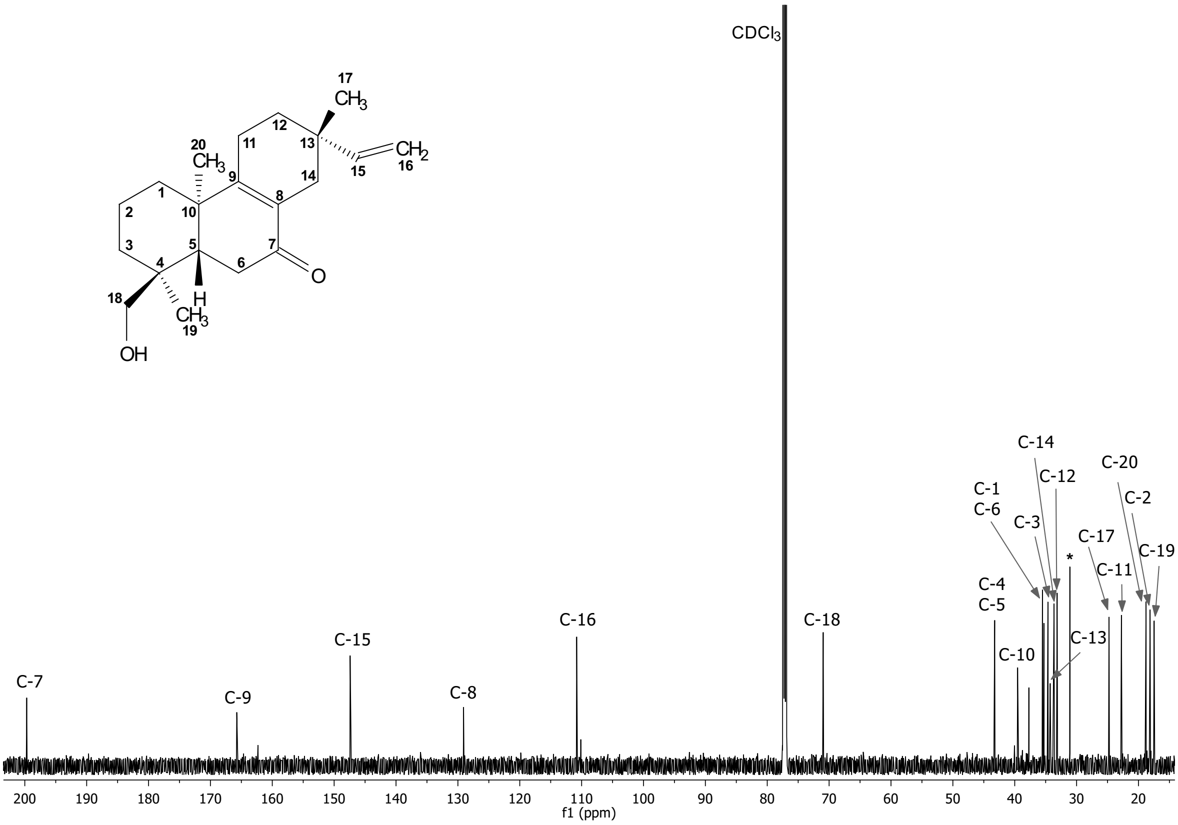

Figure S11. ^{13}C NMR spectrum of compound **2** ($CDCl_3$, 150 MHz). *-CH_3 from acetone.

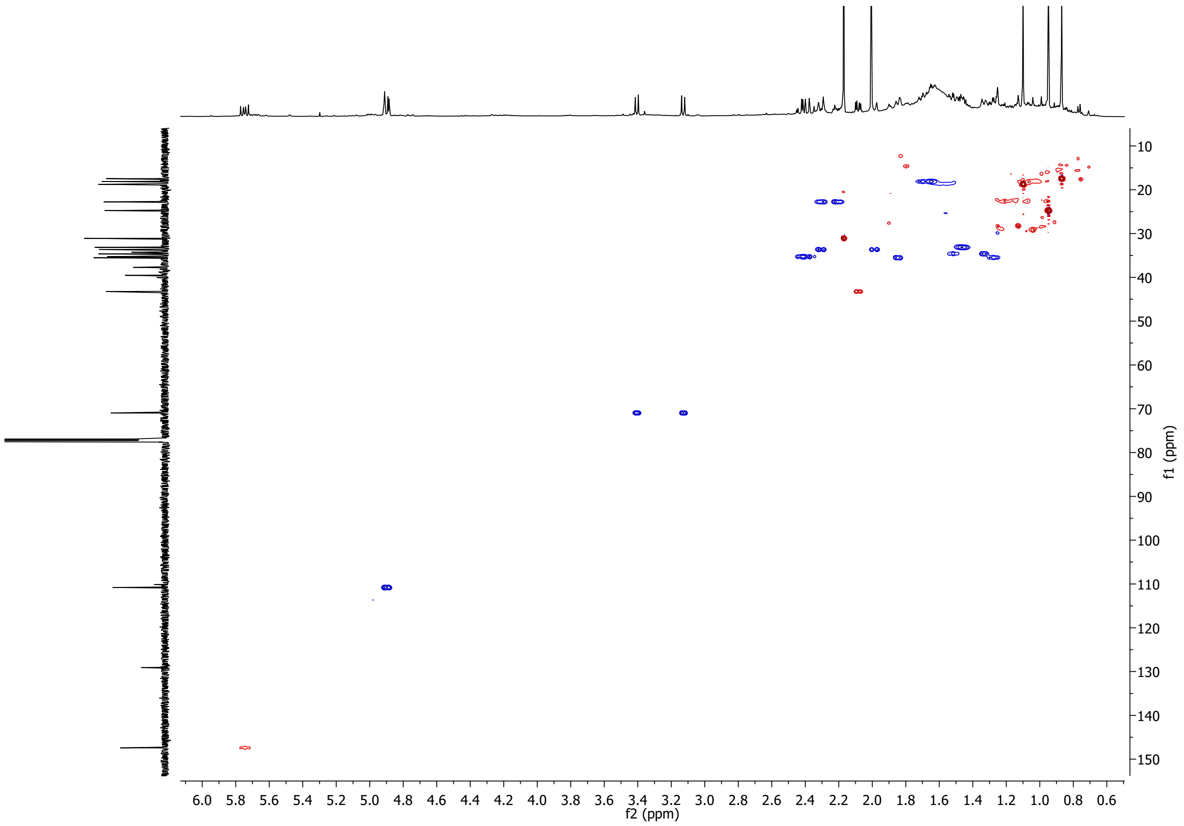

Figure S12. $^{1}H/^{13}$-HSQC spectrum of compound **2** (600/150 MHz, $CDCl_3$).

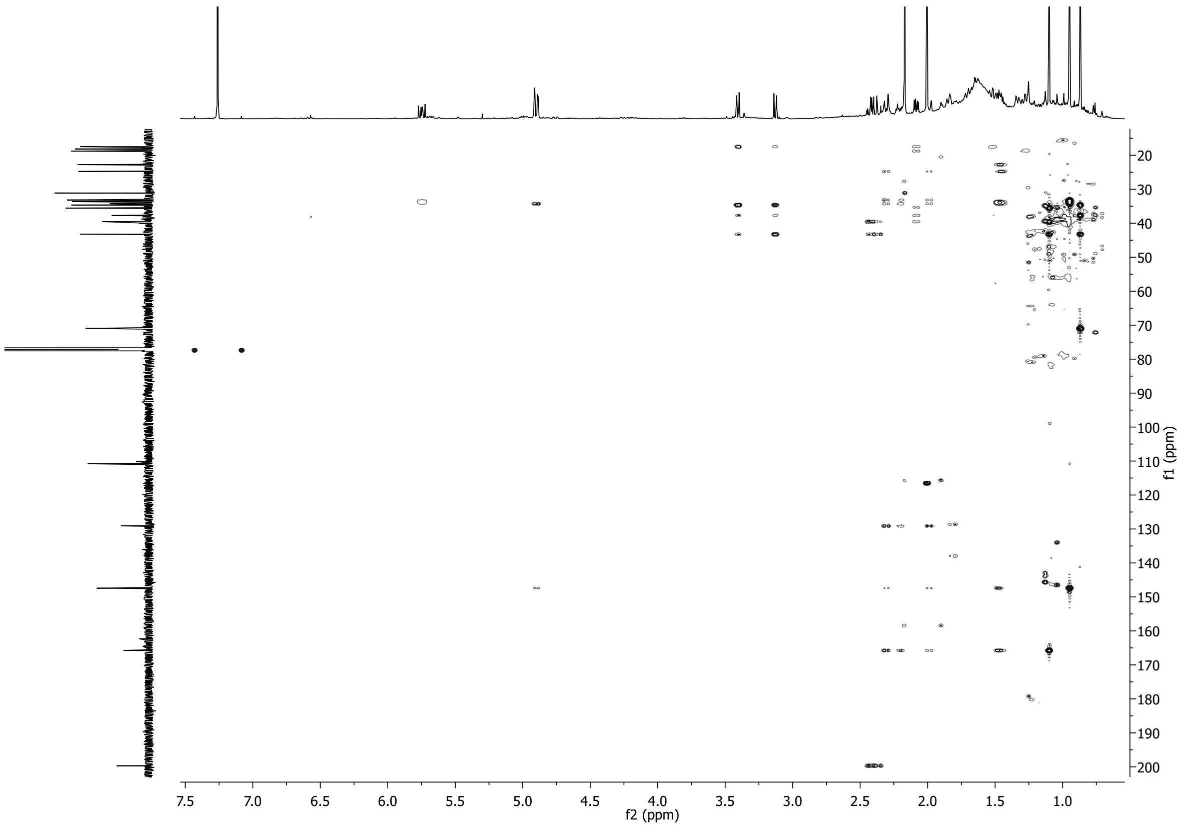

Figure S13. $^{1}H/^{13}$-HMBC spectrum of compound **2** (600/150 MHz, $CDCl_3$).

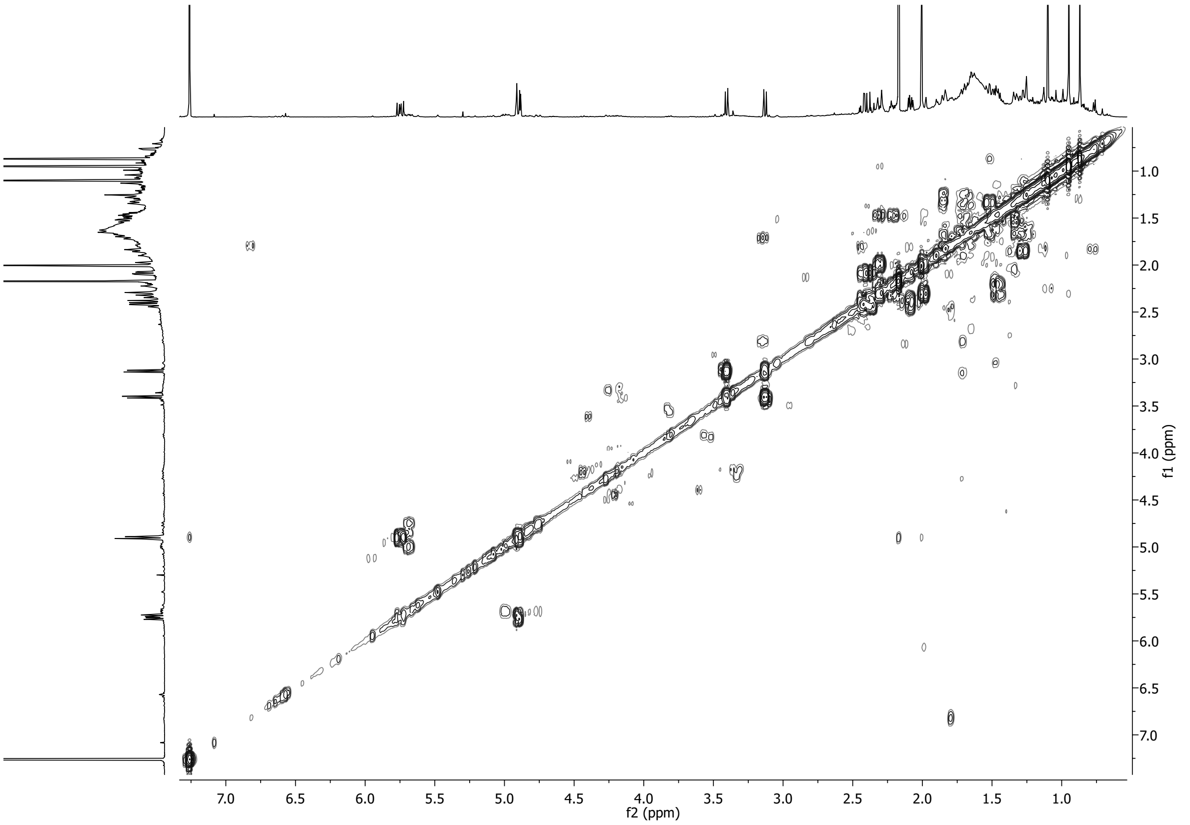

Figure S14. $^1H/^1H$-COSY spectrum of compound **2** (600 MHz, $CDCl_3$).

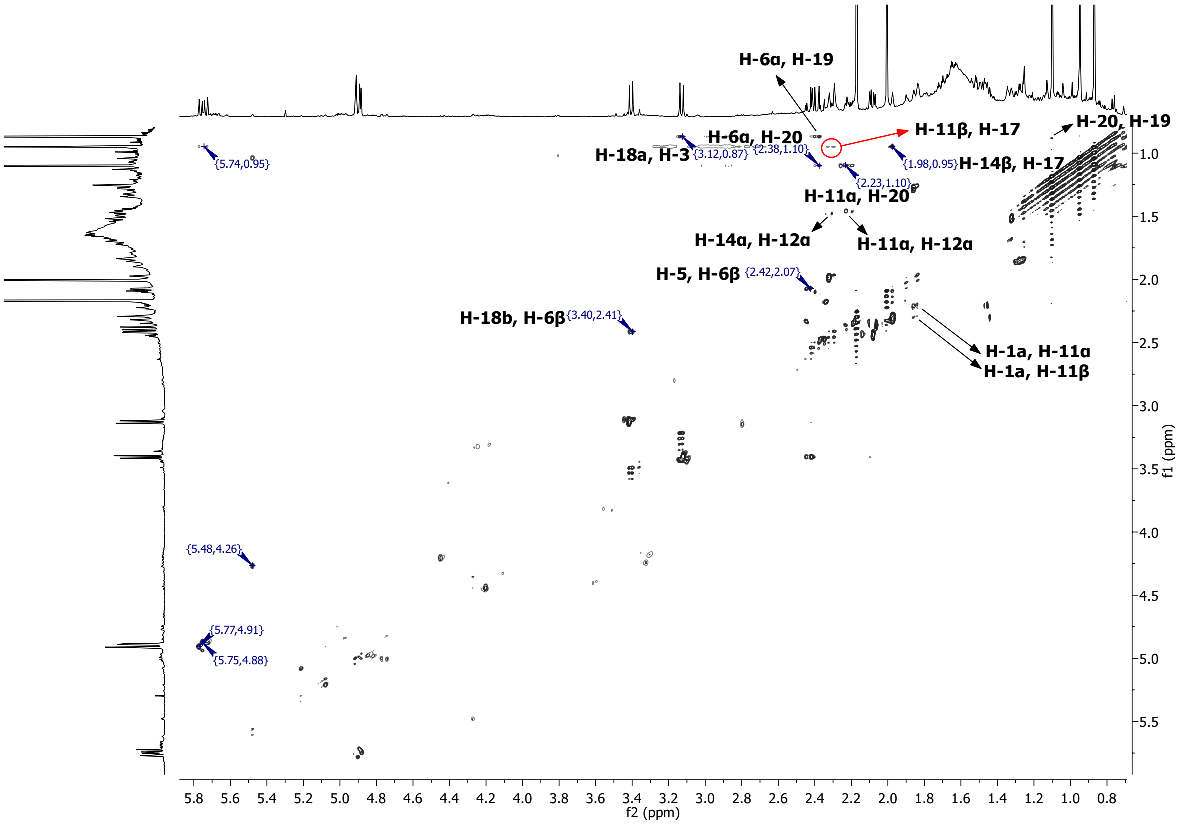

Figure S15. $^1H/^1H$-NOESY spectrum of compound **2** (600 MHz, $CDCl_3$). Cross-correlated peak (red circle) enabled the determination of the stereochemistry at C-13.

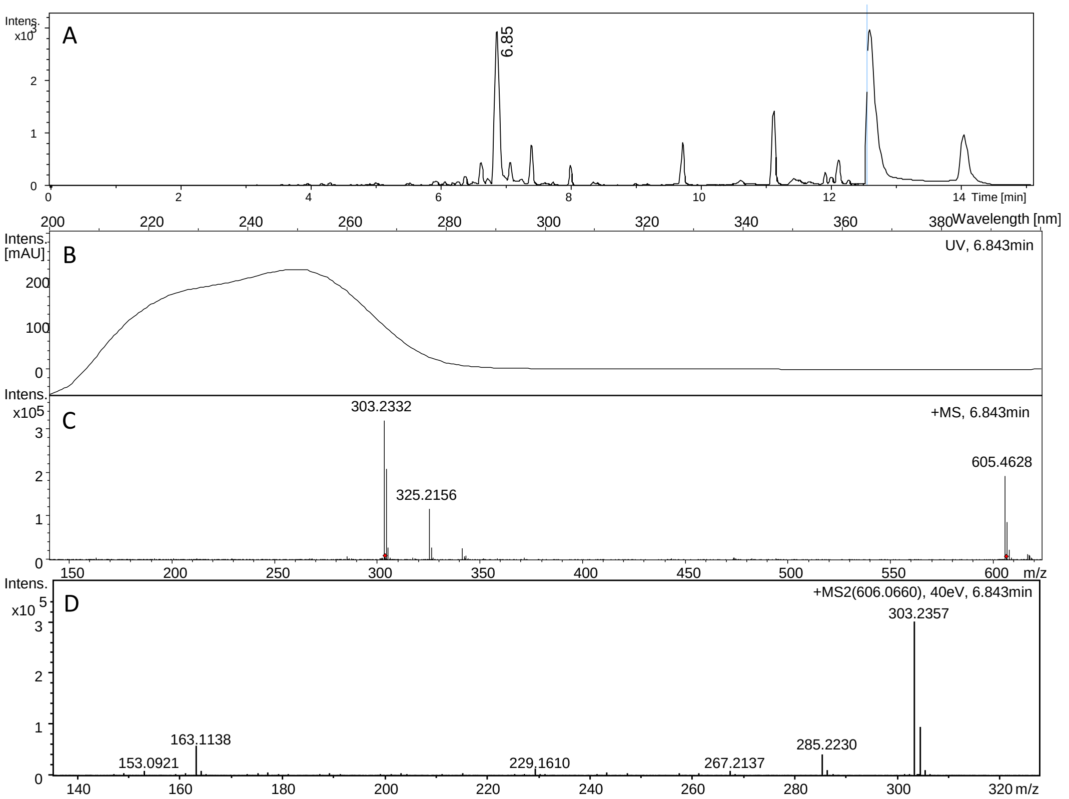

Figure S16. +ESI-QqTOF-MS chromatogram (A), UV spectrum (B), +ESI-MS (C) and +ESI-MS2 (D) spectra of compound **3**, $[M+H]^+$: 303.2332 ($C_{20}H_{31}O_2$). Internal calibrant sodium formiate: 12.6 min.

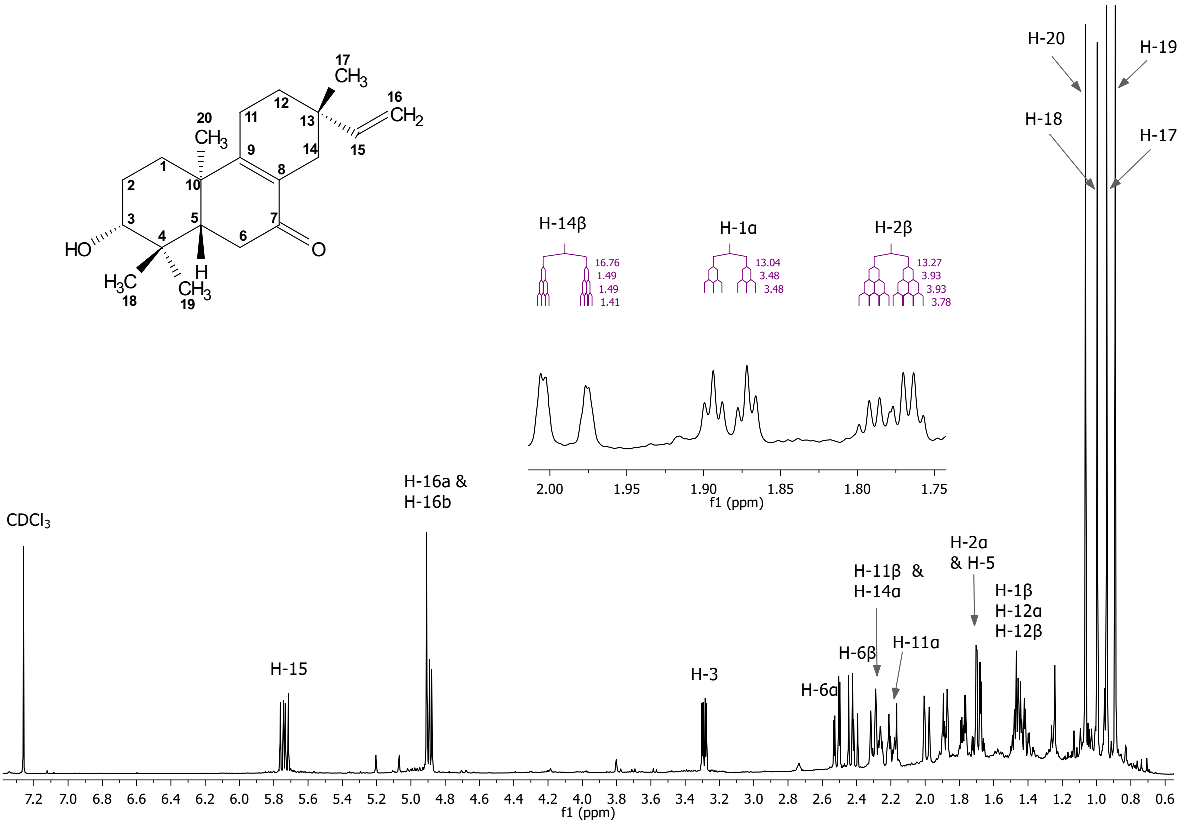

Figure S17. ^1H NMR spectrum of compound **3** ($CDCl_3$, 600 MHz).

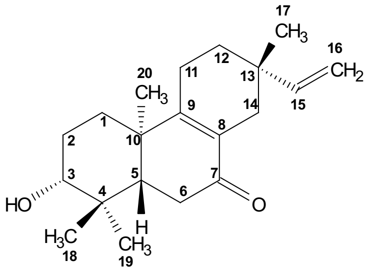

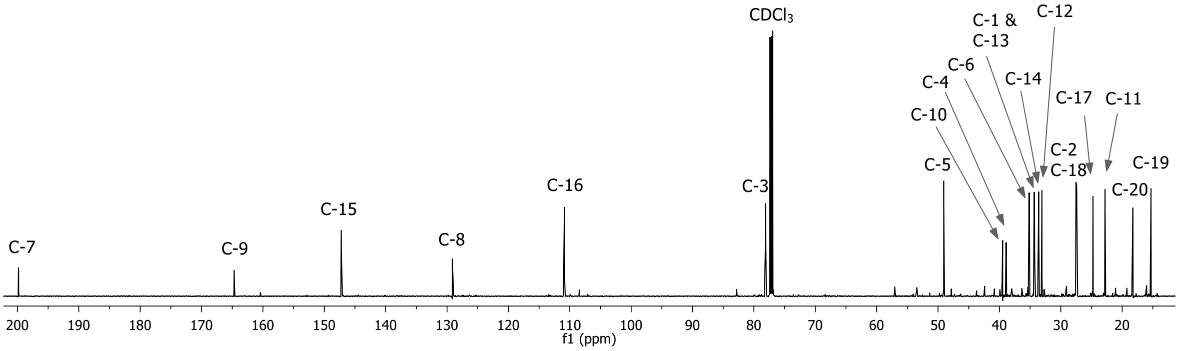

Figure S18. ^{13}C NMR spectrum of compound **3** ($CDCl_3$, 150 MHz).

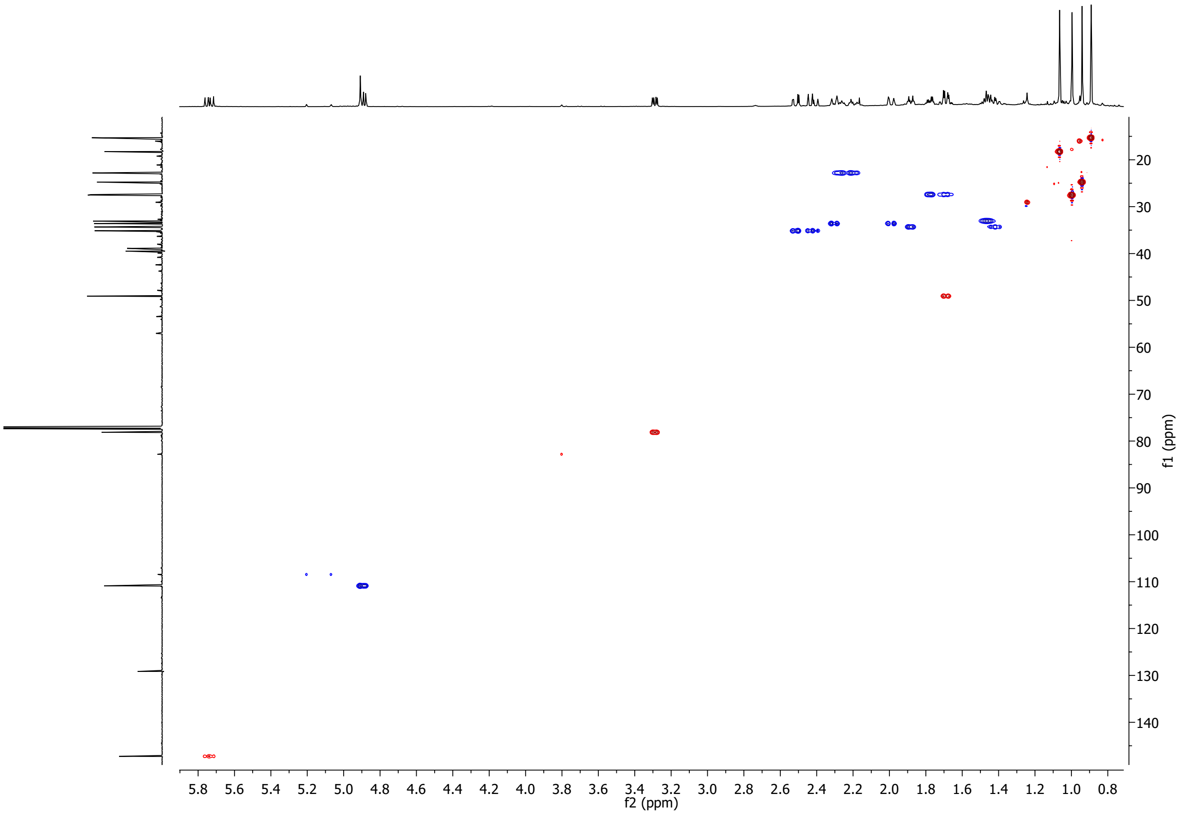

Figure S19. ^{1}H/^{13}-HSQC spectrum of compound **3** (600/150 MHz, $CDCl_3$).

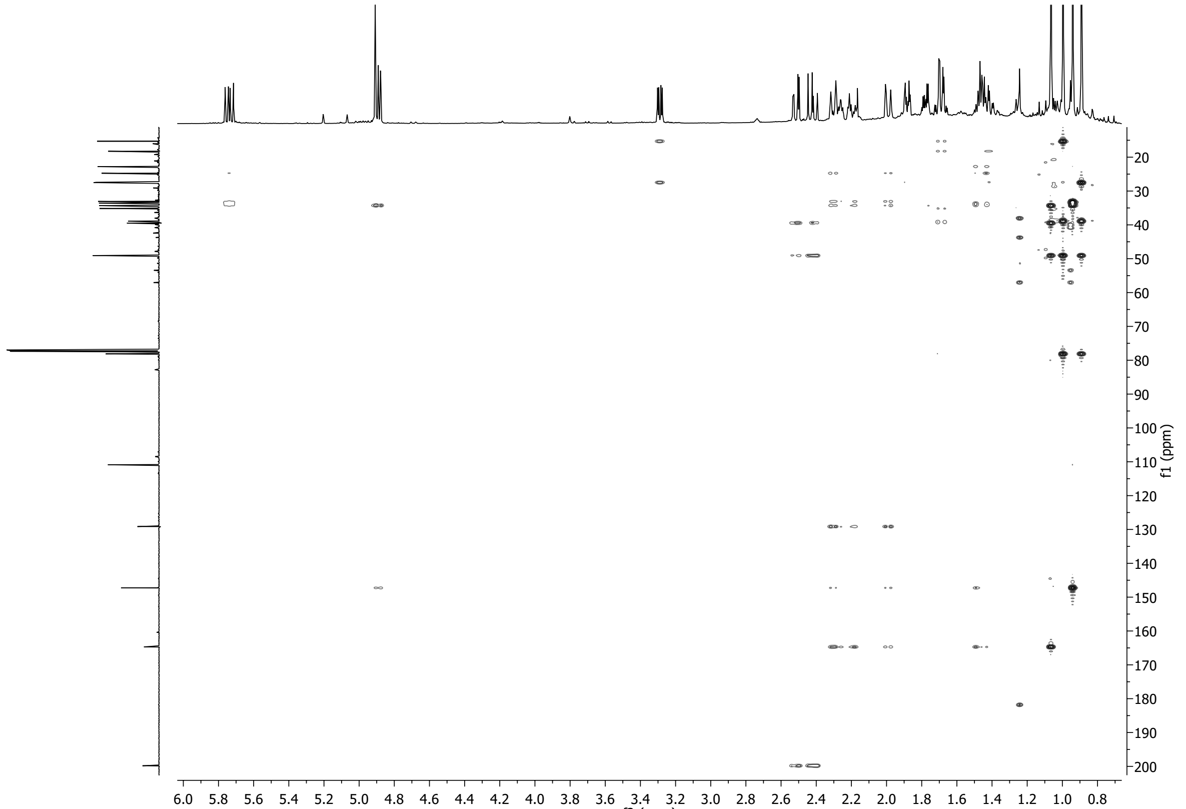

Figure S20. $^{1}H/^{13}$-HMBC spectrum of compound **3** (600/150 MHz, $CDCl_3$).

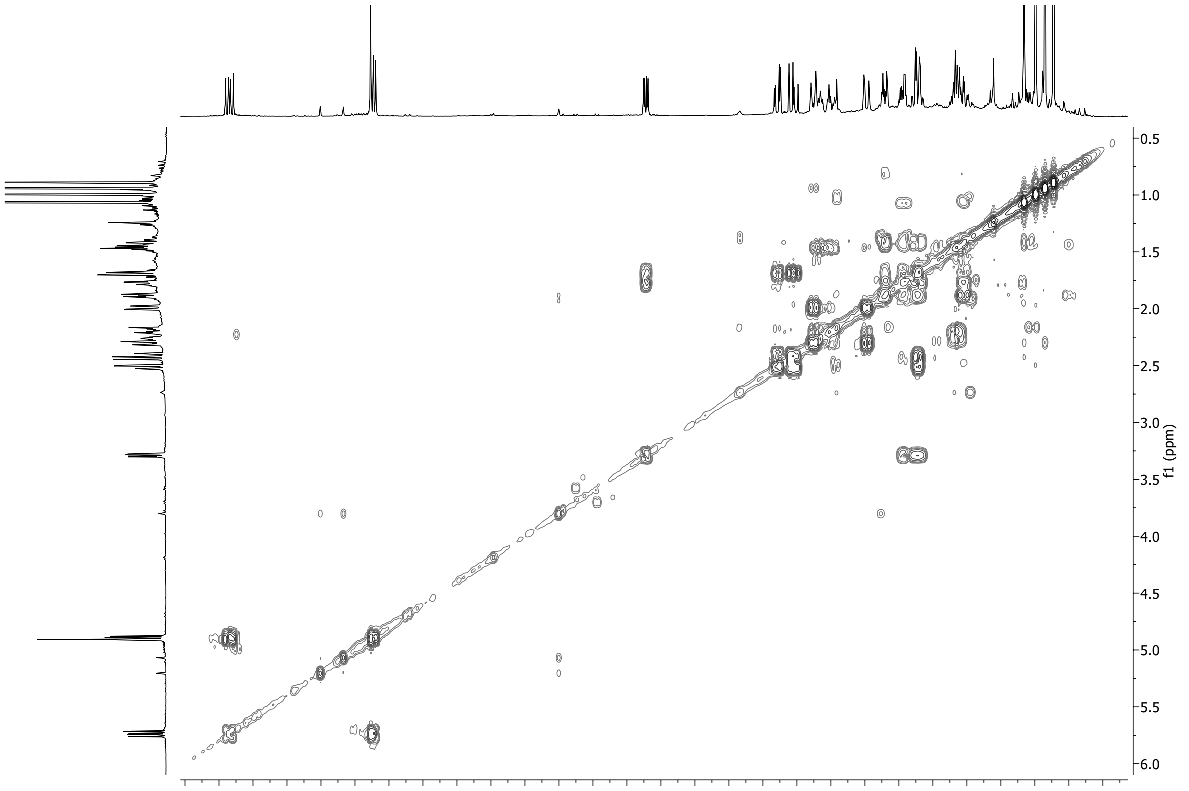

.

Figure S21. $^{1}H/^{1}H$-COSY spectrum of compound **3** (600 MHz, $CDCl_3$).

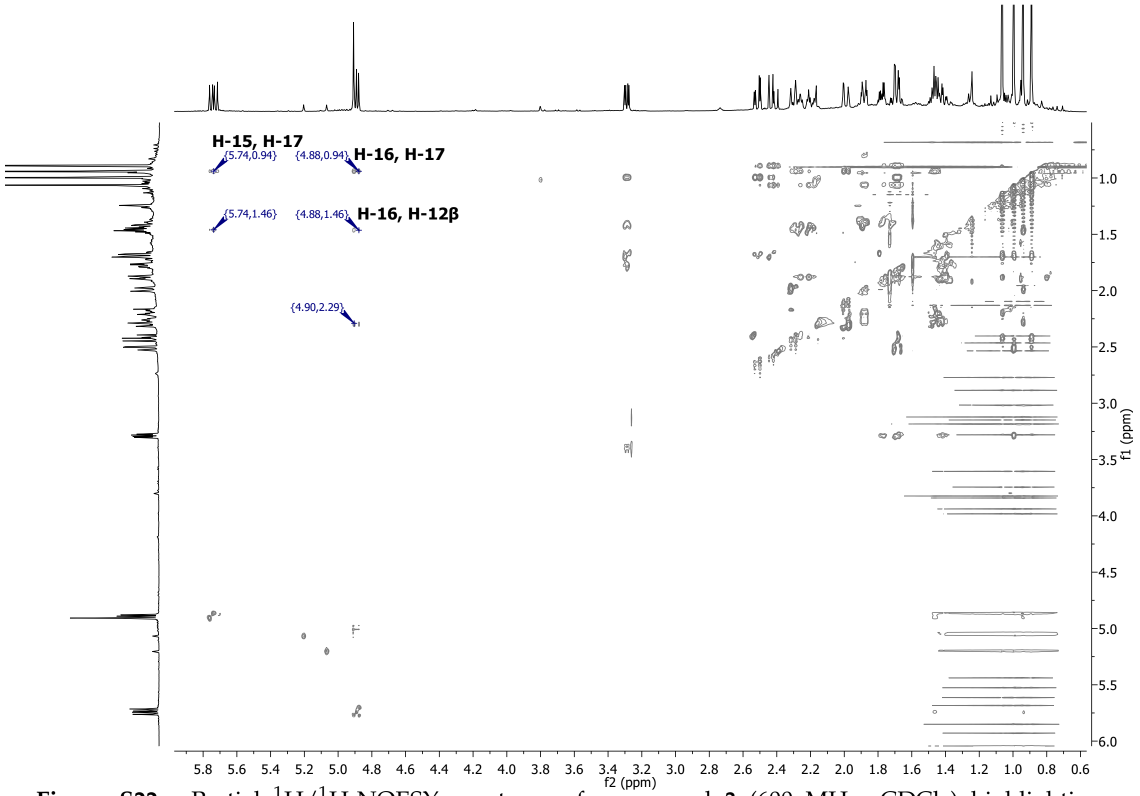

Figure S22. Partial $^1H/^1H$-NOESY spectrum of compound **3** (600 MHz, $CDCl_3$) highlighting correlations in the downfield region.

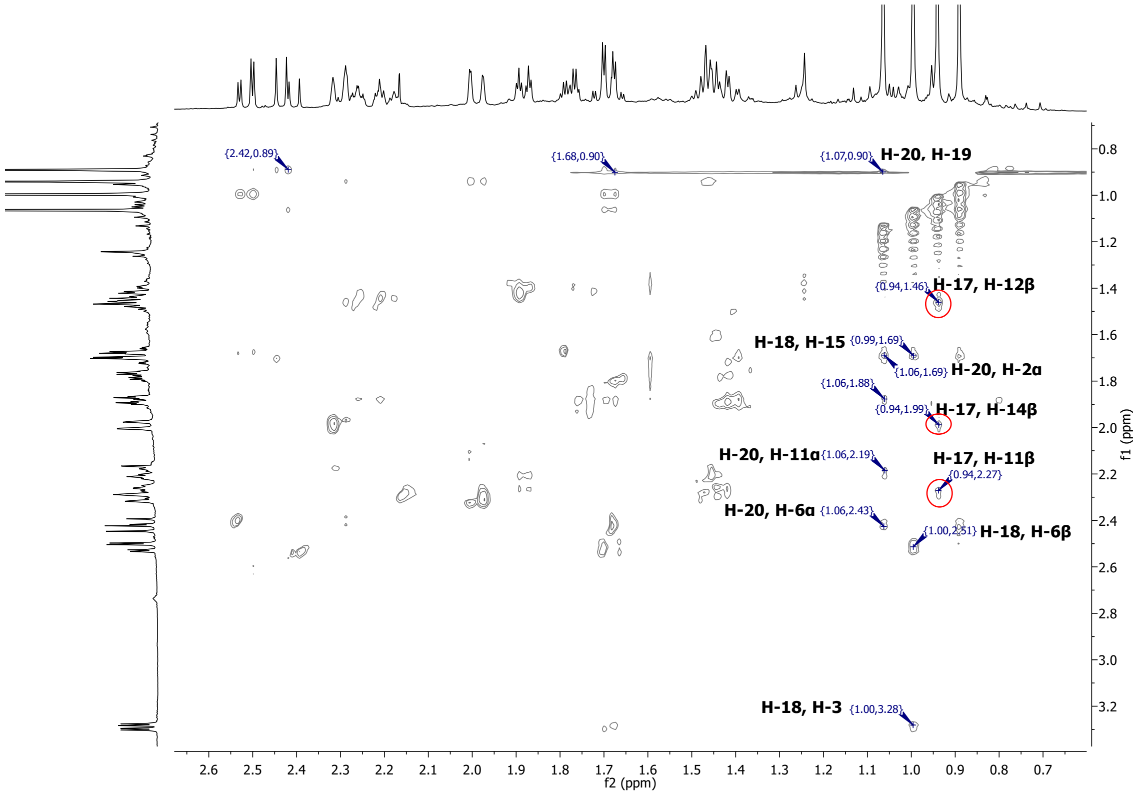

Figure S23. Partial $^1H/^1H$-NOESY spectrum of compound **3** (600 MHz, $CDCl_3$) showing correlations in the upfield region. Stereochemistry at C-13 in compound **4** is justified by the NOE effects circled in red.

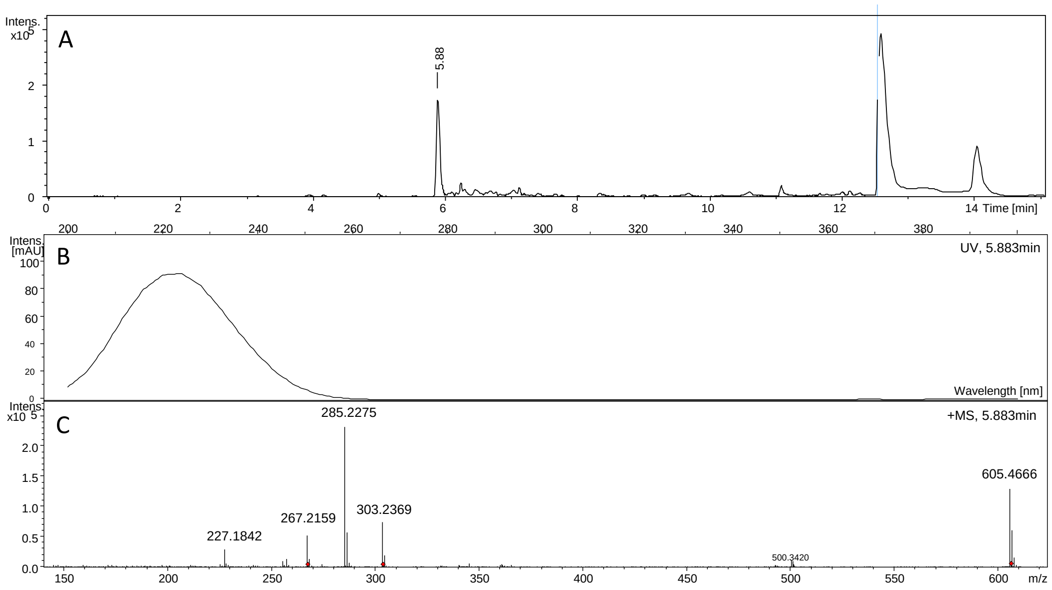

Figure S24. +ESI-QqTOF-MS chromatogram (A), UV spectrum (B), +ESI-MS (C) and +ESI-MS2 (D) spectra of compound **4**, $[M+H]^+$: 303.2369 ($C_{20}H_{31}O_2$). Internal calibrant sodium formiate: 12.6 min.

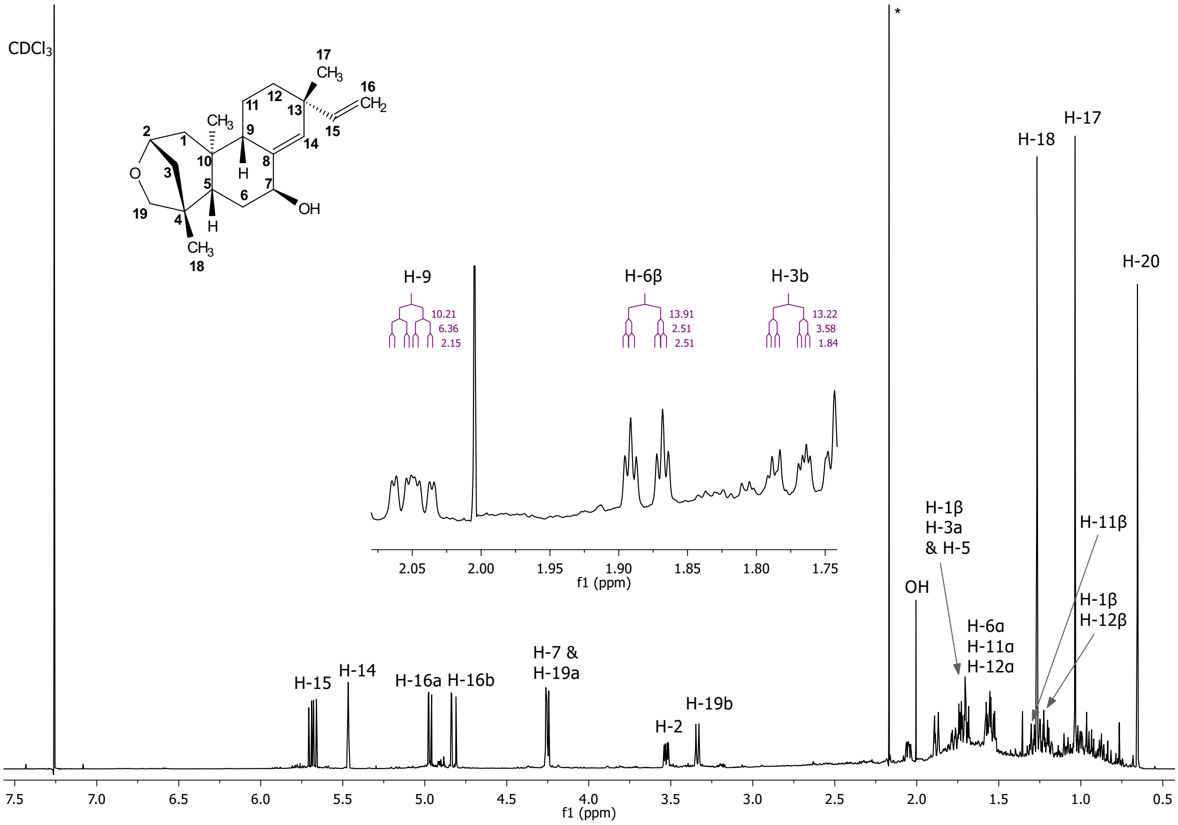

Figure S25. ^{1}H NMR spectrum of compound **4** ($CDCl_3$, 600 MHz). *-CH_3 protons from acetone.

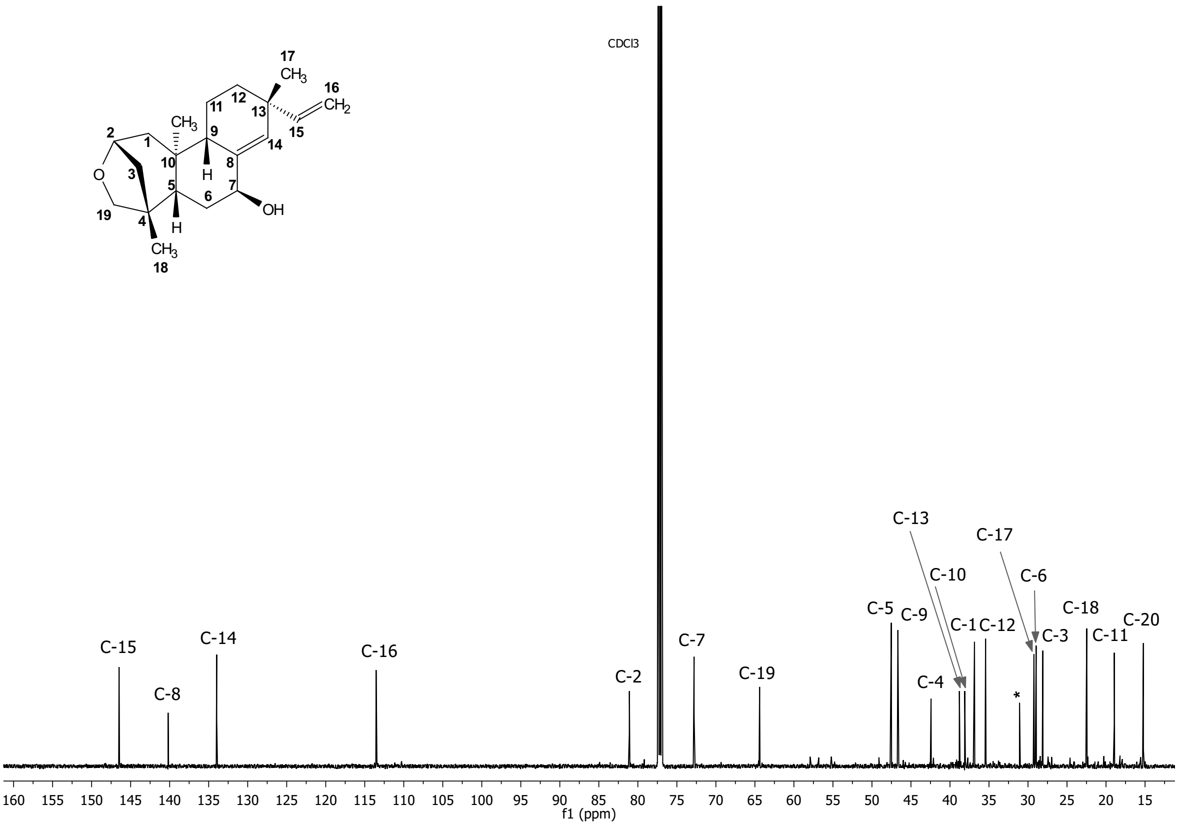

Figure S26. ^{13}C NMR spectrum of compound **4** ($CDCl_3$, 150 MHz). *-CH_3 from acetone.

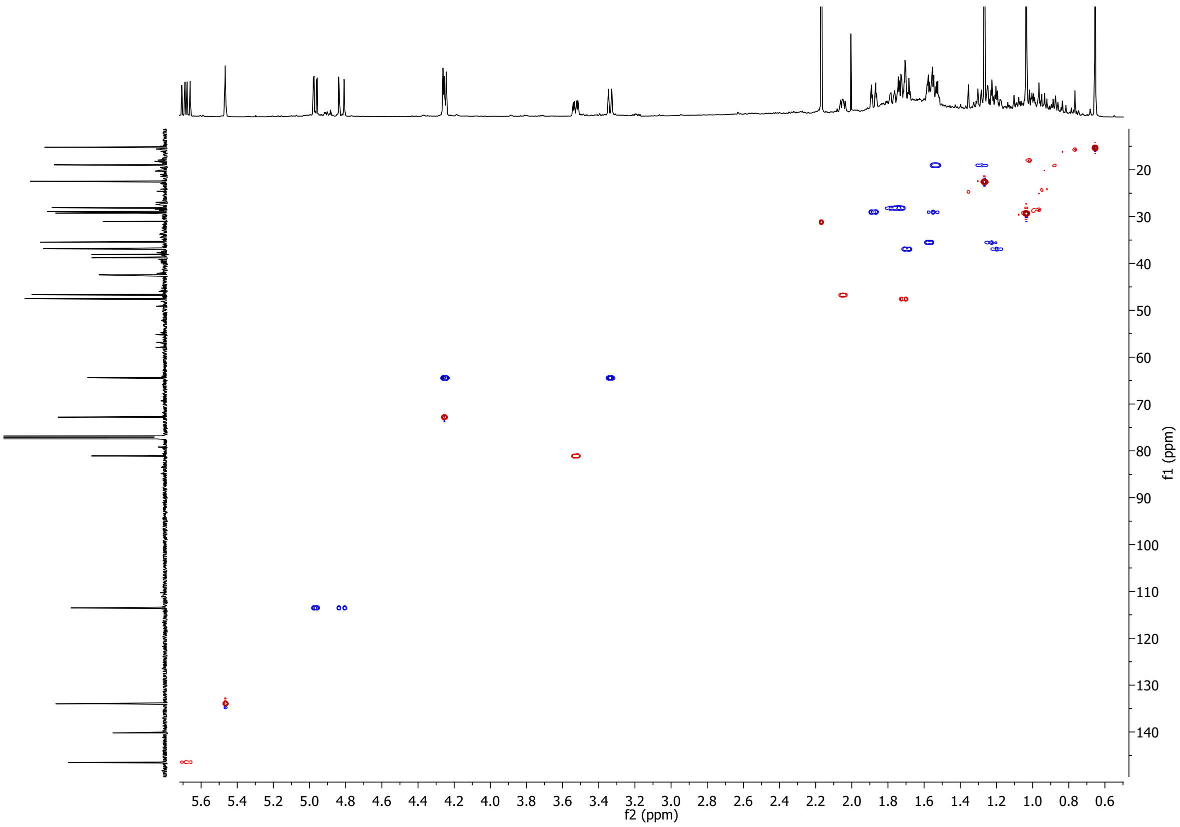

Figure S27. $^{1}H/^{13}C$-HSQC spectrum of compound **4** (600/150 MHz, $CDCl_3$).

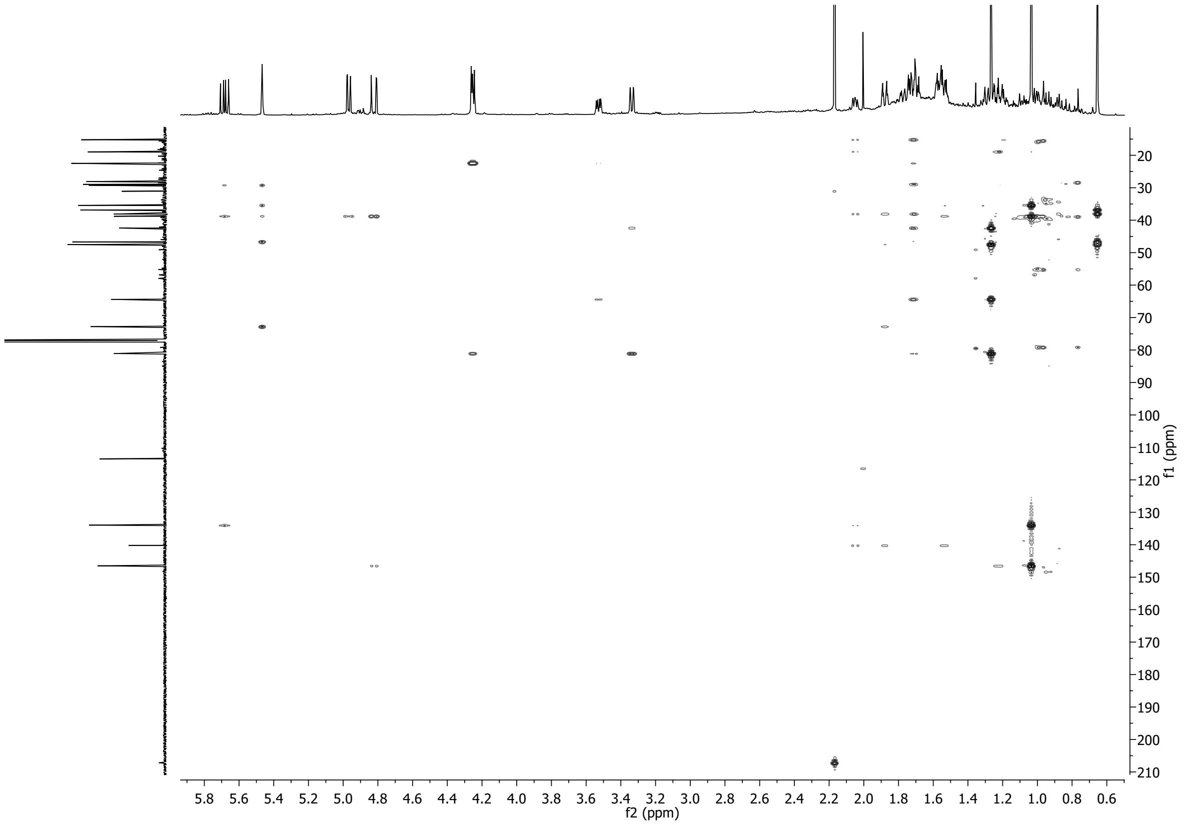

Figure S28. $^{1}H/^{13}C$-HMBC spectrum of compound **4** (600/150 MHz, $CDCl_3$).

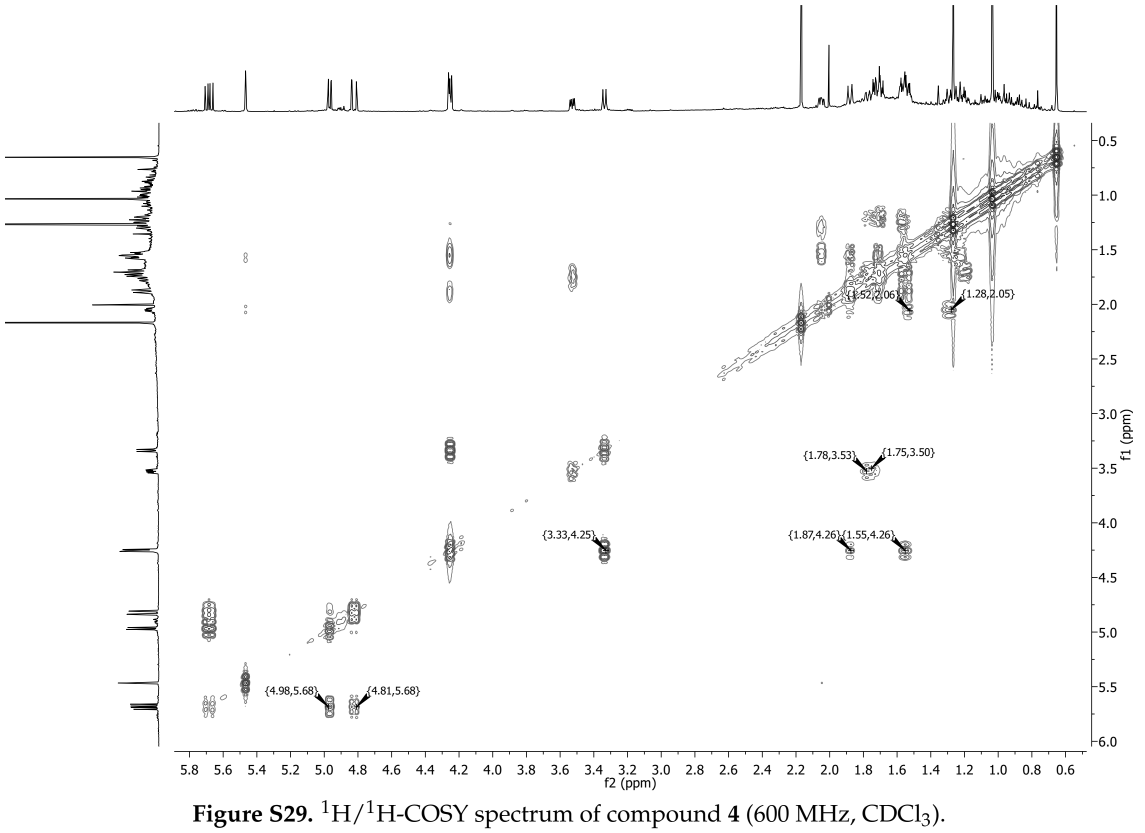

Figure S29. $^{1}H/^{1}H$-COSY spectrum of compound **4** (600 MHz, $CDCl_3$).

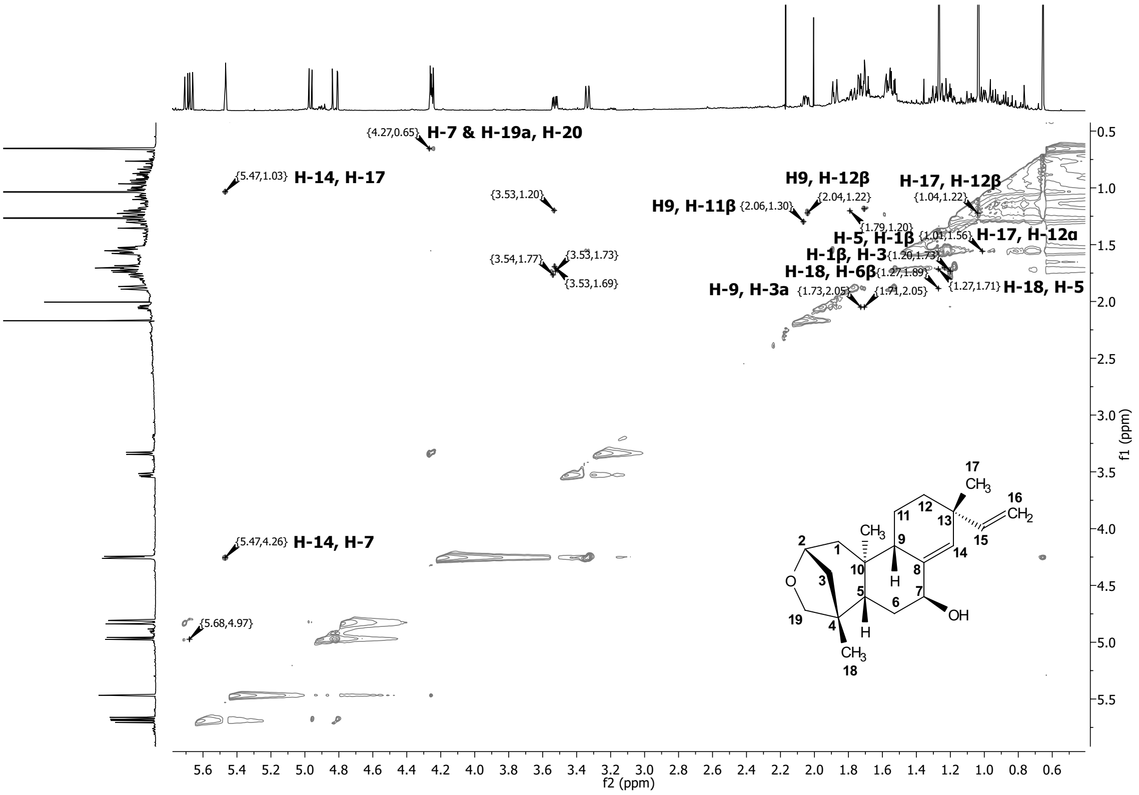

Figure S30. $^1H/^1H$-NOESY spectrum of compound **4** (600 MHz, $CDCl_3$).
