# Supplementary figures and images for: ent-Pimarane and ent-Kaurane Diterpenes from Aldama discolor (Asteraceae) and Their Antiprotozoal Activity
Source: Molecules. 2016 Sep 15;21(9):1237. doi: 10.3390/molecules21091237 (PMC6274167; doi:10.3390/molecules21091237)

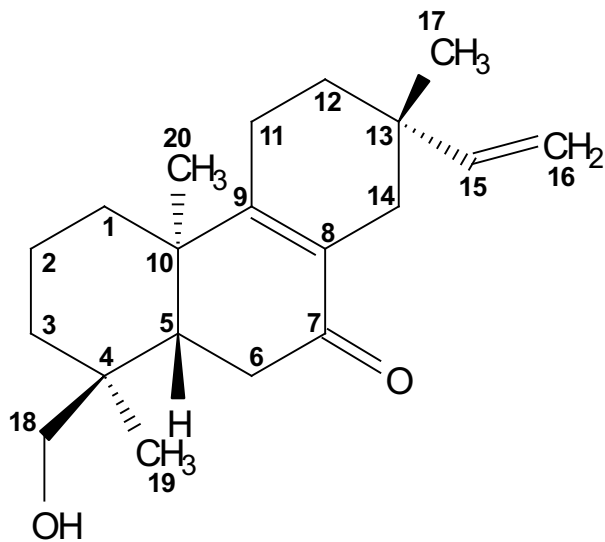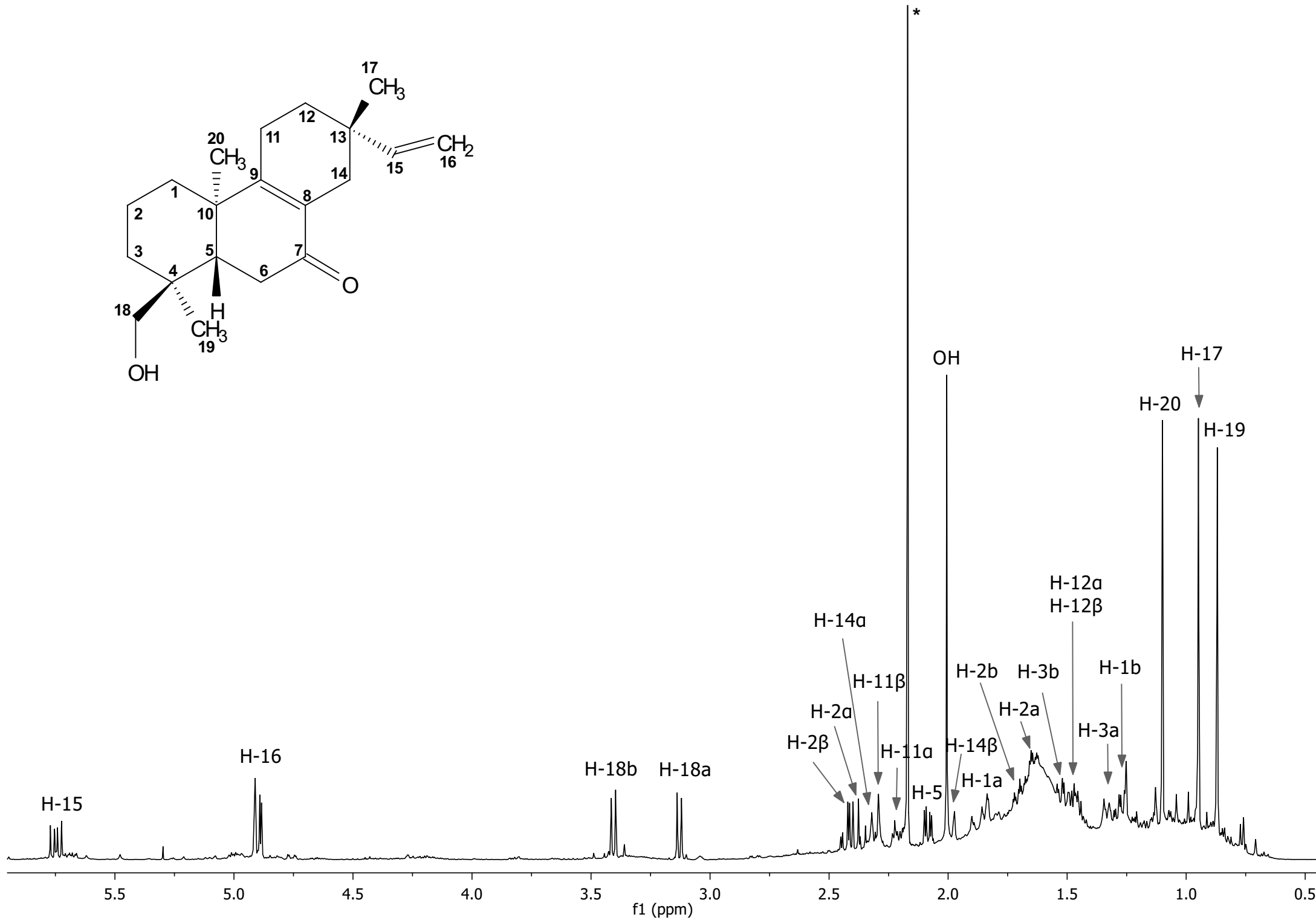

Supplement: Supplementary file 2 [file molecules-21-01237-s002.zip › Fig_1H_NMR_S_11.pdf]

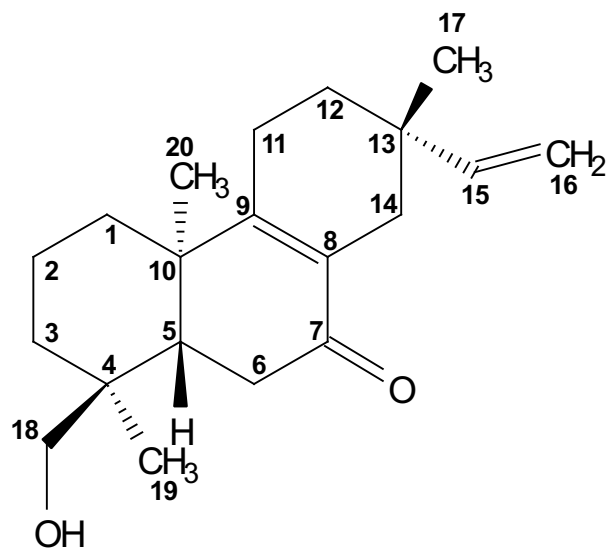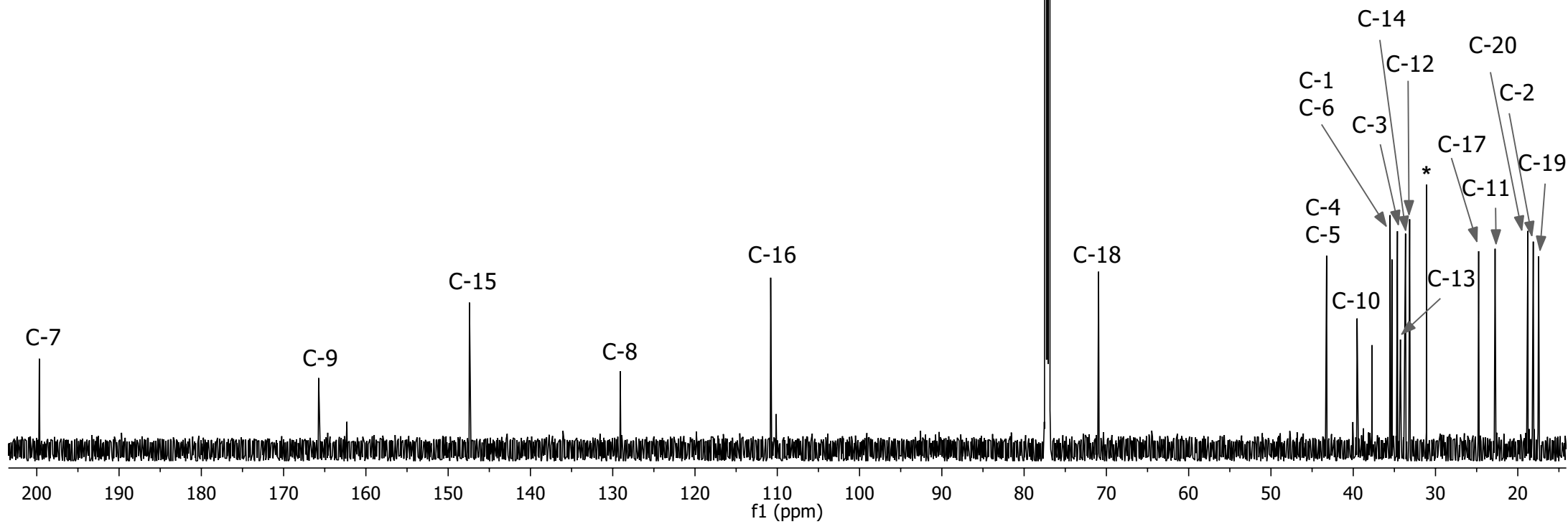

Supplement: Supplementary file 2 [file molecules-21-01237-s002.zip › Fig_13C_NMR_S_11.pdf]

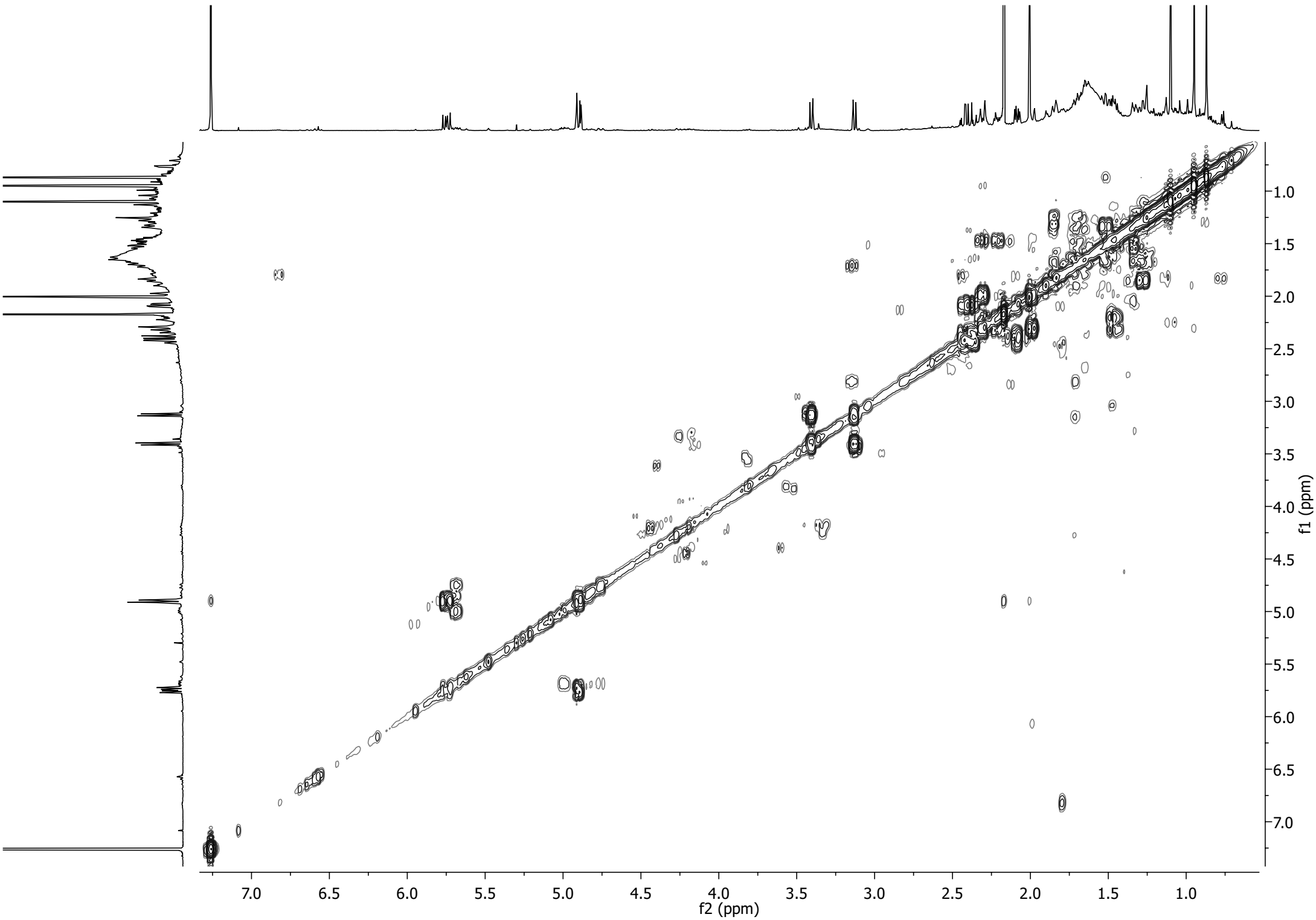

Supplement: Supplementary file 2 [file molecules-21-01237-s002.zip › Fig_COSY_11.pdf]

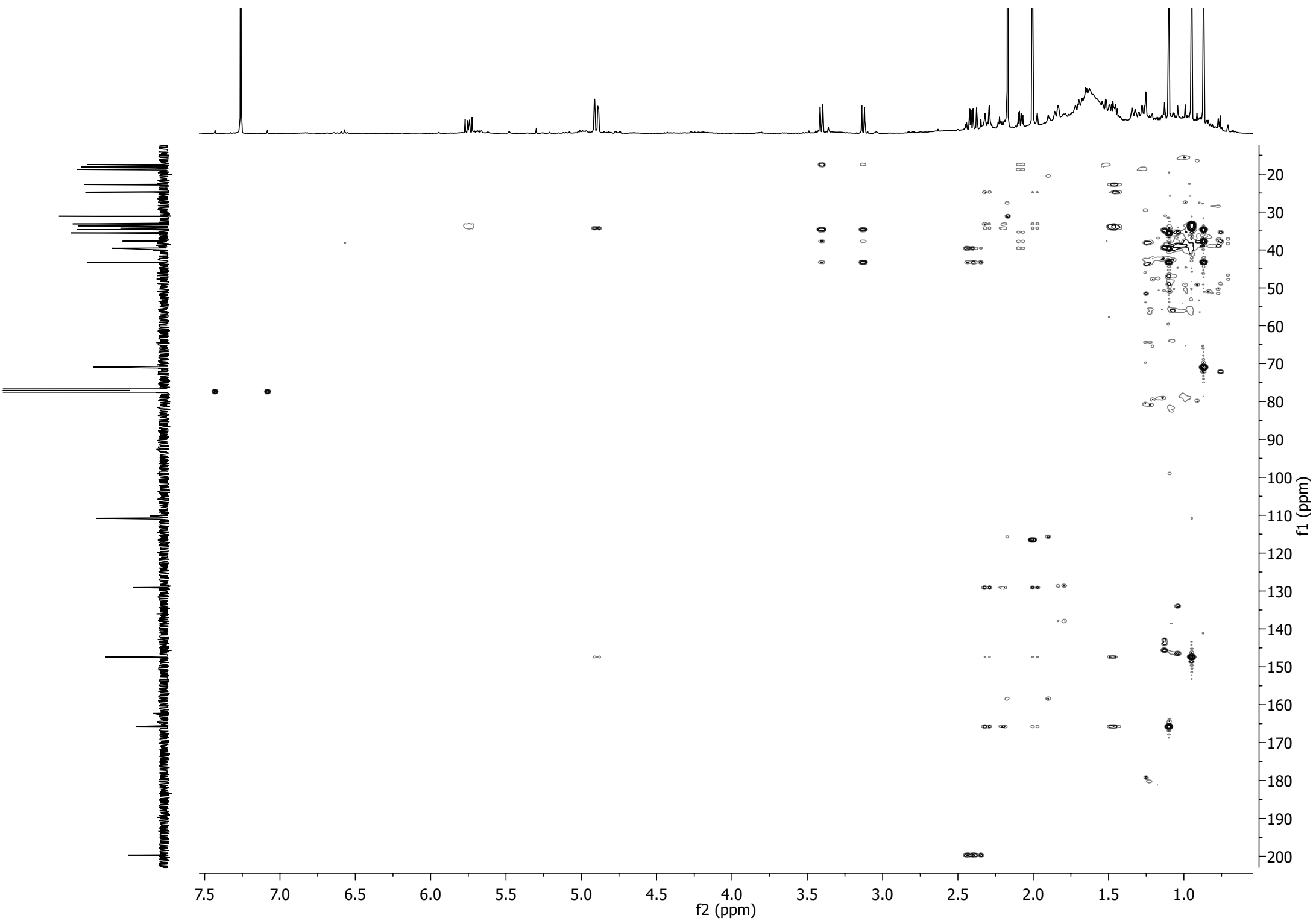

Supplement: Supplementary file 2 [file molecules-21-01237-s002.zip › Fig_HMBC_11.pdf]

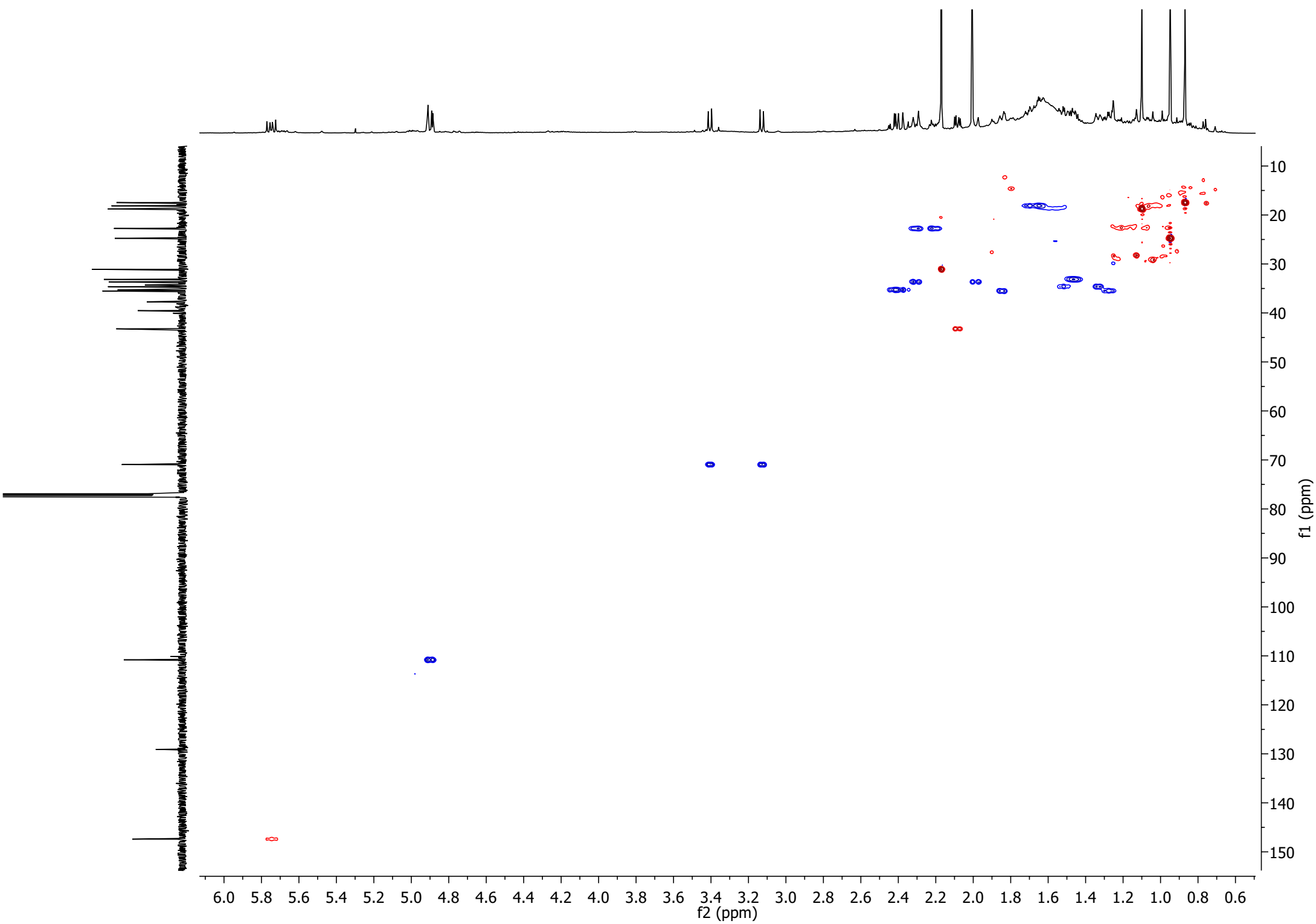

Supplement: Supplementary file 2 [file molecules-21-01237-s002.zip › Fig_HSQC_11.pdf]

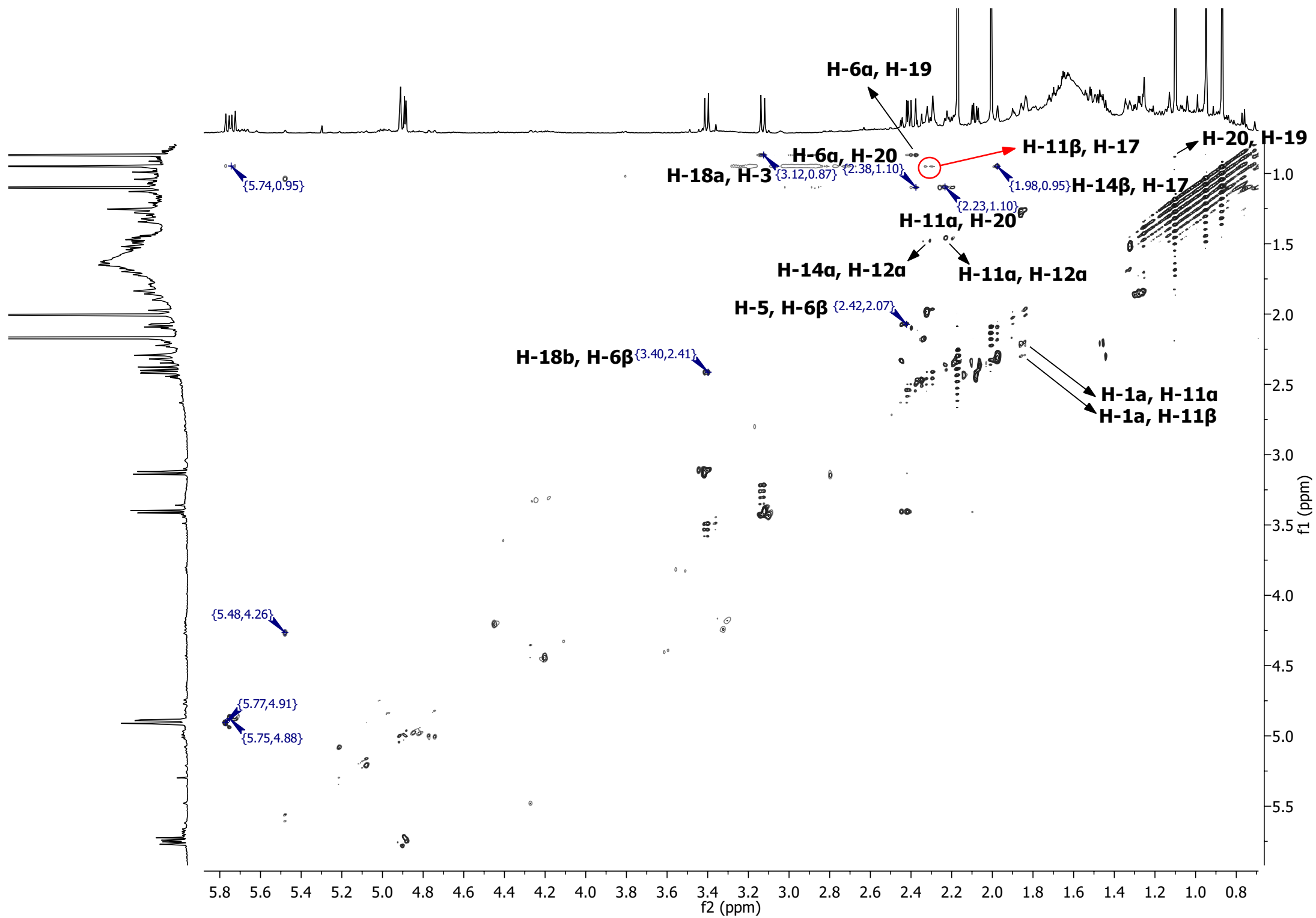

Supplement: Supplementary file 2 [file molecules-21-01237-s002.zip › Fig_NOESY_11.pdf]

CDCl<sub>3</sub>

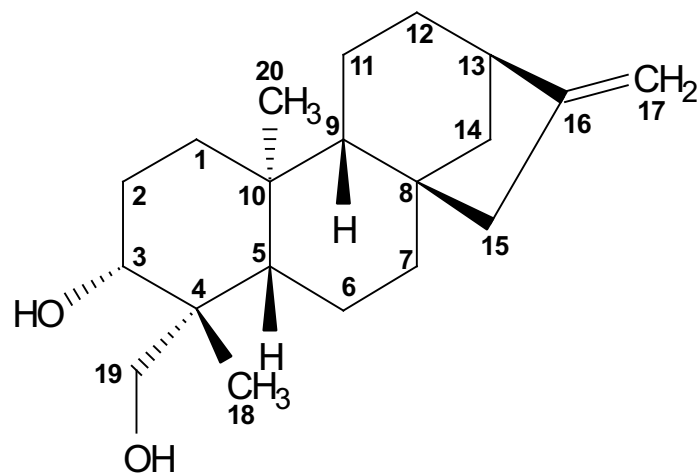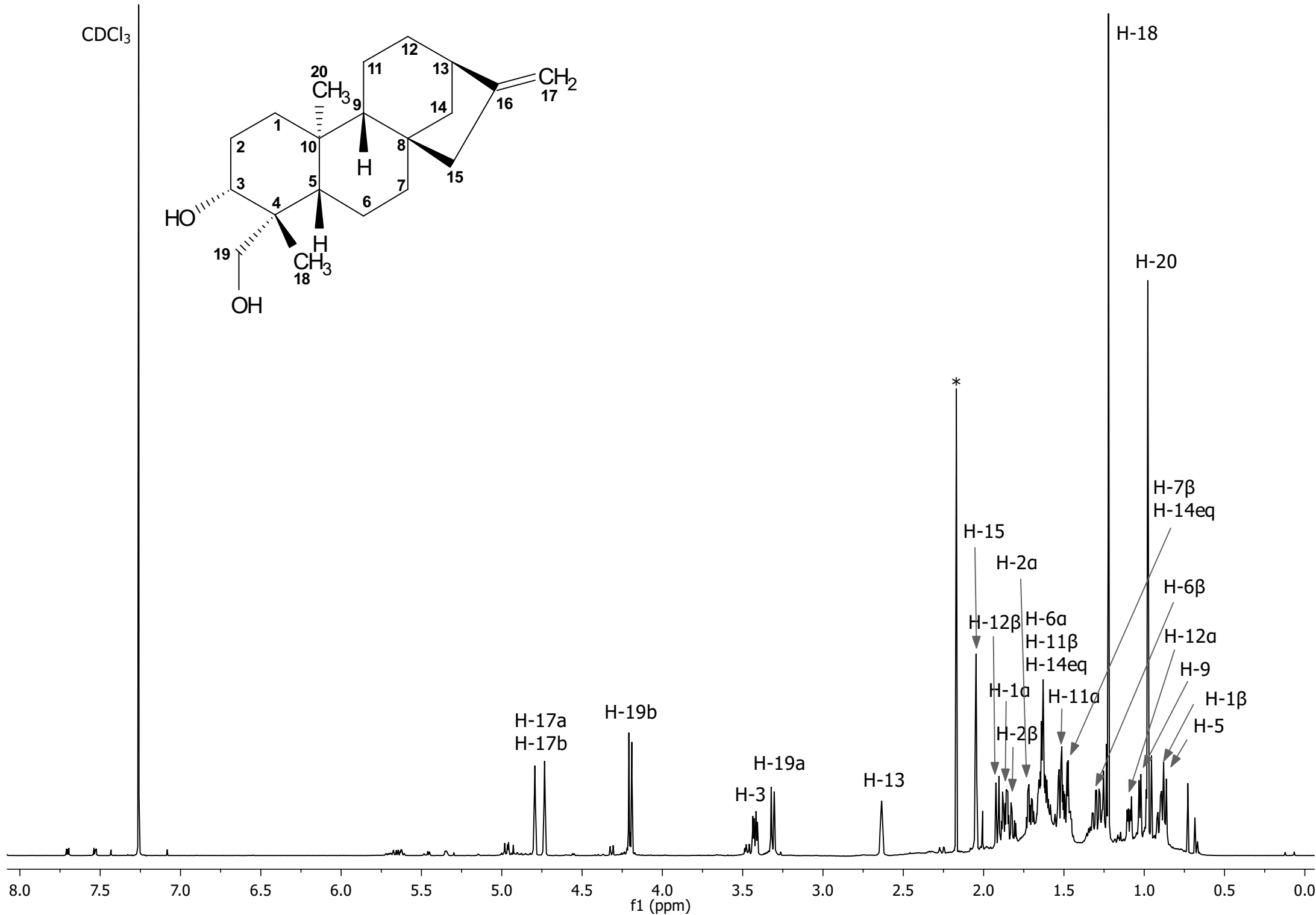

Supplement: Supplementary file 2 [file molecules-21-01237-s002.zip › Fig1_1H_NMR_S_12.pdf]

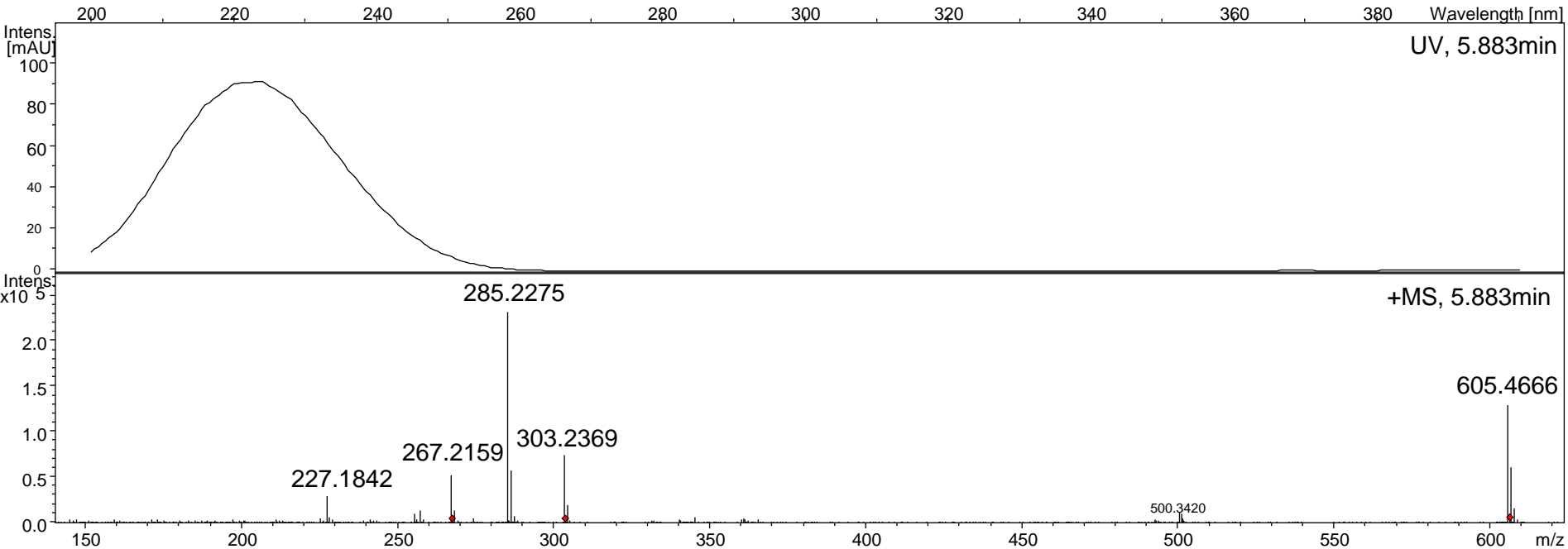

Supplement: Supplementary file 2 [file molecules-21-01237-s002.zip › Fig1_MS_S_13.pdf]

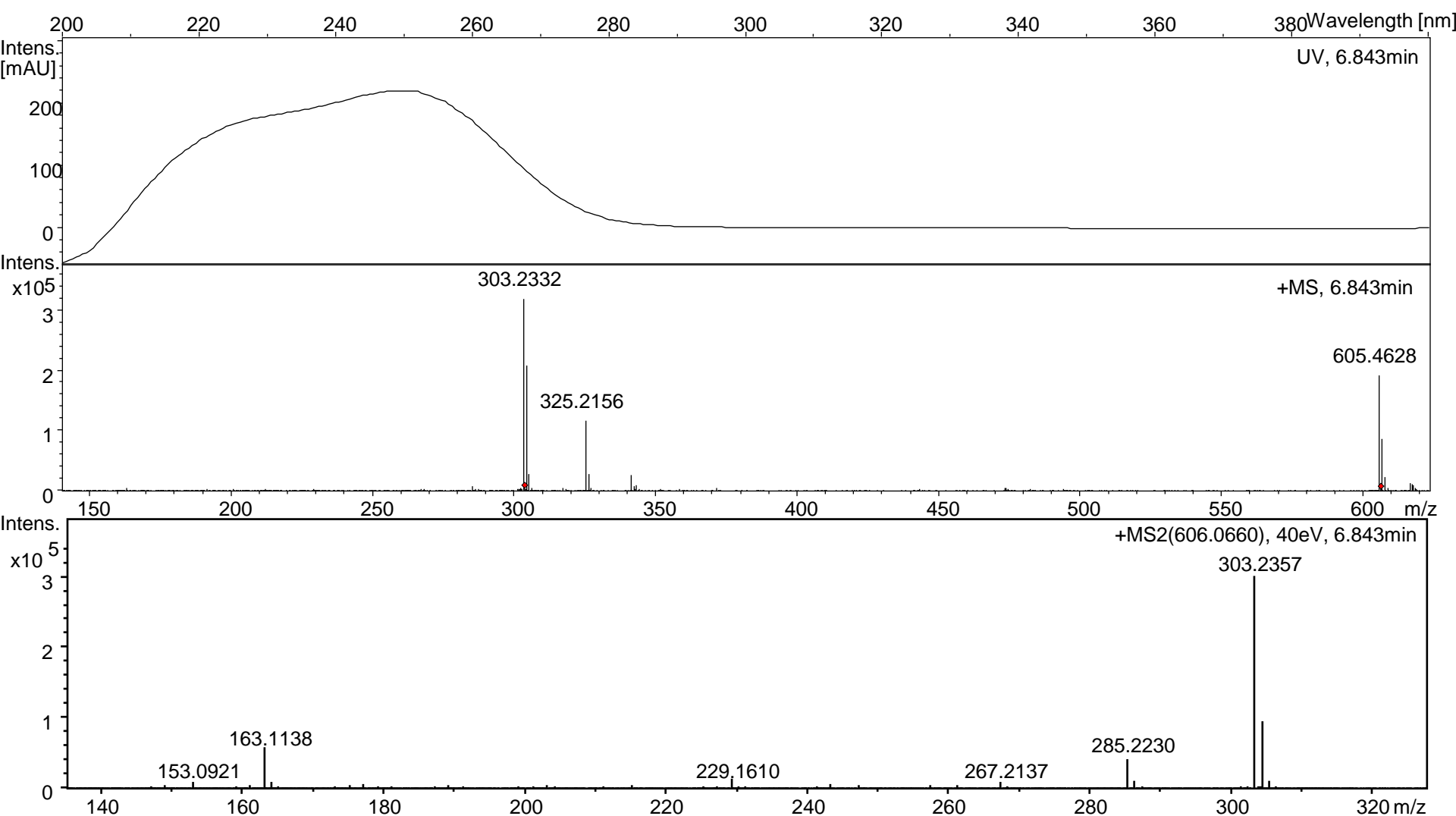

Supplement: Supplementary file 2 [file molecules-21-01237-s002.zip › Fig2_MS_S_14.pdf]

**A**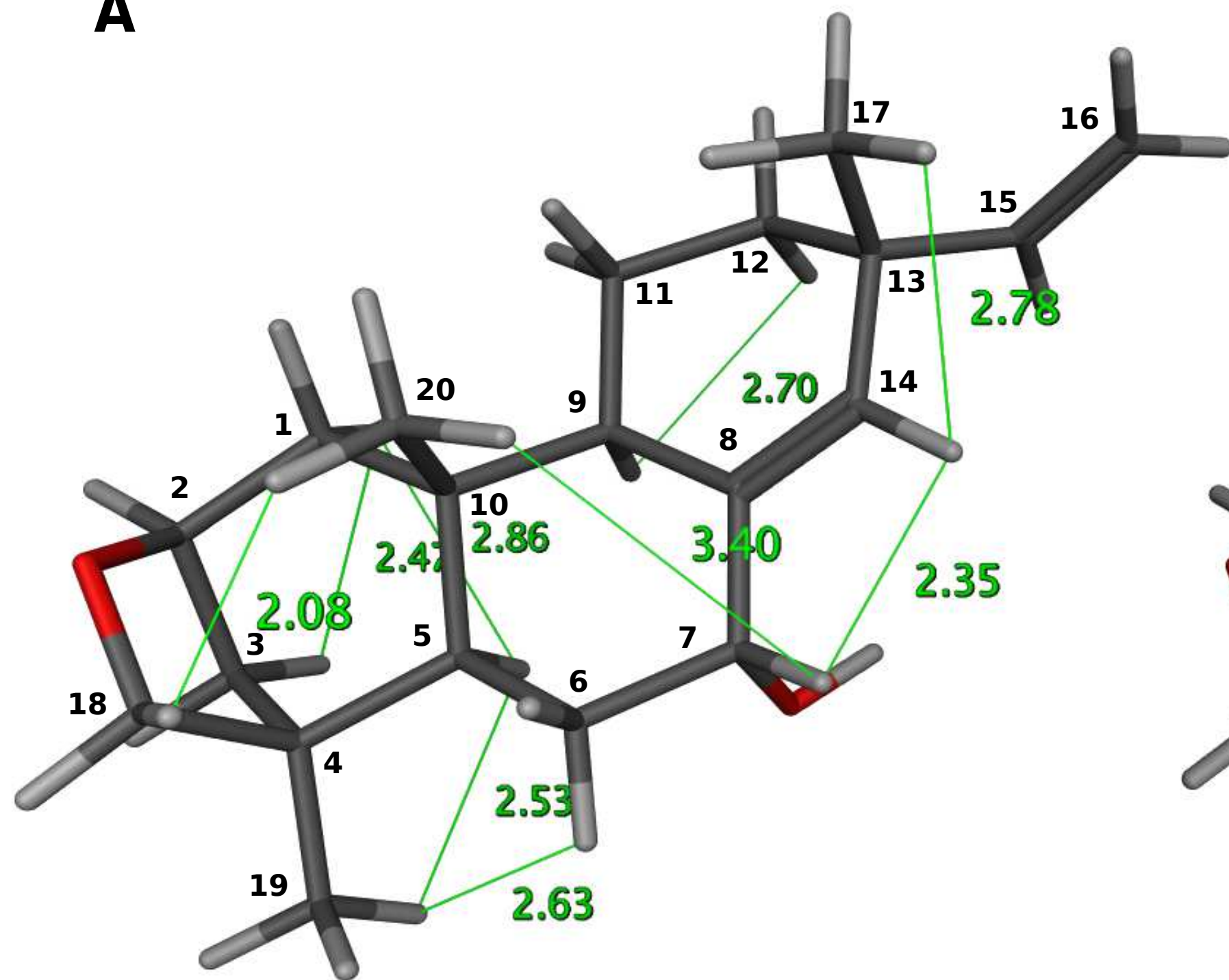**B**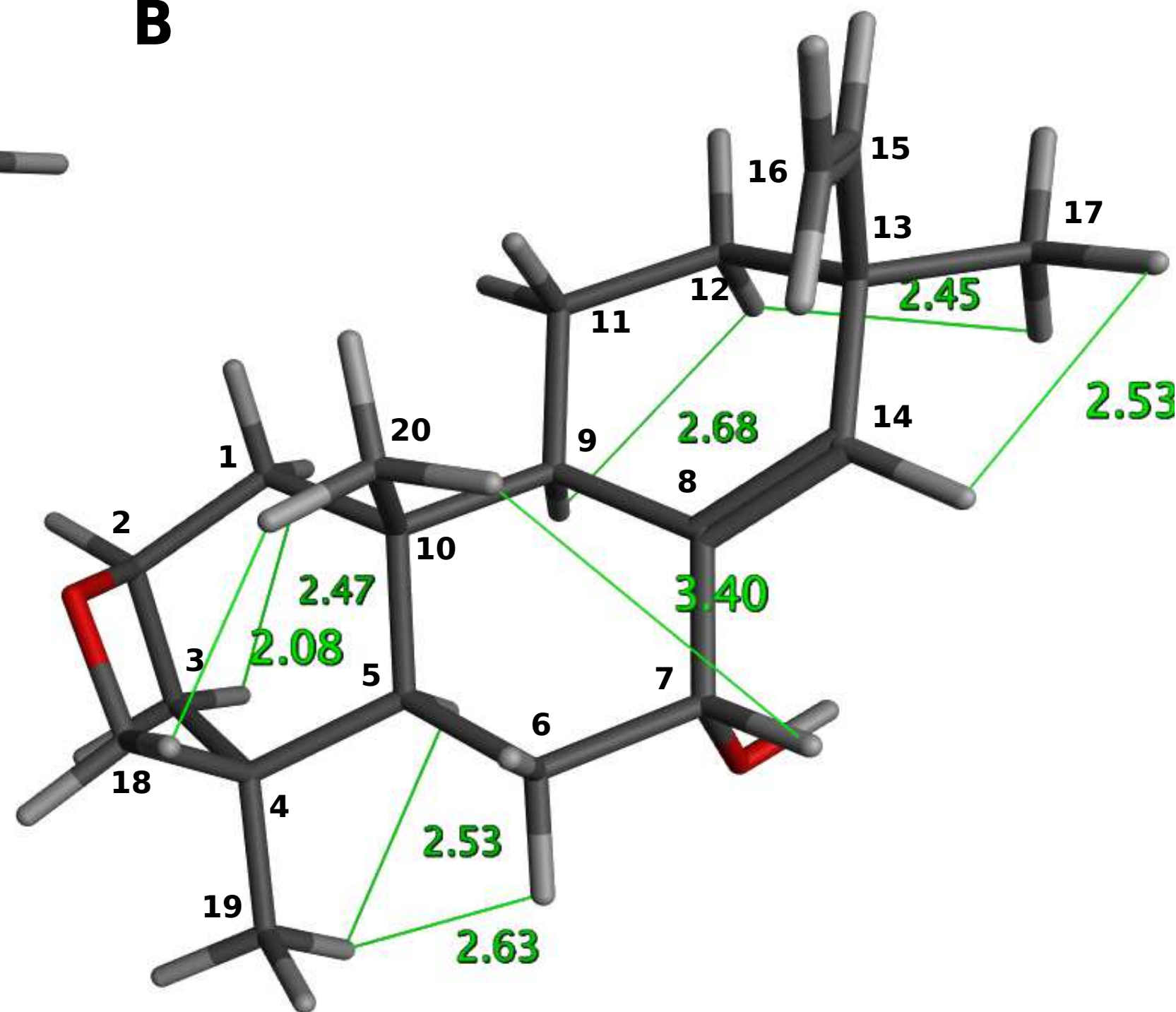

Supplement: Supplementary file 2 [file molecules-21-01237-s002.zip › Fig2_NOE_13-eps-converted-to.pdf]

CDCl<sub>3</sub>

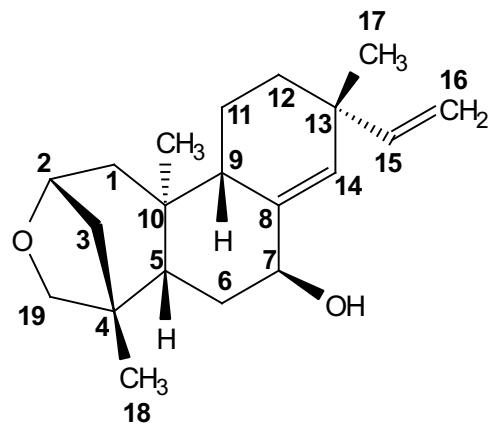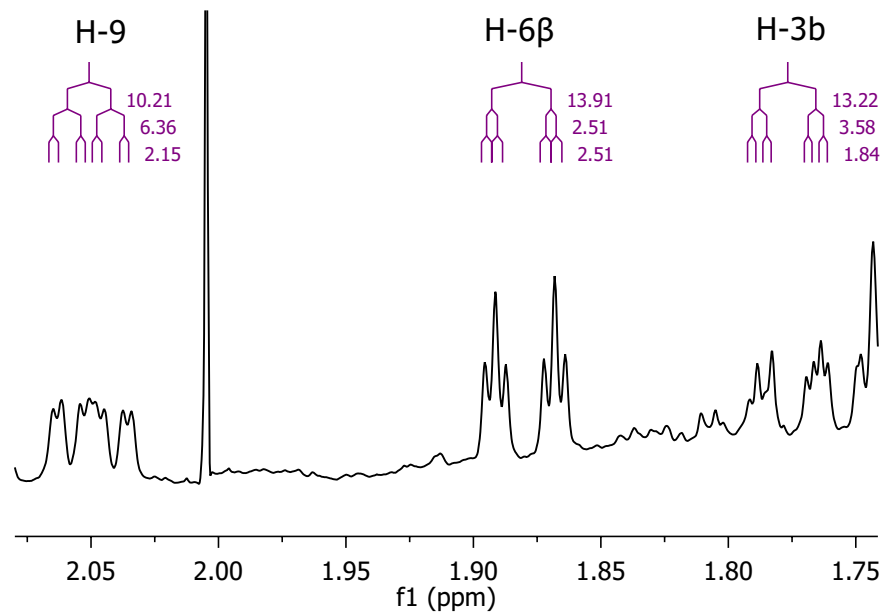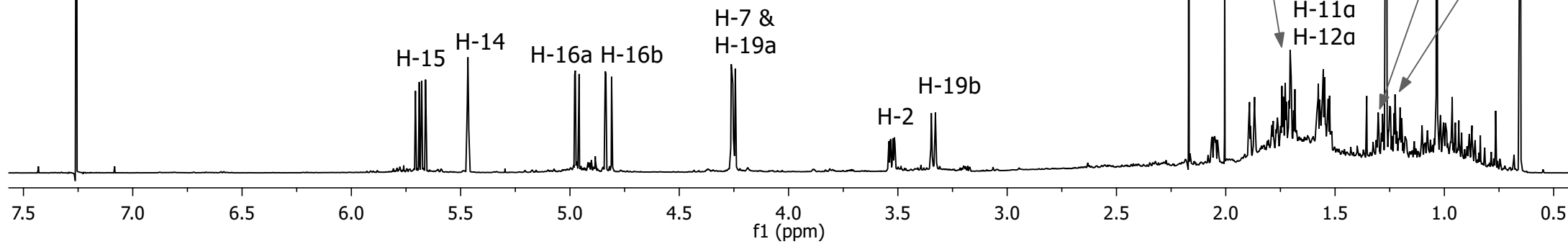

Supplement: Supplementary file 2 [file molecules-21-01237-s002.zip › Fig3_1H_NMR_S_13.pdf]

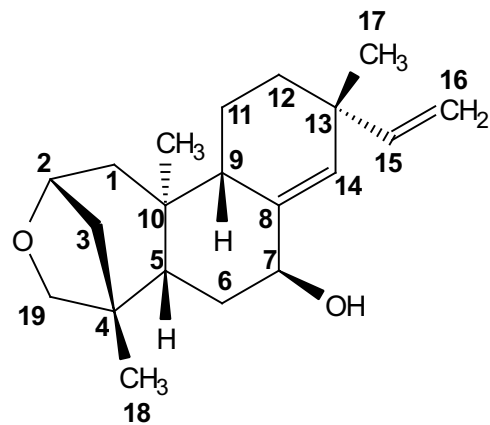

CDCl<sub>3</sub>

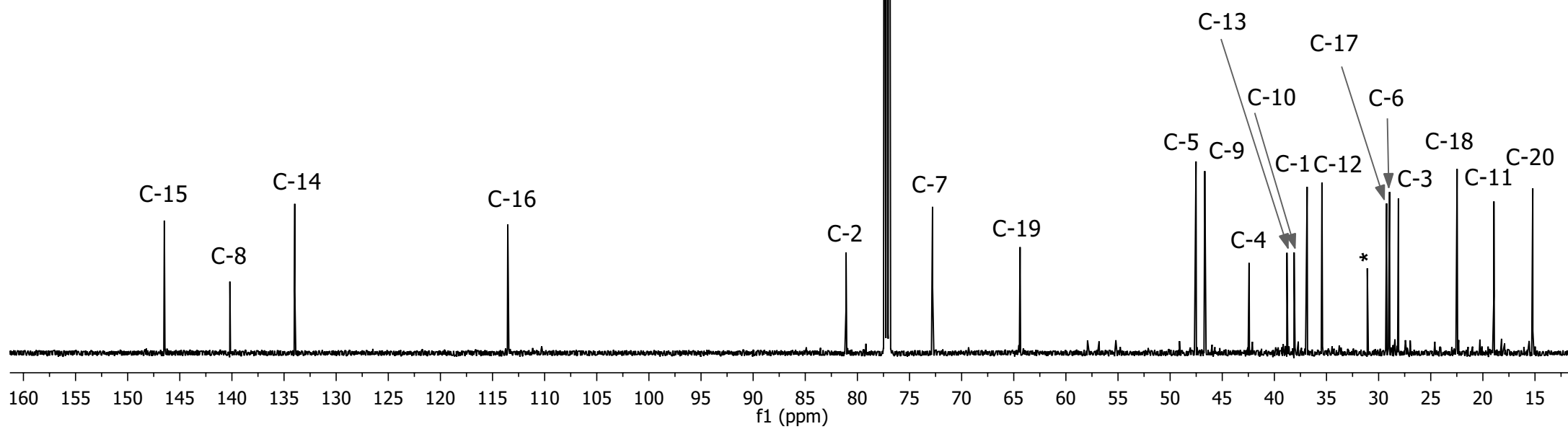

Supplement: Supplementary file 2 [file molecules-21-01237-s002.zip › Fig4_13C_NMR_S_13.pdf]

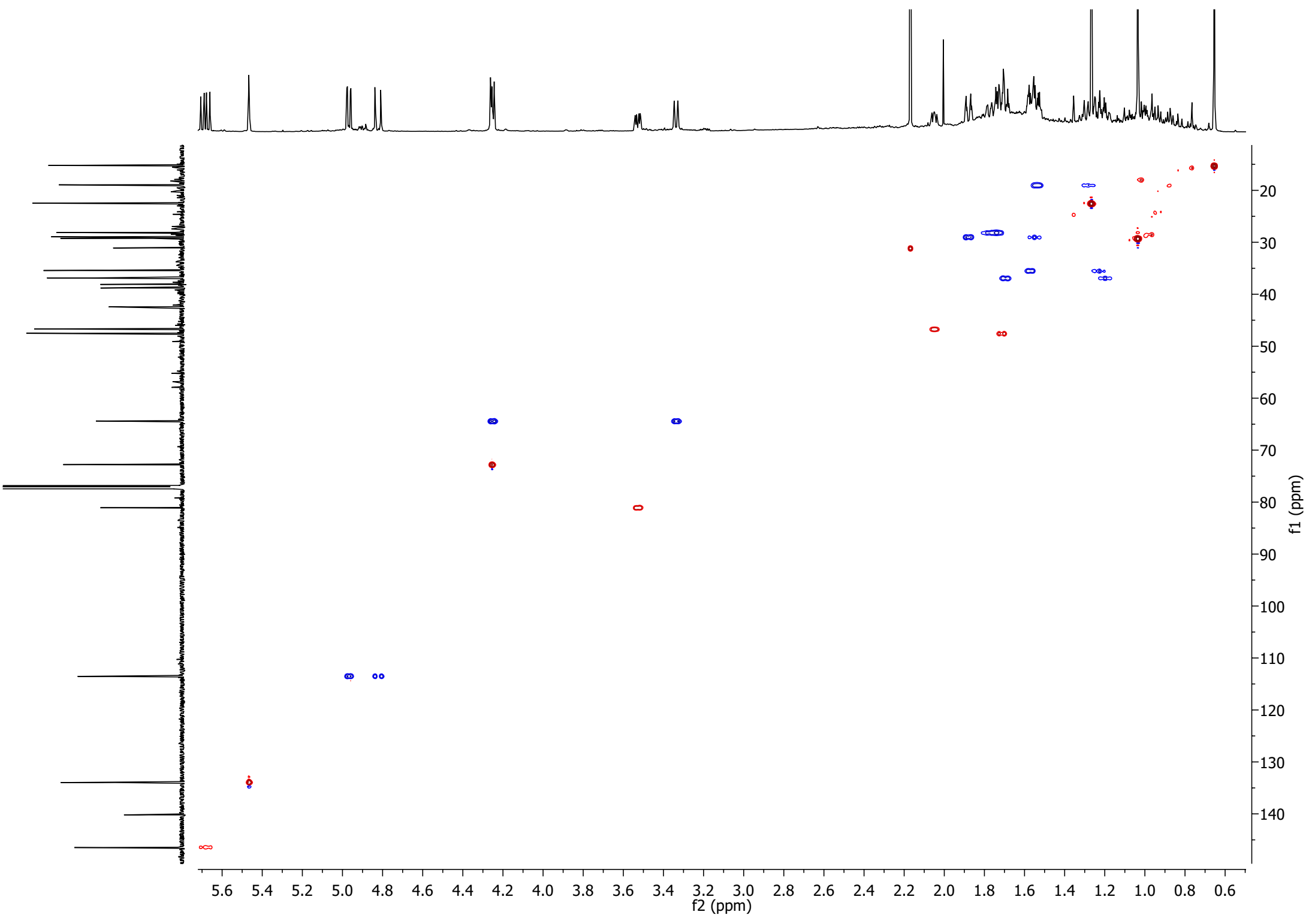

Supplement: Supplementary file 2 [file molecules-21-01237-s002.zip › Fig5_HSQC_S_13.pdf]

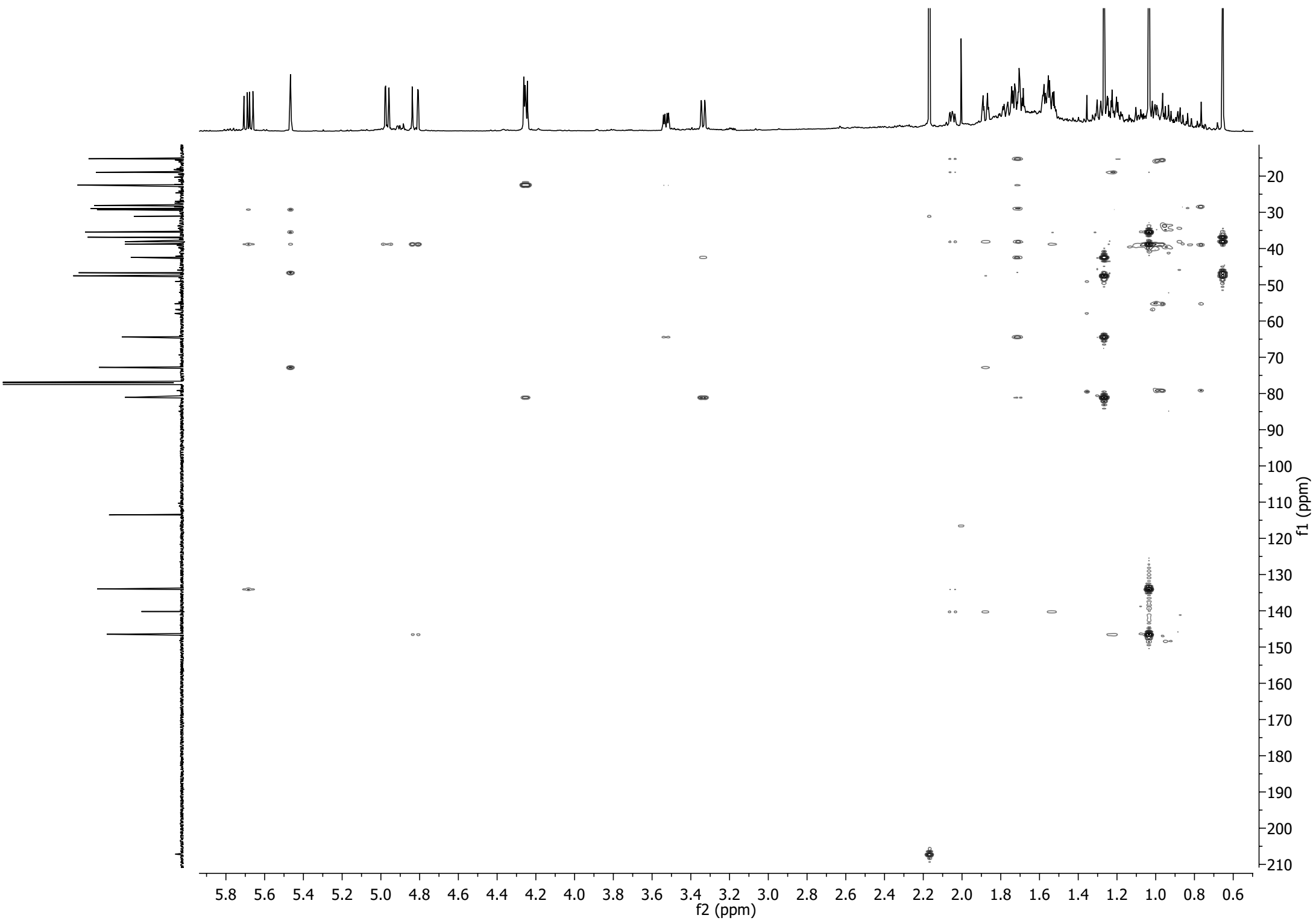

Supplement: Supplementary file 2 [file molecules-21-01237-s002.zip › Fig6_HMBC_S_13.pdf]

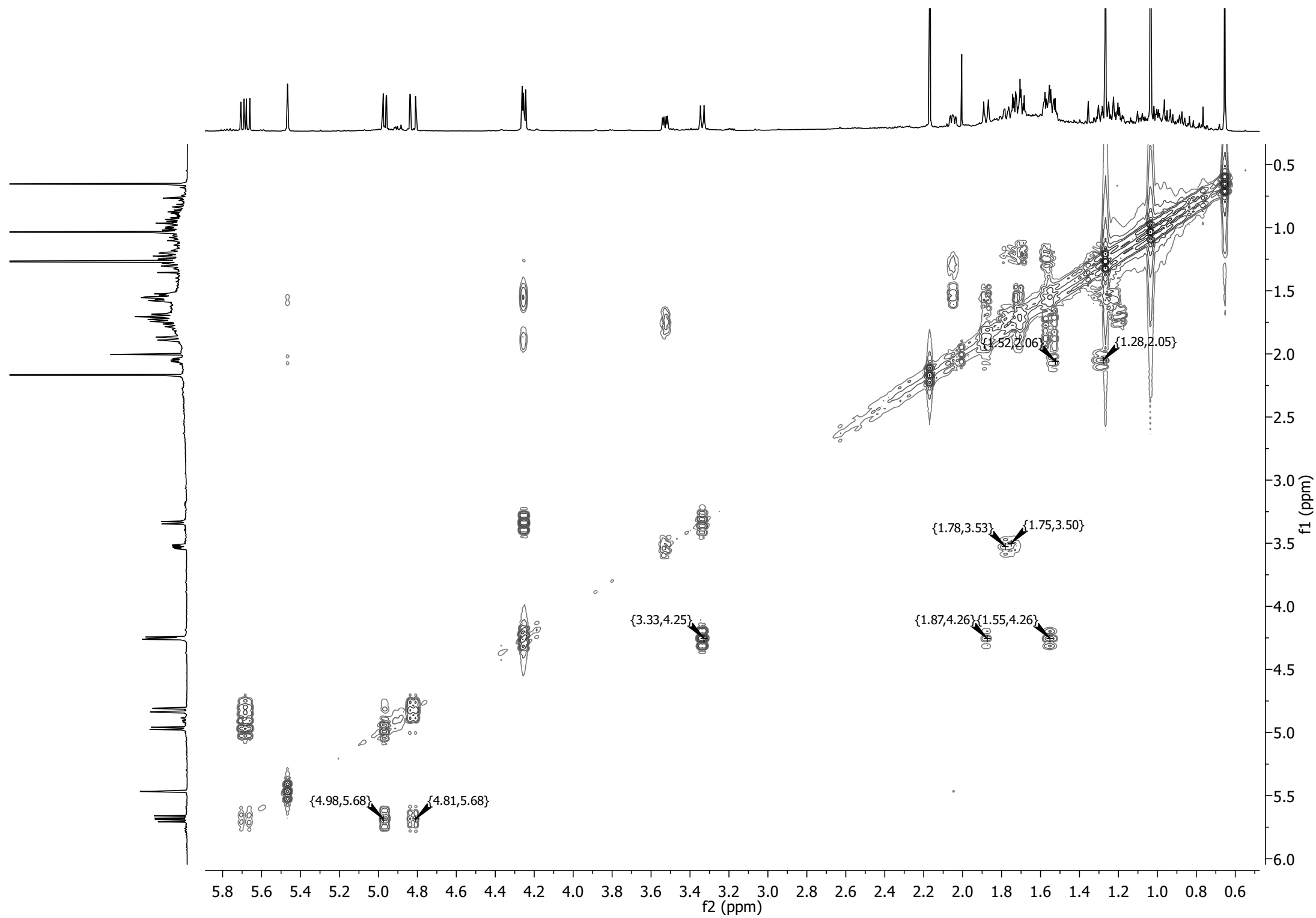

Supplement: Supplementary file 2 [file molecules-21-01237-s002.zip › Fig7_COSY_S_13.pdf]

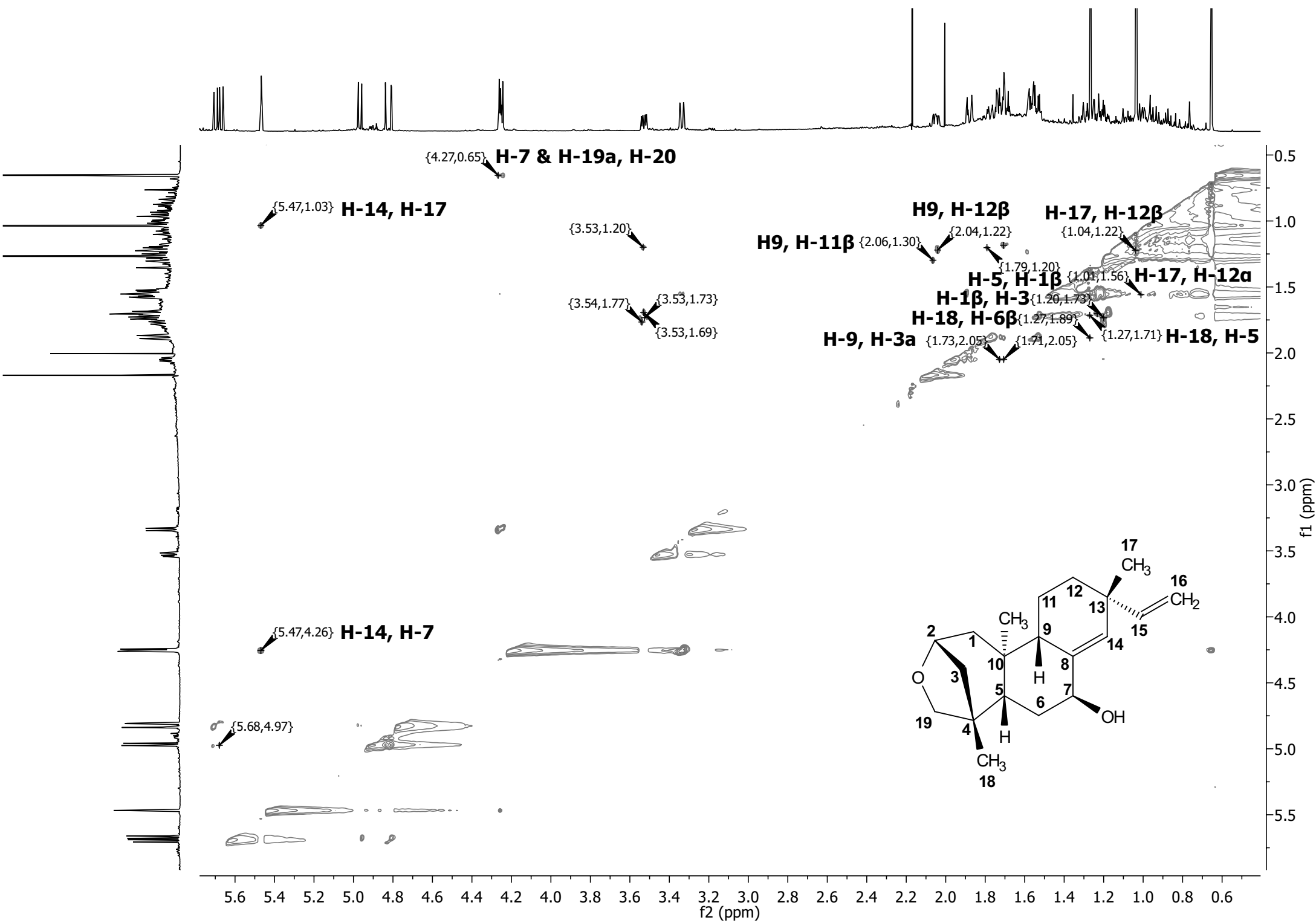

Supplement: Supplementary file 2 [file molecules-21-01237-s002.zip › Fig8_NOESY_S_13.pdf]

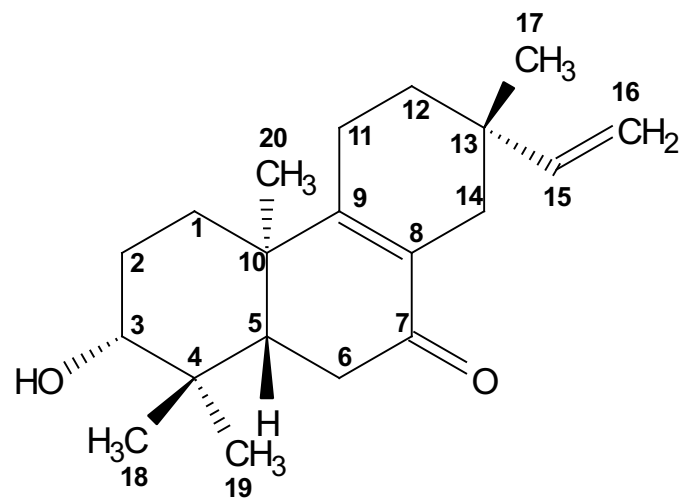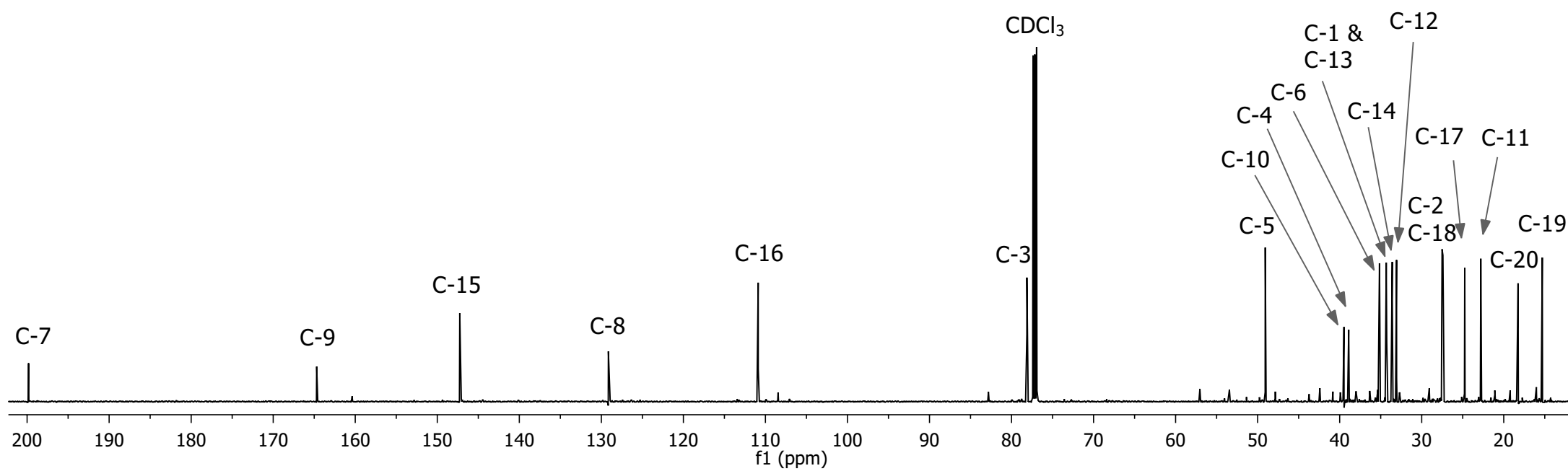

Supplement: Supplementary file 2 [file molecules-21-01237-s002.zip › Fig10_13C_NMR_S_14.pdf]

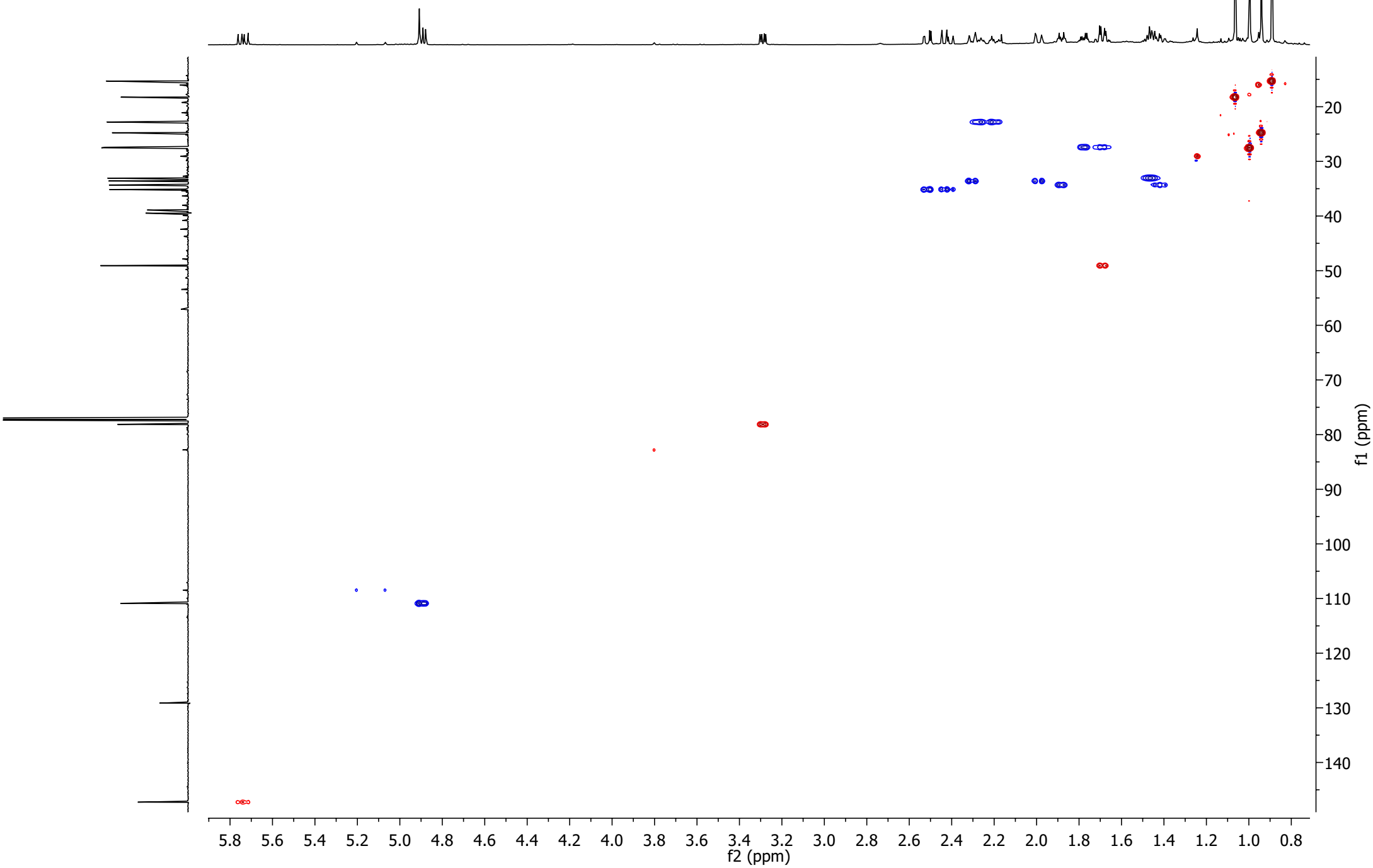

Supplement: Supplementary file 2 [file molecules-21-01237-s002.zip › Fig11_HSQC_S_14.pdf]

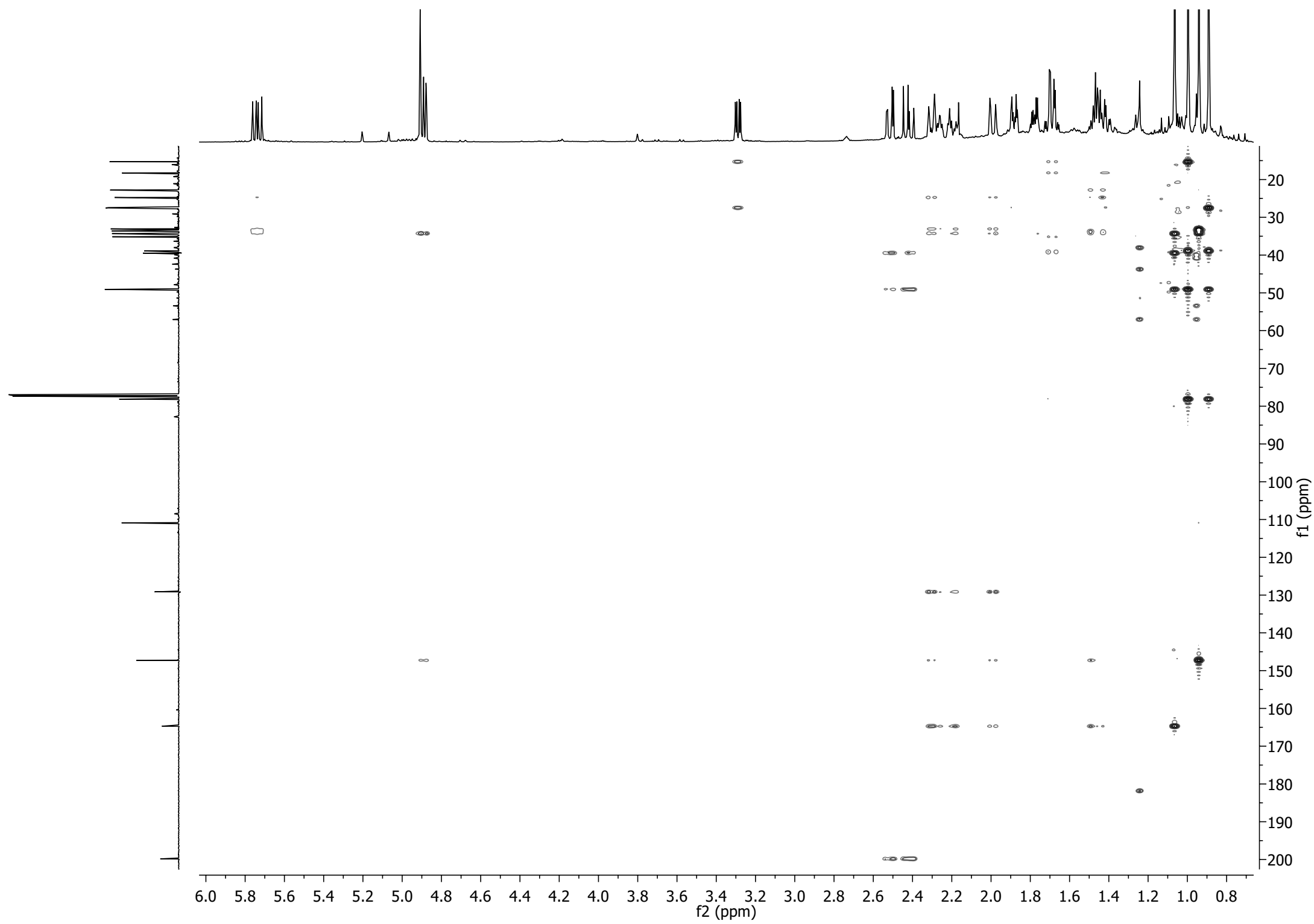

Supplement: Supplementary file 2 [file molecules-21-01237-s002.zip › Fig12_HMBC_S_14.pdf]

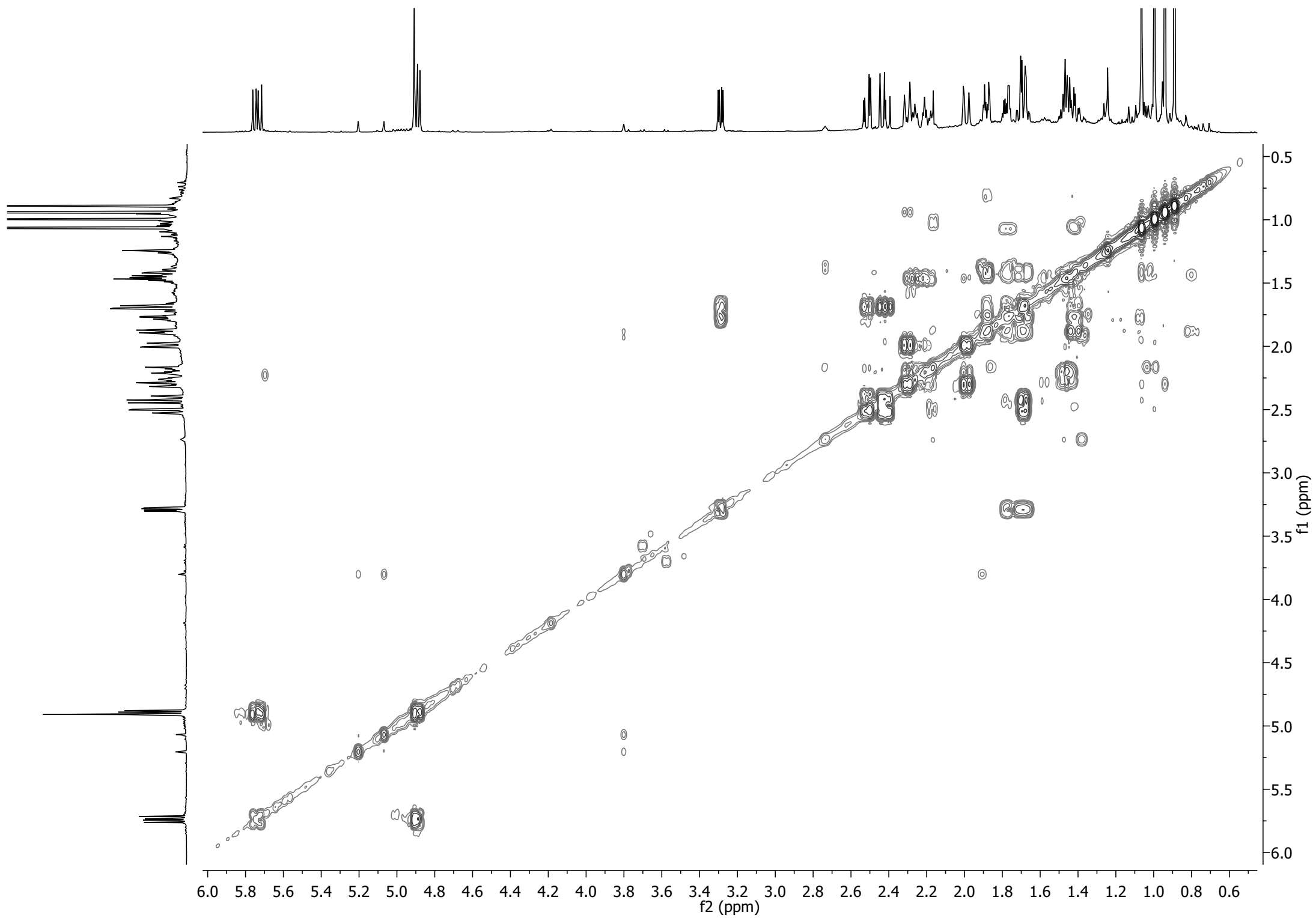

Supplement: Supplementary file 2 [file molecules-21-01237-s002.zip › Fig13_COSY_S_14.pdf]

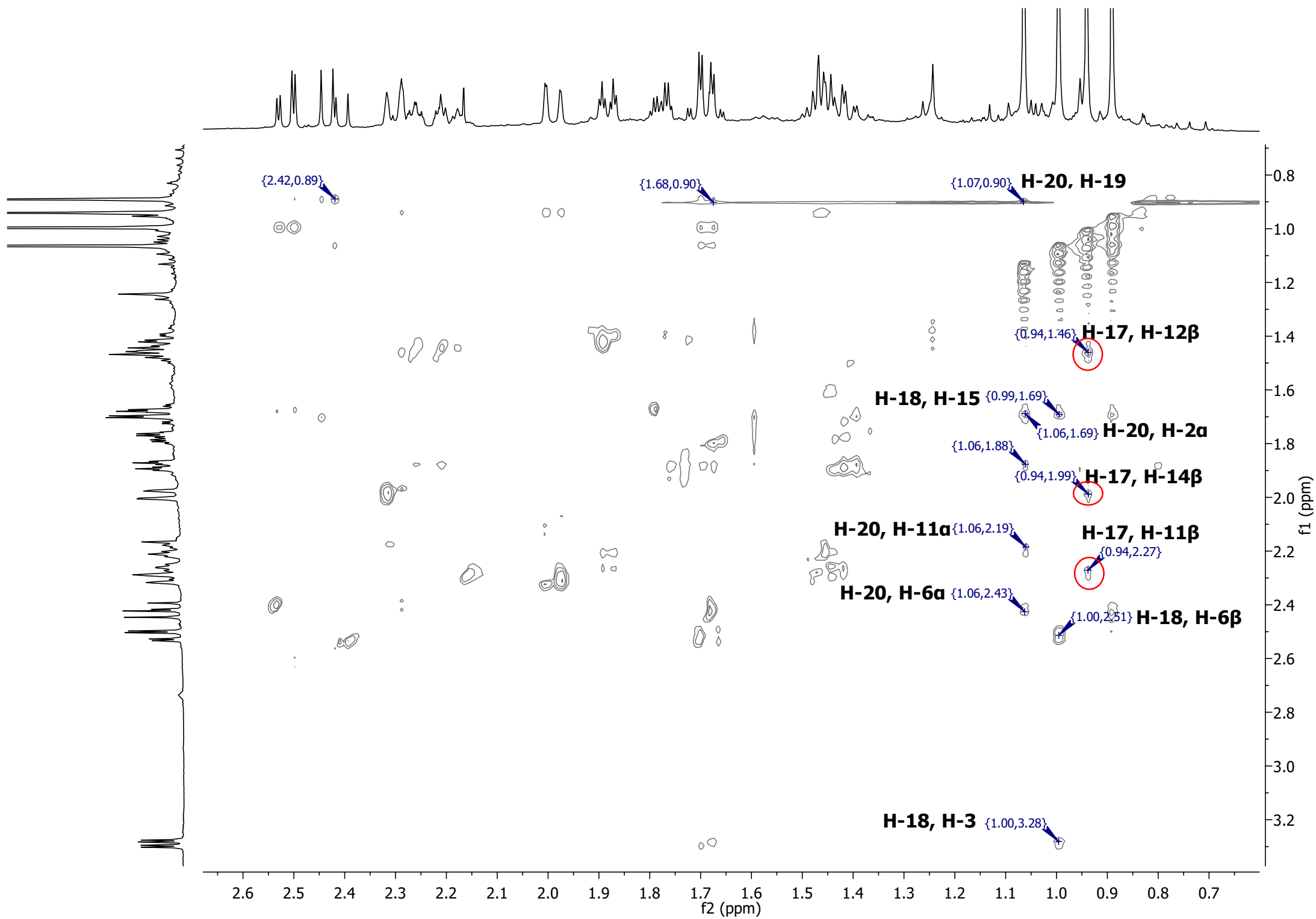

Supplement: Supplementary file 2 [file molecules-21-01237-s002.zip › Fig14_NOESY_S_14.pdf]

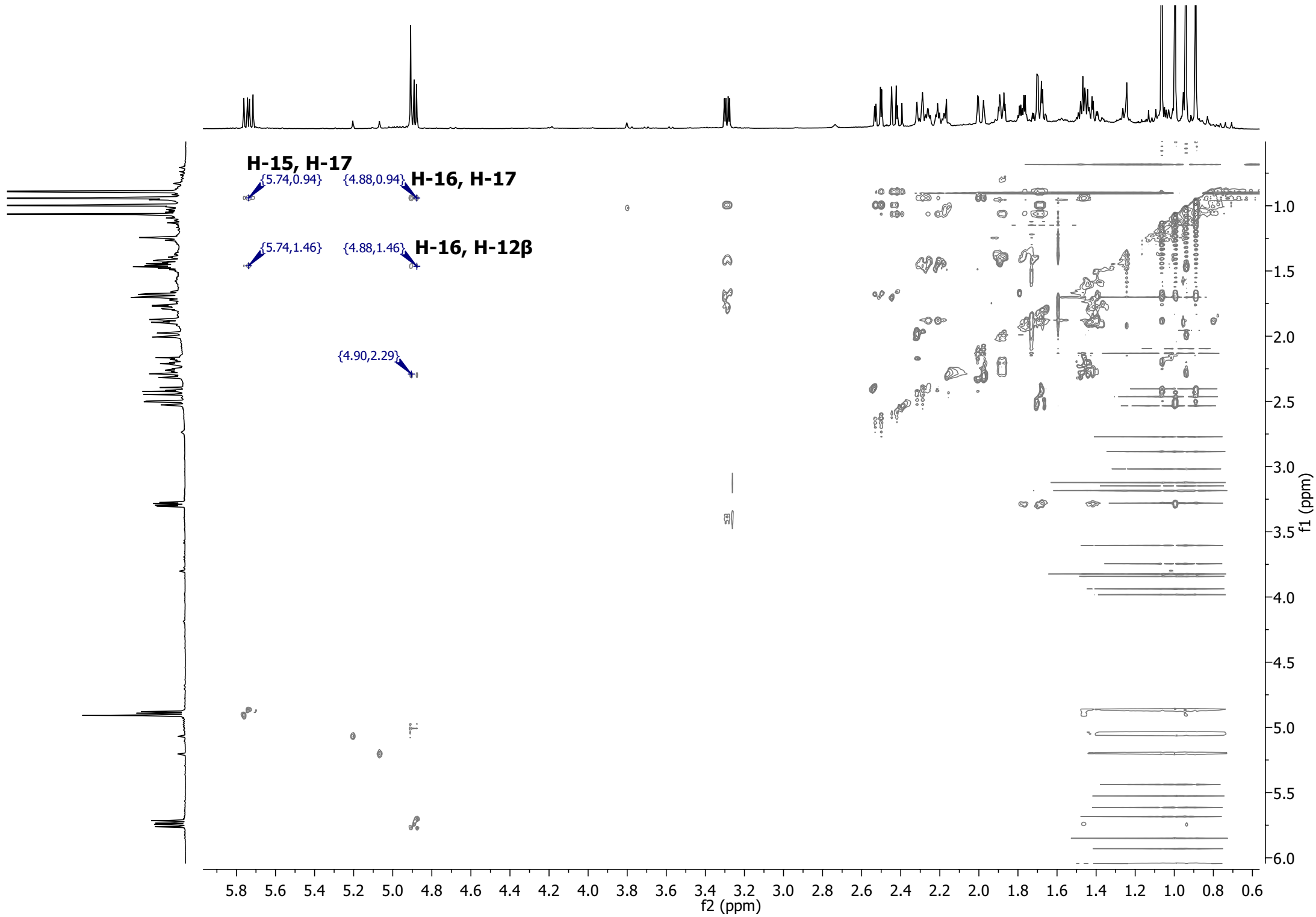

Supplement: Supplementary file 2 [file molecules-21-01237-s002.zip › Fig15_NOESY_S_14.pdf]

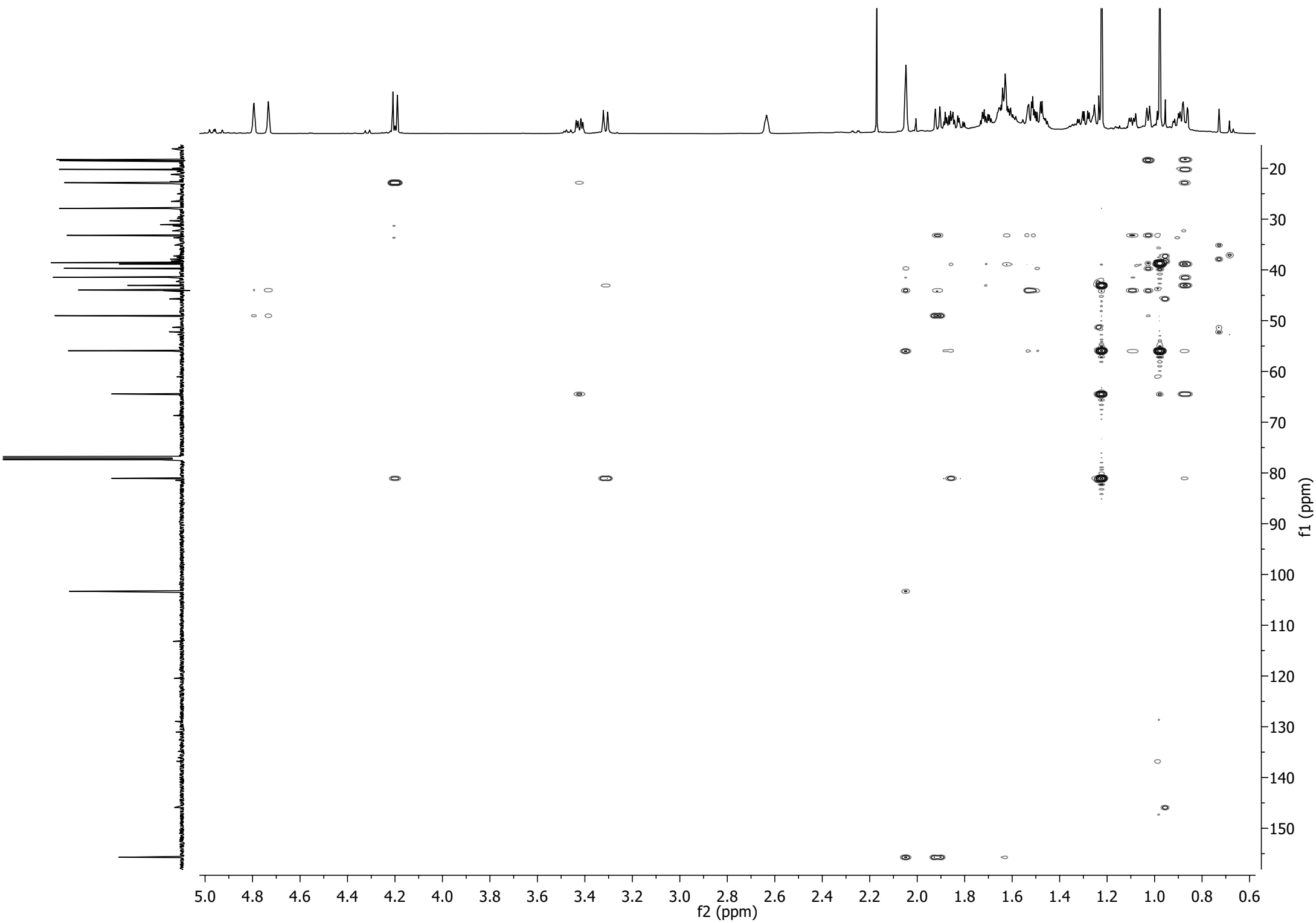

Supplement: Supplementary file 2 [file molecules-21-01237-s002.zip › HMBC-kaurane.pdf]

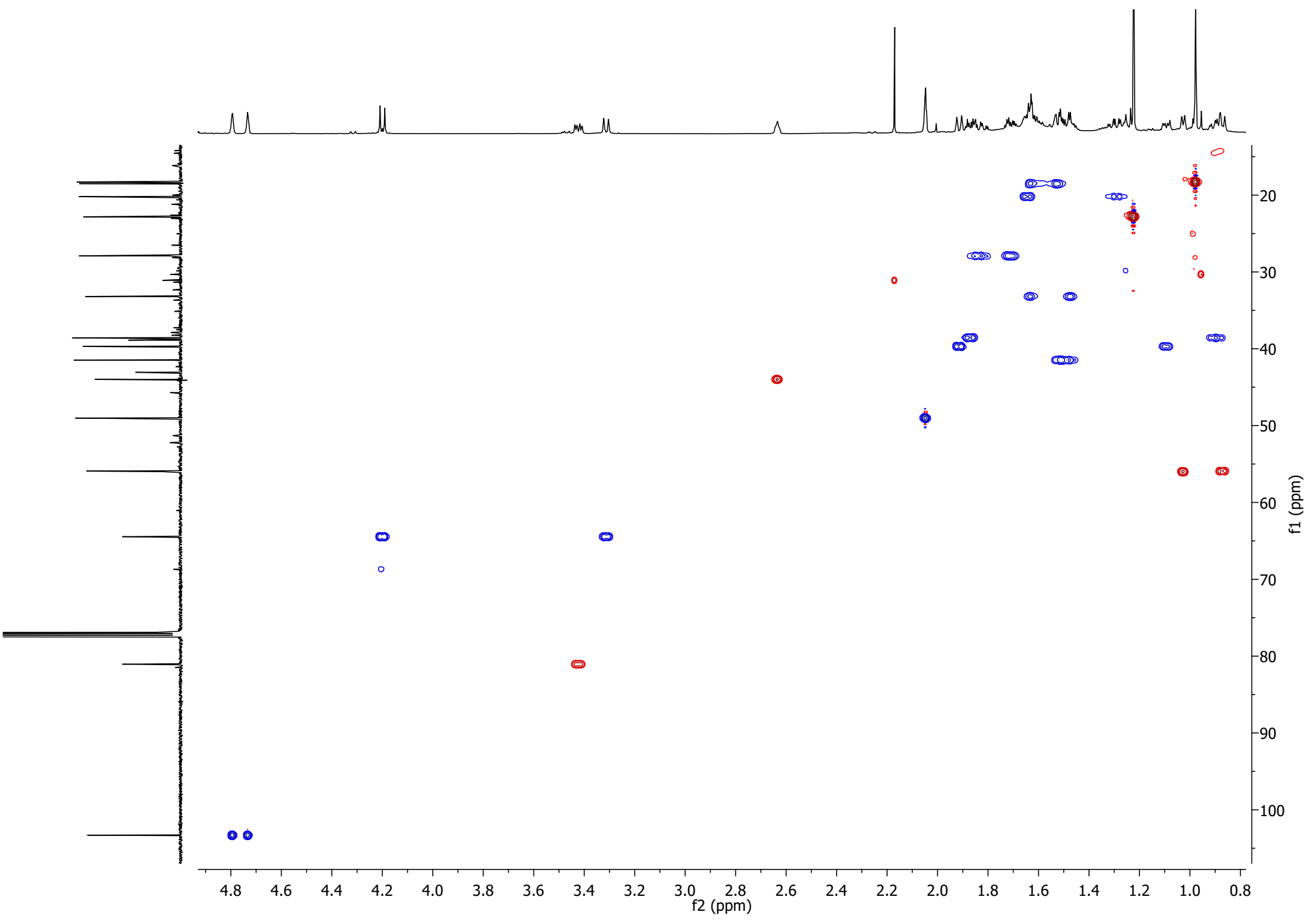

Supplement: Supplementary file 2 [file molecules-21-01237-s002.zip › HSQC-kaurane.pdf]

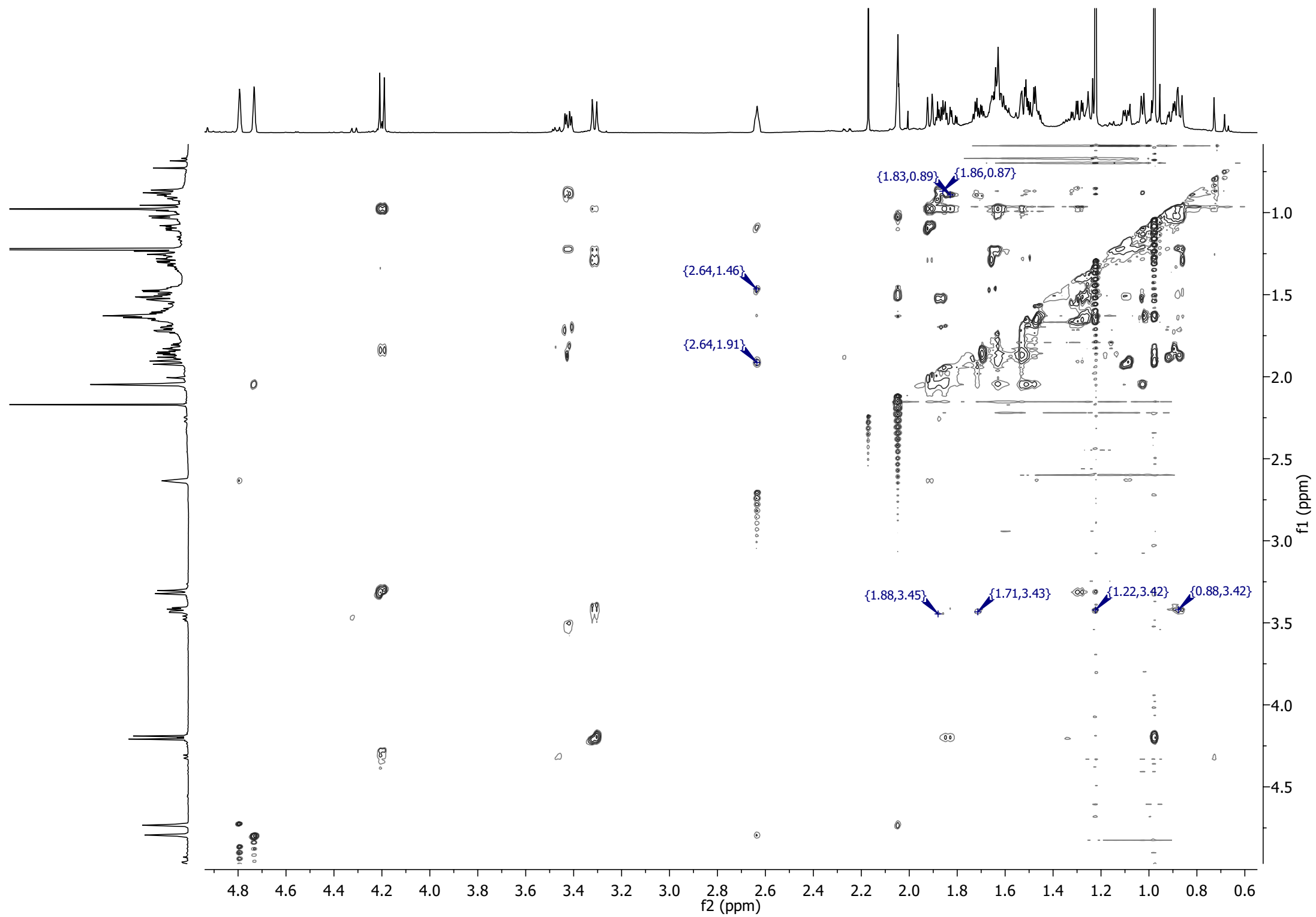

Supplement: Supplementary file 2 [file molecules-21-01237-s002.zip › NOESY-kaurane.pdf]

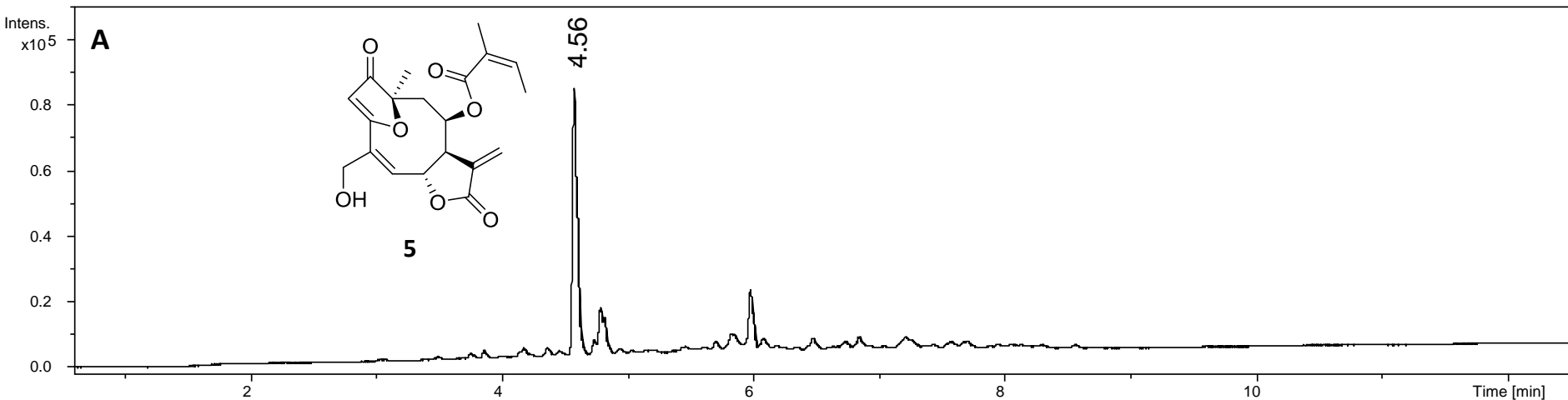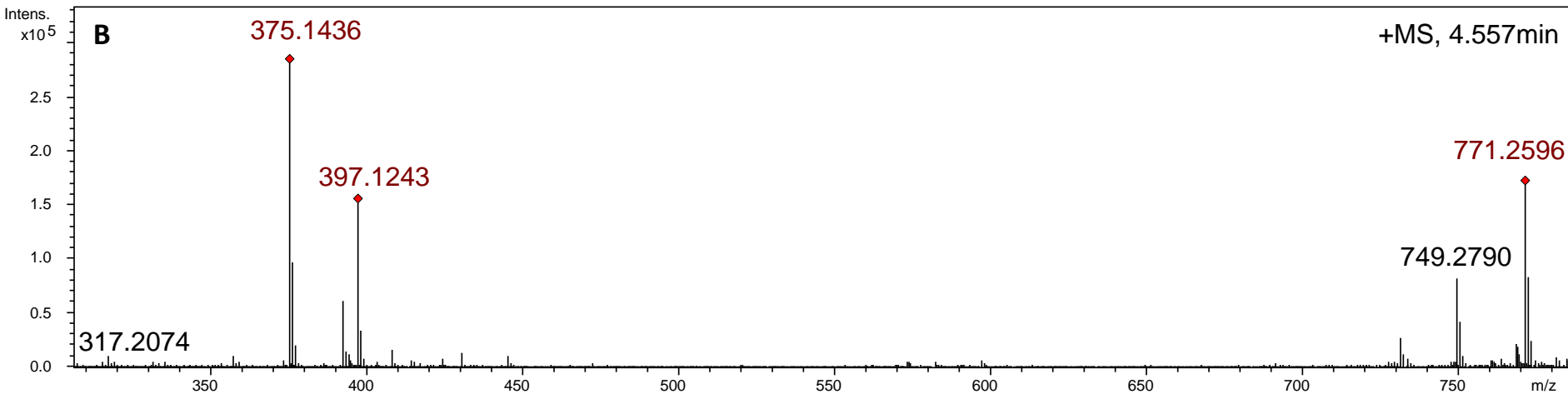

Supplement: Supplementary file 2 [file molecules-21-01237-s002.zip › Vd-Budlein-Chr-MS.pdf]

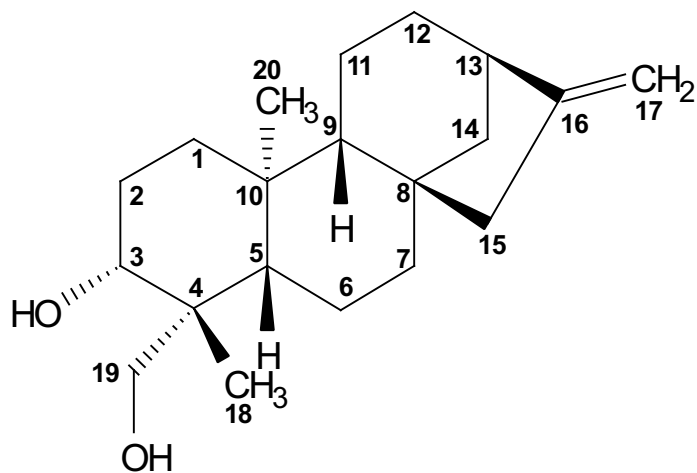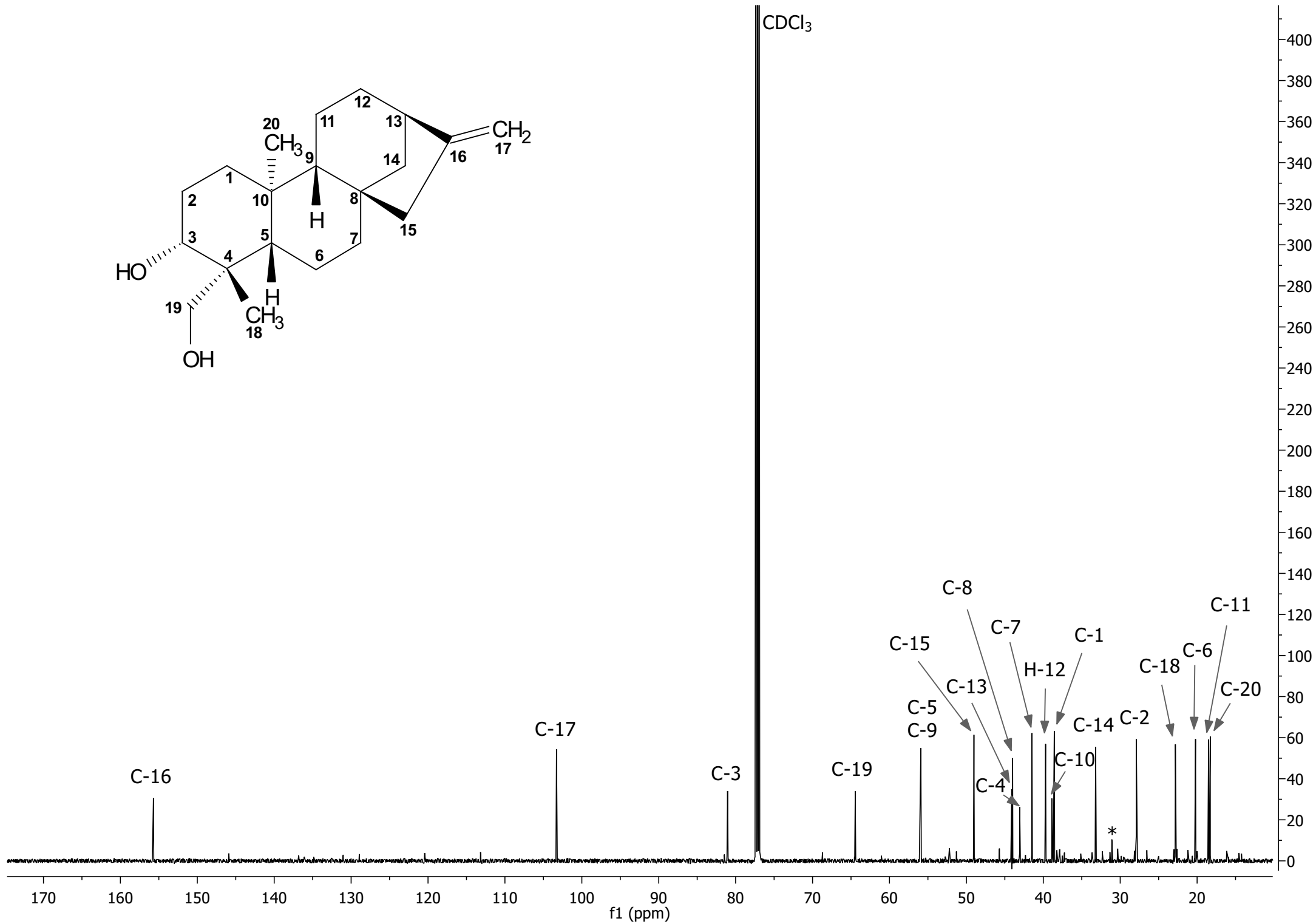

Supplement: Supplementary file 2 [file molecules-21-01237-s002.zip › 13C-kaurane.pdf]

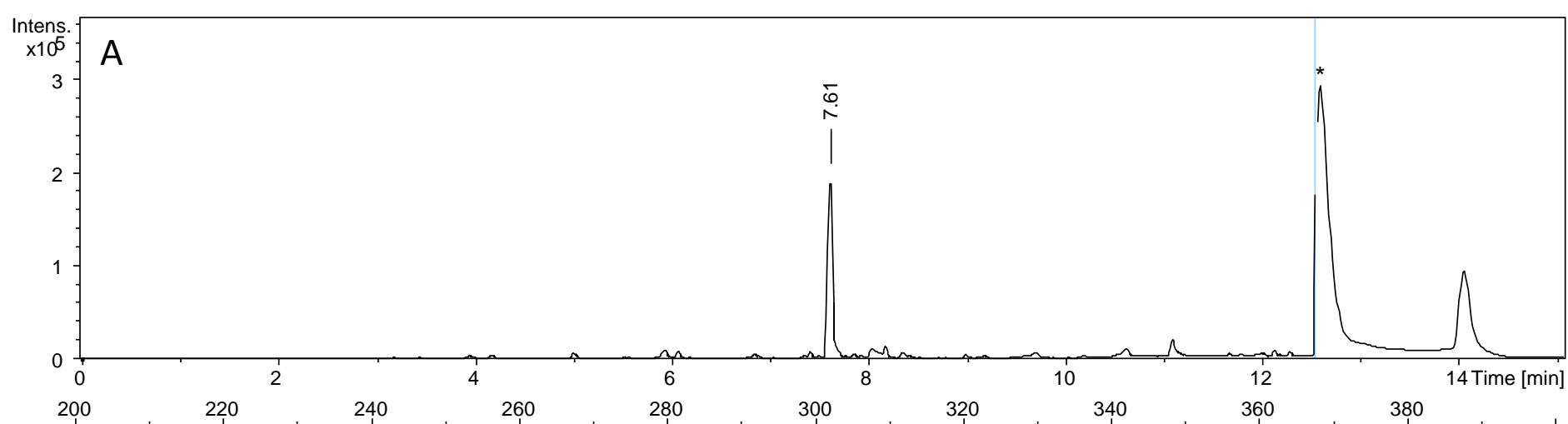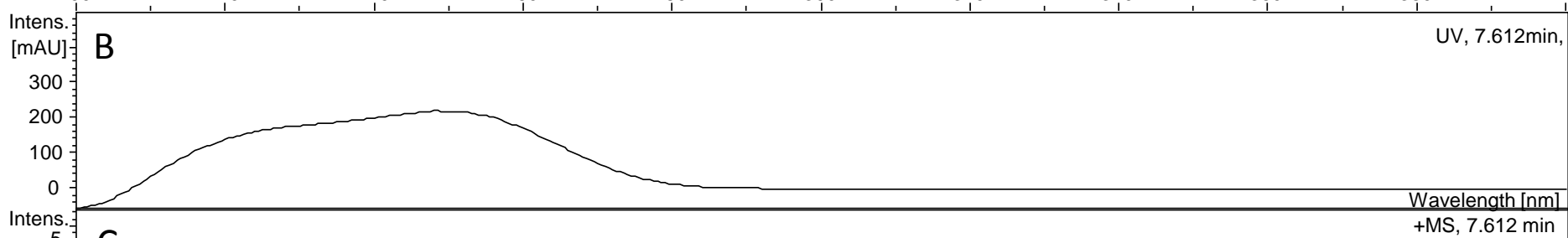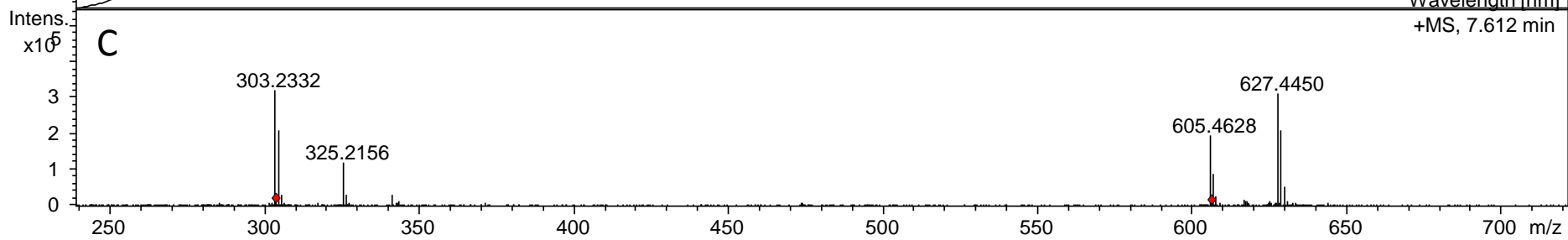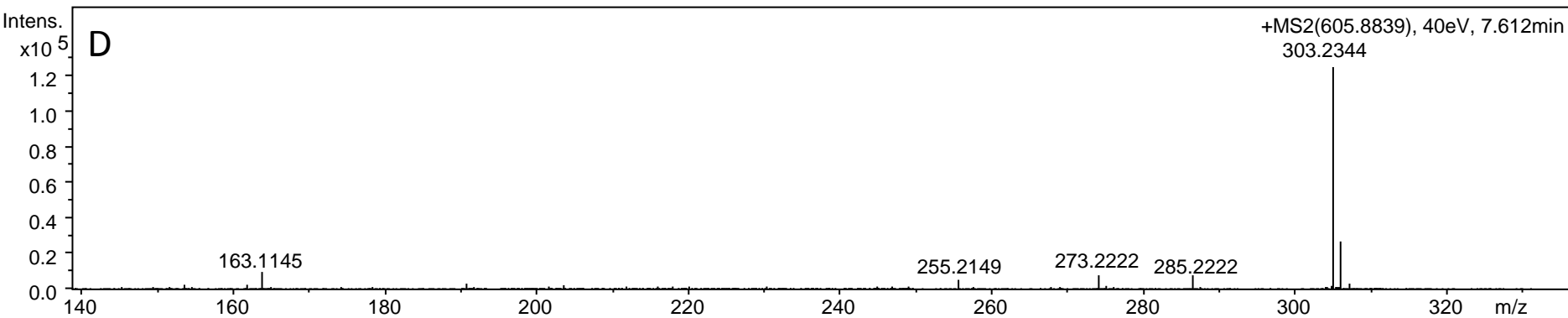

Supplement: Supplementary file 2 [file molecules-21-01237-s002.zip › Compound-1.pdf]

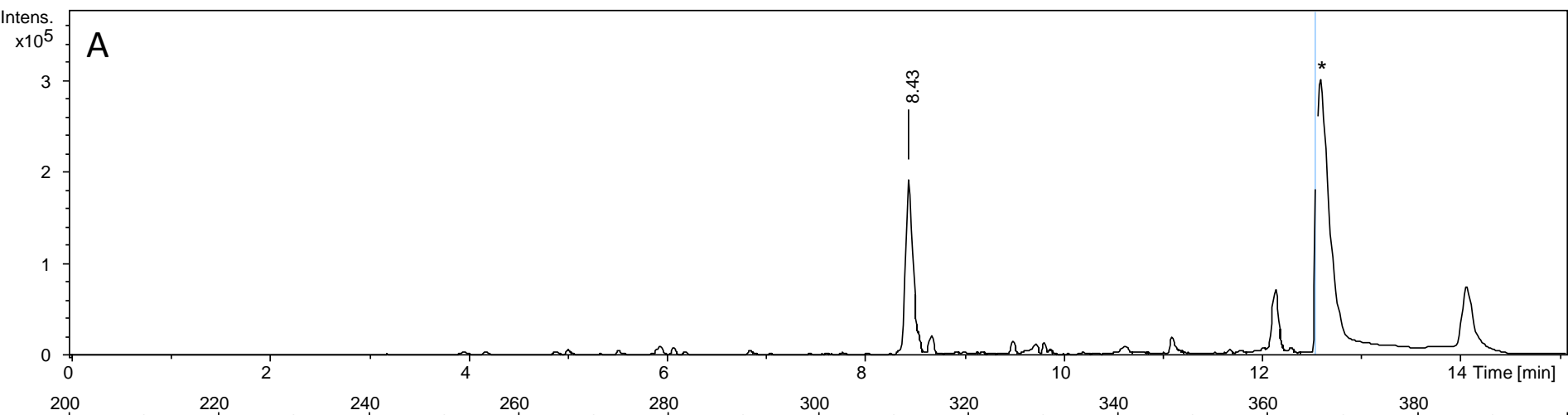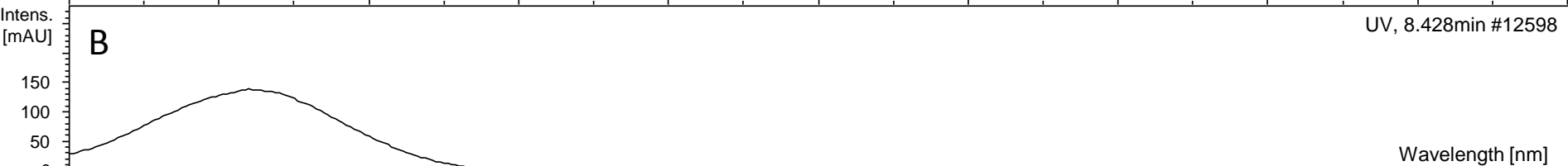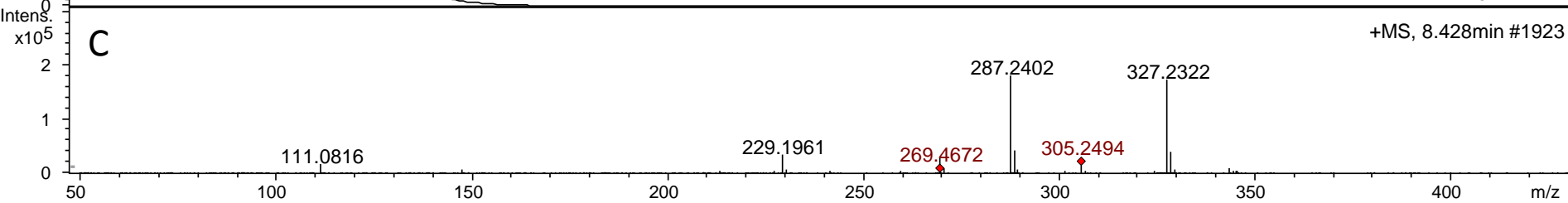

Supplement: Supplementary file 2 [file molecules-21-01237-s002.zip › Compound-2.pdf]

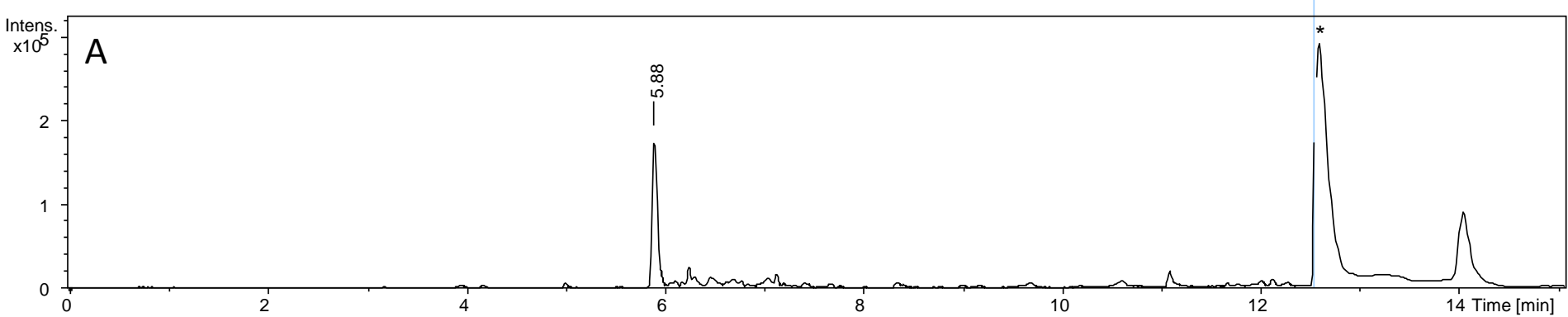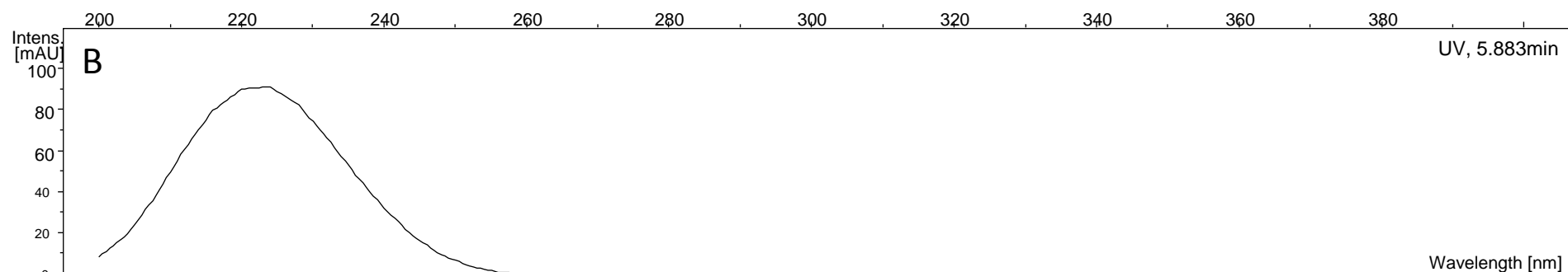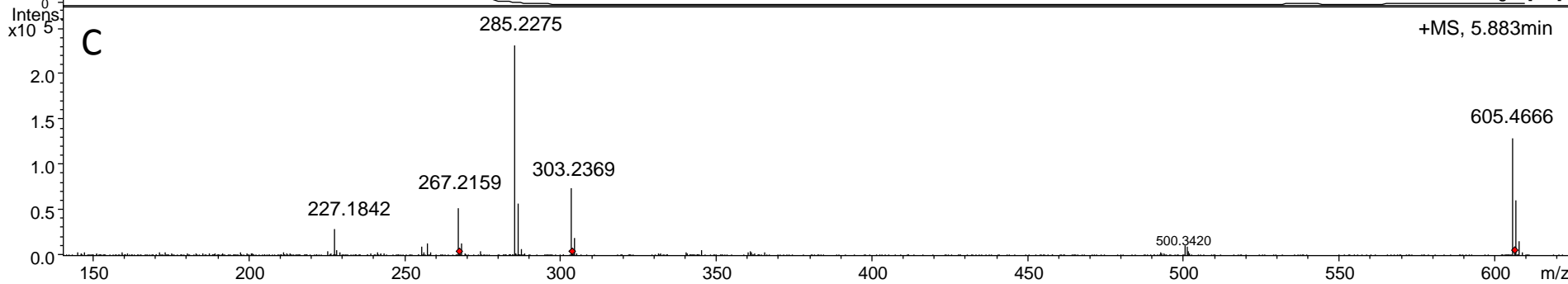

Supplement: Supplementary file 2 [file molecules-21-01237-s002.zip › Compound-3.pdf]

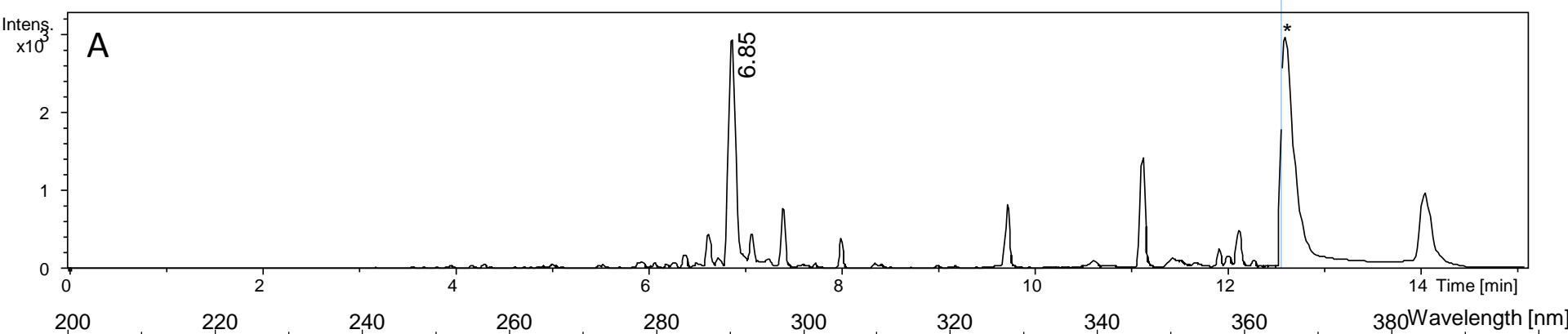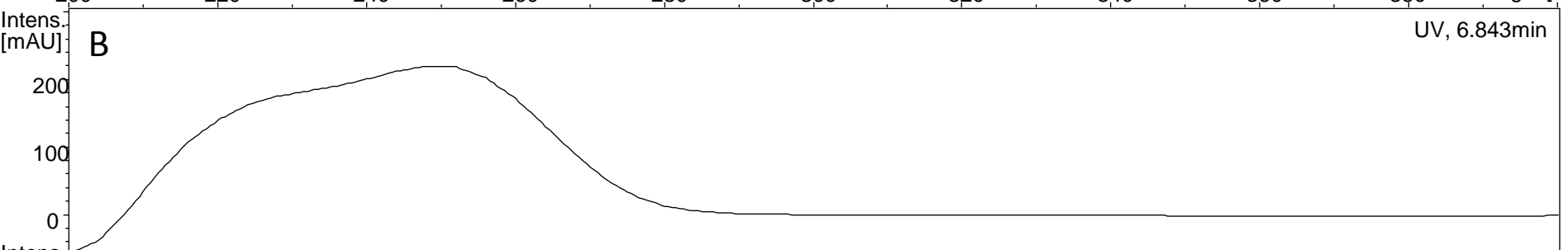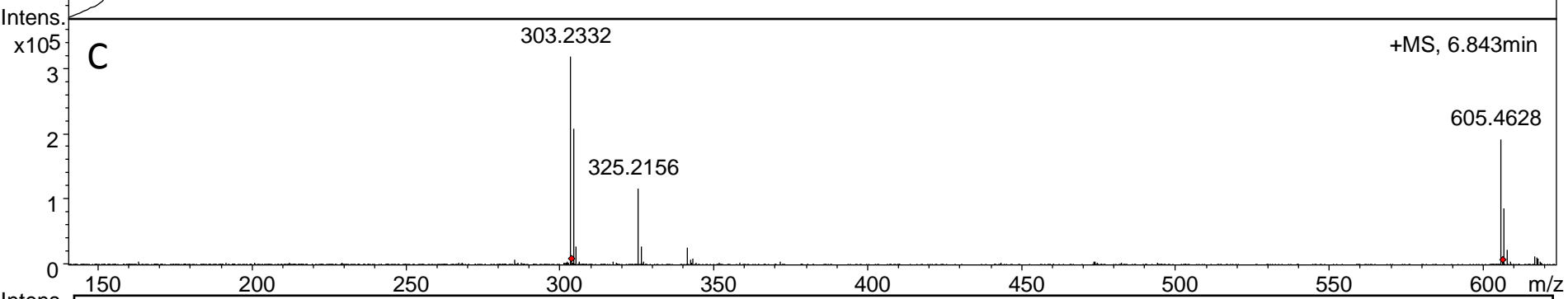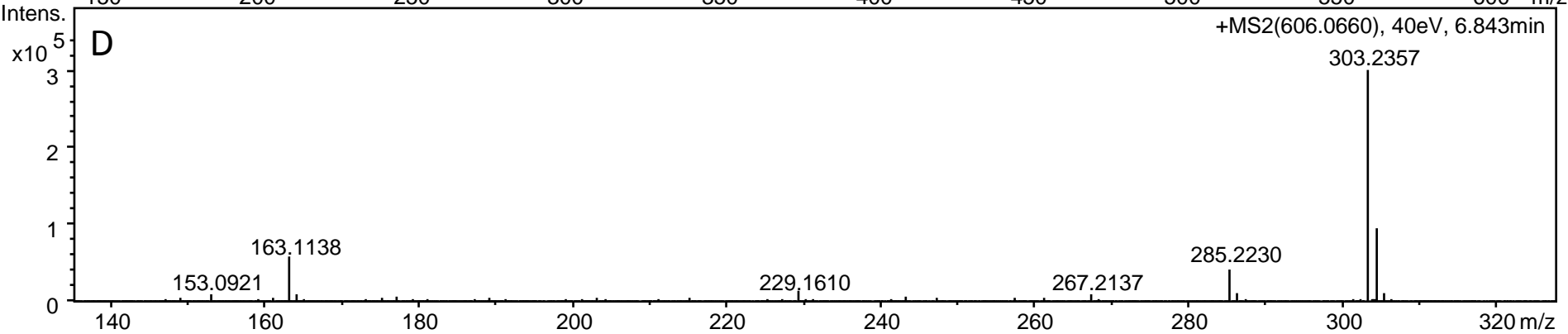

Supplement: Supplementary file 2 [file molecules-21-01237-s002.zip › Compound-4.pdf]

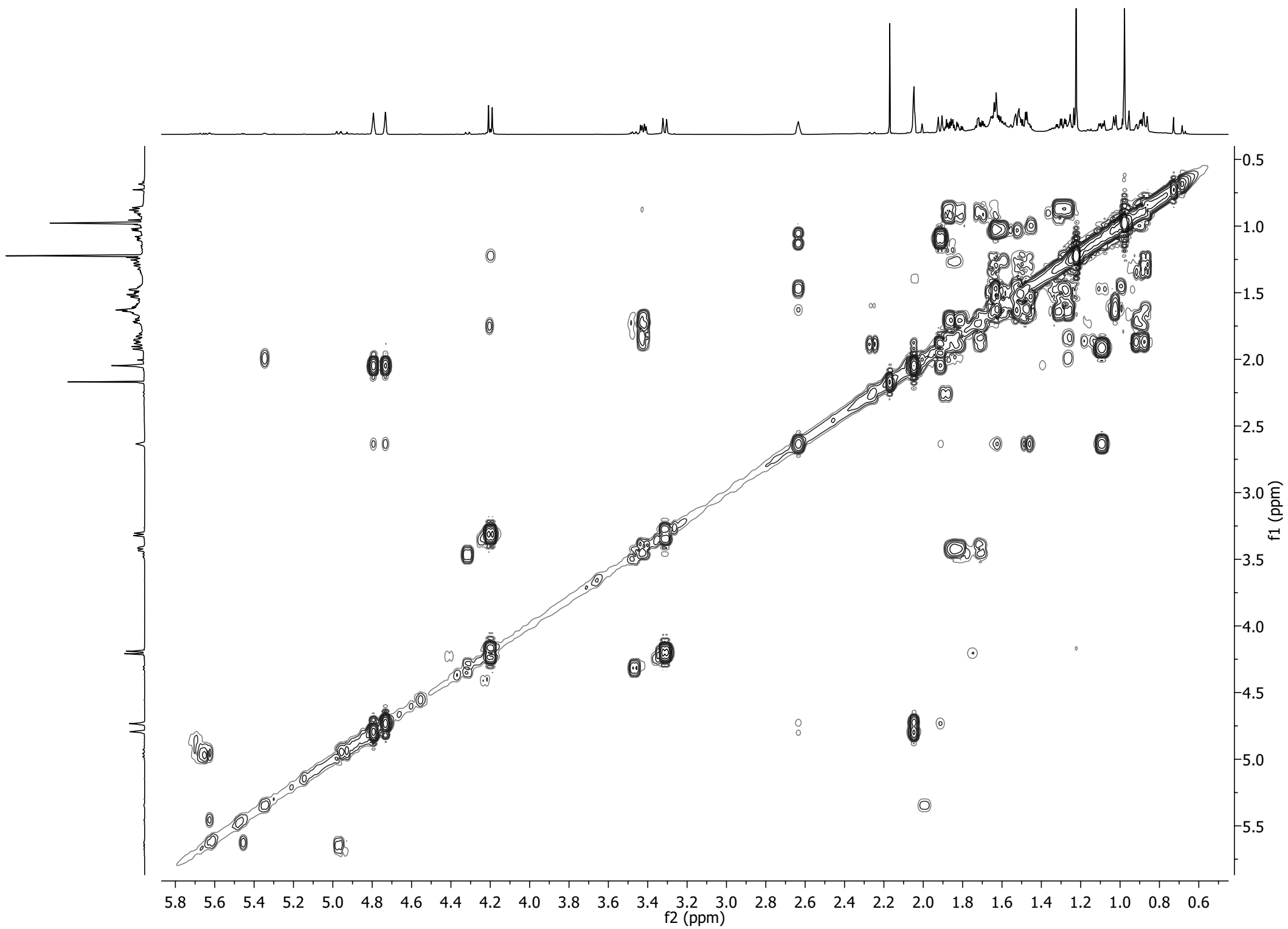

Supplement: Supplementary file 2 [file molecules-21-01237-s002.zip › COSY-kaurane.pdf]
